# Tumor suppressor genotype influences the extent and mode of immunosurveillance in lung cancer

**DOI:** 10.1038/s41467-026-74023-x

**Published:** 2026-06-15

**Authors:** Keren M. Adler, Haiqing Xu, Amy C. Gladstein, Valerie M. Irizarry-Negron, Maggie R. Robertson, Katherine R. Doerig, Dmitri A. Petrov, Monte M. Winslow, David M. Feldser

**Affiliations:** 1 Department of Cancer Biology, Perelman School of Medicine, University of Pennsylvania, Philadelphia, PA USA; 2 Abramson Family Cancer Research Institute, Perelman School of Medicine, University of Pennsylvania, Philadelphia, PA USA; 3 Cell and Molecular Biology Graduate Group, Perelman School of Medicine, University of Pennsylvania, Philadelphia, PA USA; 4 Department of Genetics, Stanford University School of Medicine, Stanford, CA USA; 5 Department of Biology, Stanford University School of Medicine, Stanford, CA USA; 6 Department of Pathology, Stanford University School of Medicine, Stanford, CA USA

**Keywords:** Immunoediting, Non-small-cell lung cancer

## Abstract

The impact of cancer driving mutations on immunosurveillance throughout tumor development remains poorly understood. To better understand the contribution of tumor genotype to immunosurveillance, we generated and validated lentiviral-based vectors that create increasingly immunogenic neoantigens. This vector system is compatible with autochthonous Cre-regulated cancer models, CRISPR/Cas9-mediated somatic genome editing, and tumor barcoding. Here, we show that in the context of oncogenic KRAS-driven lung cancer and strong neoantigen expression, tumor suppressor genotype dictates the degree of immune cell recruitment, positive selection of tumors with neoantigen silencing, and tumor outgrowth. By quantifying the impact of 11 commonly inactivated tumor suppressor genes on tumor growth across neoantigenic contexts, we show that the growth-promoting effects of tumor suppressor gene inactivation correlate with increasing sensitivity to immunosurveillance. Importantly, some genotypes also dramatically changed sensitivity to immunosurveillance independently of their growth-promoting effects. We propose a model of immunoediting in which tumor suppressor gene inactivation works in tandem with neoantigen expression to shape tumor immunosurveillance and immunoediting such that the same neoantigens uniquely modulate tumor immunoediting depending on the genetic context.

## Abstract (web summary)

The precise immune regulatory role of cancer driver mutations during tumor development remains unclear. Using lentiviral vectors to generate a tunable neoantigen library, this study reports that tumor suppressor genotype regulates immunosurveillance in KRAS-driven lung cancer.

## Introduction

Our understanding of cancer immune surveillance is colored by the immunoediting hypothesis, which proposes that in response to neoantigens presented on cancer cells, the adaptive immune system imposes a selective pressure that drives tumor evolution towards a more immune-evasive state^1,2^. In the decades since the inception of the immunoediting hypothesis, multiple mechanisms have emerged to explain how cancer cells actively promote an immunosuppressive tumor microenvironment that limits the immune system’s ability to identify and eliminate antigen-expressing cancer cells^3–9^. Interestingly, the precise genetic determinants of malignant transformation itself (*i.e*., oncogene acquisition and tumor suppressor gene inactivation) can regulate cell-intrinsic immune modulatory pathways and even predict the response to immune checkpoint therapy in certain contexts^10–20^. However, the extent to which cancer genotype shapes immune surveillance and whether different genotypes increase or decrease immunoediting remains unclear.

Autochthonous cancer models that recapitulate tumor initiation and early expansion driven by oncogene activation and tumor suppressor gene inactivation represent tractable model systems to understand gene-microenvironment interactions in a physiologically relevant context^21,22^. Recent advances in CRISPR-mediated gene inactivation in somatic cells, coupled with tumor barcoding, have enabled the quantitative analysis of the effects of different genes on cancer cell fitness^23^. Here, we combine genetically engineered conditional mouse models with lentiviral-mediated somatic gene inactivation, neoantigen expression, and tumor barcoding to better understand how inactivation of diverse tumor suppressor genes modulates immunoediting. By expressing fixed neoantigens across different genetic contexts, we shift the focus away from the role of the neoantigen in immunoediting and towards the importance of the genetic context in which the neoantigen arises. Our methodology is a resource for the assessment of tumor genotype-immune phenotype interactions in a highly quantitative and relatively high-throughput in vivo manner. Our study uncovers tumor genotype-dependencies that impact mechanisms of immune evasion as well as sensitivity or resistance to immune surveillance, thus highlighting a previously under-appreciated role for cancer driver mutations in shaping immunoediting.

## Results

### A system to generate an immunogenic series of lung tumors in vivo

**Fig. 1:**
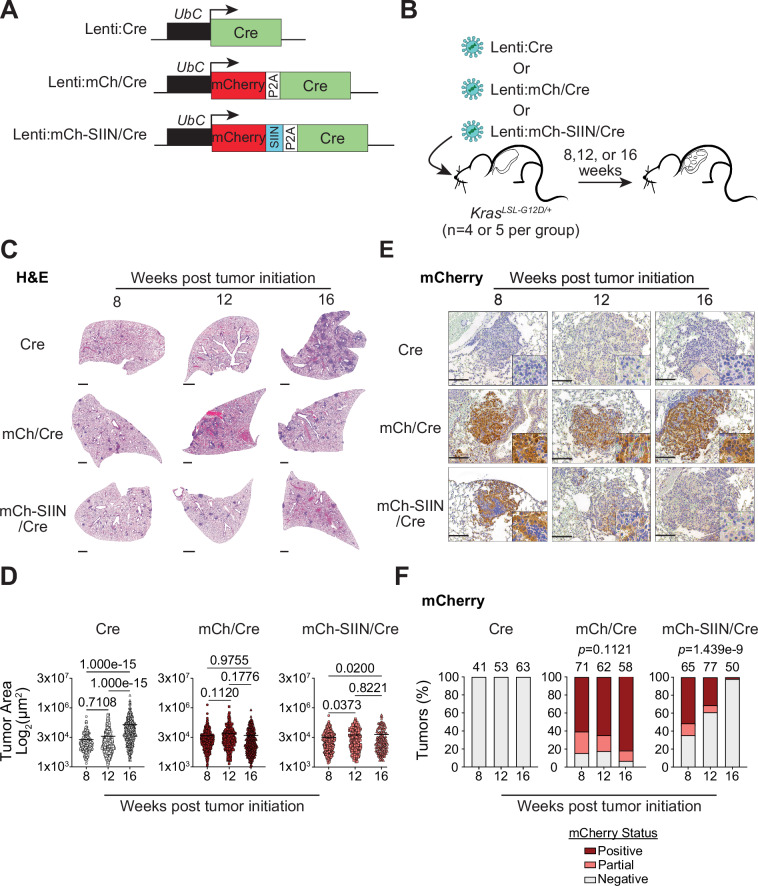
An immunogenic series of lentiviral-based vectors to study tumor immunosurveillance in KRAS-driven lung tumors. **A**Depiction of immunogenic series of lenti-vectors used to initiate tumors. Top, Lenti:Cre: baseline vector expresses Cre recombinase from the UbC promoter. Middle, Lenti:mCh/Cre: moderately immunogenic vector expresses mCherry and Cre recombinase from the UbC promoter. Bottom, Lenti:mCh-SIIN/Cre: highly immunogenic vector expresses an mCherry-SIINFEKL fusion protein as well as Cre recombinase from the UbC promoter. **B** Experimental outline. Tumors were initiated in *Kras*^*LSL-G12D/+*^ mice using 1×10^5^ plaque-forming units (PFU) of Lenti:Cre, Lenti:mCh/Cre, or Lenti:mCh-SIIN/Cre. Lungs were harvested for sectioning at 8, 12, or 16 weeks post-transduction. **C** Representative scans of H&E stained lungs from each experimental group. Tile scans were taken at 5x magnification. Scale bar is 1 mm. **D** Quantification of individual tumor area for each vector at 8, 12, and 16 weeks, graphed using a log_2_ scale. Statistical significance was determined using one-way ANOVA testing with a Šidák correction. Error bars represent mean ± standard deviation. For Lenti:Cre, *n* = 5 mice at 8 weeks post tumor initiation and *n* = 4 mice at 12 and 16 weeks post tumor initiation. For Lenti:mCh/Cre, *n* = 4 mice at 8, 12, and 16 weeks post tumor initiation. For Lenti:mCh-SIIN/Cre, n = 5 mice at 8 and 12 weeks post tumor initiation and *n* = 4 mice at 16 weeks. **E** Representative IHC images for mCherry at 8, 12, and 16 weeks after tumor initiation with Lenti:Cre, Lenti:mCh-Cre, or Lenti:mCh-SIIN/Cre. Insets are 3x magnified. Scale bar is 119um. No independent repetitions of this staining were conducted. **F** The proportion of mCherry^Positive^ tumors was assessed 8, 12, and 16 weeks after tumor initiation across all three immunogenic contexts and plotted as percentages in stacked bar plots. Number of individual tumors analyzed for each group is indicated on graph. For Lenti:Cre, *n* = 5 mice at 8 weeks, *n* = 4 mice at 12 and 16 weeks. For Lenti:mCh/Cre, *n* = 4 mice at 8, 12, and 16 weeks. For Lenti:mCh-SIIN/Cre, *n* = 5 mice at 8 and 12 weeks, and *n* = 4 mice at 16 weeks. Statistical significance of mCherry expression was determined using a chi-squared test.

To model immunoediting during lung tumor formation, we used the *Kras*^*LSL-G12D/+*^ (*K*) genetically engineered mouse model in which tumors are initiated via transduction of lung epithelial cells with lentiviral-based vectors expressing Cre recombinase^21^. To promote immunogenicity in the lung, we generated a lentiviral-based vector that expresses the MHC-I restricted peptide SIINFEKL linked to mCherry in addition to Cre (Lenti:mCh-SIIN/Cre, Fig. 1A). Thus, mCherry and mCherry-SIINFEKL expression serve as a simple readout for neoantigen expression in tumors and provide a tractable means to model neoantigen-driven tumor-immune dynamics in Genetically Engineered Mouse Models (GEMMs) of lung adenocarcinoma that otherwise exhibit low mutational burden and limited immunogenicity^3,24,25^. To model a less immunogenic tumor state, a similar vector was designed to only express mCherry and Cre (Lenti:mCh/Cre, Fig. 1A). Lastly, to control for the potential immunogenic effects of mCherry, a third vector was generated that only expresses Cre (Lenti:Cre, Fig. 1A). Tumors were initiated in *K* mice with either Lenti:Cre, Lenti:mCh/Cre, or Lenti:mCh-SIIN/Cre, and analyzed 8, 12, and 16 weeks after tumor initiation (Fig. 1B). Mice transduced with each vector had numerous small adenomas 8 weeks after tumor initiation (Fig. 1C). As expected, tumors initiated with Lenti:Cre increased significantly in size over time (Fig. 1C and D). In contrast, tumors initiated with either Lenti:mCh/Cre or Lenti:mCh-SIIN/Cre grew at a slower rate. Immunohistochemical (IHC) staining for mCherry showed that the vast majority of tumors (93%) initiated with Lenti:mCh/Cre were mCherry^Positive^ throughout the 16-week time course (Fig. 1E and F). However, while the majority of tumors initiated with Lenti:mCh-SIIN/Cre (64%) were mCherry^Positive^ 8 weeks after tumor initiation, a progressively smaller percent of tumors expressed mCherry at 12 (39%) and 16 (2%) weeks after tumor initiation (Fig. 1E and F).

To validate that the loss of mCherry-SIINFEKL expression was due to a SIINFEKL-mediated T cell response, we initiated tumors in *Kras*^*LSL-G12D/+*^*;Rag1*^−/−^ (*K;Rag1*^−/−^) mice. 12 weeks after tumor initiation, Lenti:mCh/Cre and Lenti:mCh-SIIN/Cre initiated tumors were similar in size in *K;Rag1*^−/−^ mice, whereas Lenti:mCh-SIIN/Cre initiated tumors were significantly smaller than Lenti:mCh/Cre initiated tumors in immunocompetent *K;Rag1*^*+/+*^ mice (Fig. S1A). Additionally, unlike in *K* mice, the proportion of mCherry^Positive^ tumors in *K;Rag1*^−/−^ mice was the same whether tumors were initiated by Lenti:mCh/Cre or Lenti:mCh-SIIN/Cre (Figure S1B). Thus, as expected, mCherry-SIINFEKL expression forces T cell-dependent immune surveillance that limits tumor growth and selects for tumors with silenced neoantigen expression.

**Fig. 2:**
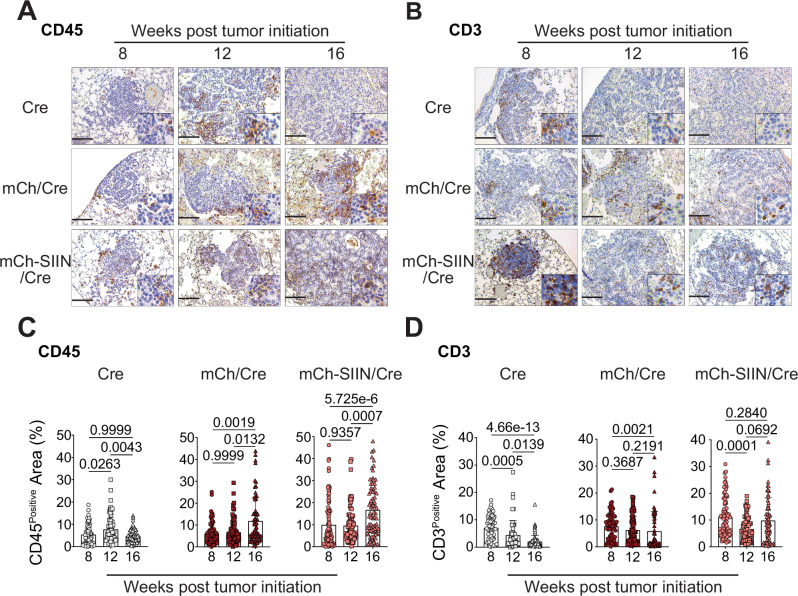
SIINFEKL expression induces an immune response in KRAS-driven lung tumors. **A**,** B** Representative 20x IHC for the immune markers CD45 (A) and CD3 (B) at 8, 12, or 16 weeks post-tumor initiation in tumors initiated by Lenti: Cre, Lenti:mCh/Cre, or Lenti:mCh-SIIN/Cre. Insets are 3x magnified. Scale bar is 119um. No independent repetitions of this staining were conducted. **C** Quantification of CD45 IHC shown in Fig. 2A using percent positive area. Statistical significance was determined using a Kruskal-Wallis test. Error bars represent mean ± standard deviation. For Lenti: Cre, *n* = 77 tumors from *n* = 5 mice at 8 weeks, *n* = 71 tumors from *n* = 4 mice at 12 weeks, and *n* = 80 tumors from *n* = 4 mice at 16 weeks. For Lenti:mCh/Cre, *n* = 84 tumors from *n* = 4 mice at 8 weeks, *n* = 81 tumors from *n* = 4 mice at 12 weeks, and *n *= 65 tumors from *n* = 4 mice at 16 weeks. For Lenti:mCh-SIIN/Cre, *n* = 93 tumors from *n* = 5 mice at 8 weeks, *n* = 84 tumors from *n* = 5 mice at 12 weeks, and *n* = 80 tumors from *n* = 4 mice at 16 weeks. **D** Quantification of CD3 IHC shown in Fig. 2B using percent positive area. Statistical significance was determined using a Kruskal-Wallis test. Error bars represent mean ± standard deviation. For Lenti: Cre, *n* = 71 tumors from *n* = 5 mice at 8 weeks, *n *= 52 tumors from *n* = 4 mice at 12 weeks, and *n* = 86 tumors from *n* = 4 mice at 16 weeks. For Lenti:mCh/Cre, *n* = 72 tumors from *n* = 4 mice at 8 weeks, *n* = 62 tumors from *n* = 4 mice at 12 weeks, and *n *= 61 tumors from *n* = 4 mice at 16 weeks. For Lenti:mCh-SIIN/Cre, *n* = 86 tumors from *n* = 5 mice at 8 weeks, *n* = 67 tumors from *n* = 5 mice at 12 weeks, and n = 66 tumors from *n* = 4 mice at 16 weeks.

To determine the extent to which each immunogenic condition promotes immune cell infiltration, we performed IHC (Fig. 2A and B). Tumors initiated with Lenti:Cre had relatively few infiltrating CD45^Positive^ cells that remained constant throughout the 8-, 12-, and 16-week time points after tumor initiation (Fig. 2C, Fig. S1C). In tumors initiated with Lenti:mCh/Cre CD45^Positive^ cell infiltration was low at early time points after tumor initiation but increased significantly by 16 weeks. Tumors initiated by Lenti:mCh-SIIN/Cre had a higher frequency of CD45^Positive^ cells 8 weeks after tumor initiation compared to both Lenti:Cre (*p* = 0.0005) and Lenti:mCh/Cre (*p* = 0.0024) initiated tumors that persisted throughout the time course. The degree of T cell infiltration (CD3^Positive^) was distinct from total immune cell infiltration during the time course. In Lenti:Cre initiated tumors, T cell infiltration declined significantly over time whereas in Lenti:mCh/Cre and in Lenti:mCh-SIIN/Cre initiated tumors, T cell infiltration was initially high and persisted over time (Fig. 2D, Fig. S1D). The high level of immune infiltration in tumors initiated with Lenti:mCh/Cre late, and Lenti:mCh-SIIN/Cre through the 16-week time point is consistent with their limited expansion compared to tumors initiated with Lenti:Cre (Fig. 1C, D). Taken together with the analysis of mCherry expression, these data suggest that while mCherry expression alone elicits an immune response strong enough to restrict tumor outgrowth, it is insufficiently immunogenic to select for the outgrowth of cells with neoantigen silencing. However, mCherry-SIINFEKL expression is sufficiently potent to drive rapid selection of tumor cells that have silenced neoantigen expression. Thus, Lenti:Cre, Lenti:mCh/Cre, and Lenti:mCh-SIIN/Cre represent a series of vectors that can be used to initiate autochthonous lung tumors with increasing immunogenicity in vivo.

### Tumor suppressor genotype differentially regulates immunoediting

**Fig. 3:**
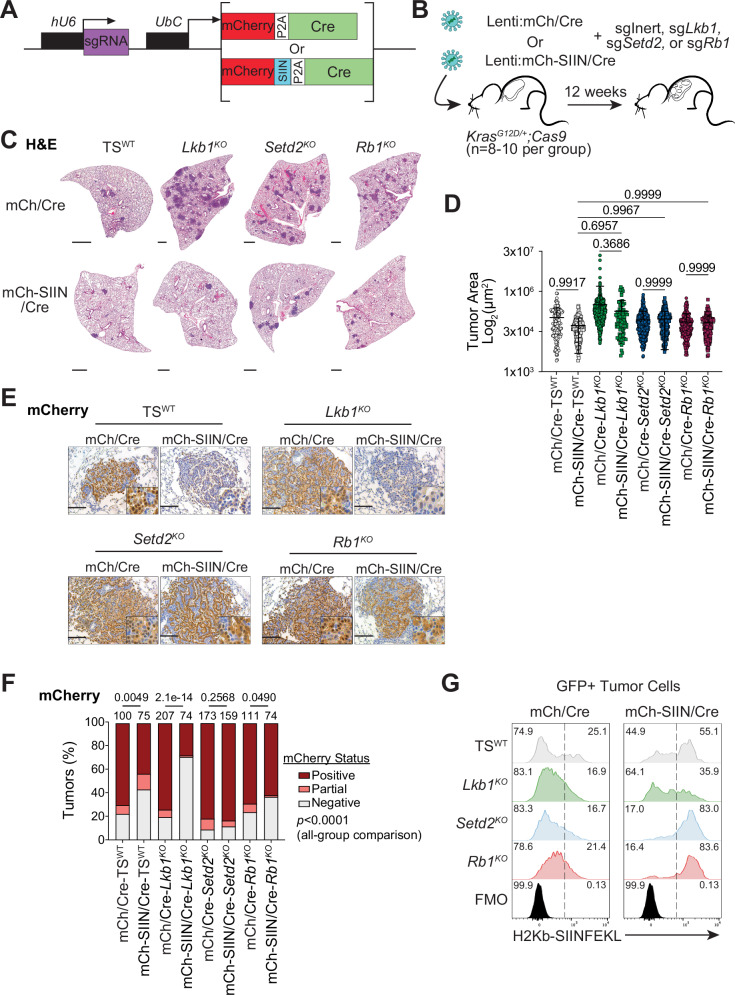
Tumors with *Setd2*^*KO*^ or *Rb1*^*KO*^, but not *Lkb1*^*KO*^, maintain SIINFEKL expression and presentation 12 weeks after tumor initiation. **A** Design of immunogenic series of lenti-vectors used to simultaneously initiate tumors and knockout a gene of interest. Design is similar as to what is depicted in Fig. 1A, with the addition of a human U6 promoter driving the expression of a sgRNA of interest. **B** Experimental scheme. Tumors were initiated in *K;Cas9* mice with 6×10^4^ PFU of Lenti:mCh/Cre or Lenti:mCh-SIIN/Cre containing sgRNAs targeting the tumor suppressors *Lkb1*, *Setd2*, or *Rb1*, in addition to an inert, non-targeting sgRNA. Mice were harvested 12 weeks after tumor initiation. **C** Representative H&E stained lung lobes from each experimental group. Scans were taken at 5x magnification. Scale bar is 1 mm. **D** Individual tumor area quantification for all experimental groups. Statistical significance was determined using one-way ANOVA testing with a Šidák correction. Error bars represent mean ± standard deviation. *n* = 10 mice per group for mCh/Cre-TS^WT^, mCh/Cre-*Lkb1*^*KO*^, and mCh/Cre-*Setd2*^*KO*^. *n* = 9 mice per group for mCh-SIIN/Cre-TS^WT^ and mCh/Cre-*Rb1*^*KO*^. *n* = 8 mice per group for mCh-SIIN/Cre-*Lkb1*^*KO*^, mCh-SIIN/Cre-*Setd2*^*KO*^, and mCh-SIIN/Cre-*Rb1*^*KO*^. Data pools together two independent experiments with similar results. **E** Representative IHC images of mCherry for each experimental group. Insets are 3x magnified and scale bar is 119um. **F** Qualitative assessment of mCherry expression in each experimental group. Each tumor image was divided into one of three categories based on its mCherry expression level: positive, partial, or negative. Significance across all groups was determined using a Fisher’s exact test. Number of individual tumors analyzed for each group is indicated on graph. Mouse number per group is the same as denoted in Fig. 3D. Data pools together two independent experiments with similar results. **G** Histogram plots showing H2Kb-SIINFEKL presentation measured by flow cytometry across each genotype specified. Plots are separated based on initiating vector and are normalized to mode. Dotted line separates positive and negative populations and value in the upper corner indicates the percentage of cells in that population. *n* = 4 mice per group for mCh/Cre-*Lbk1*^*KO*^, mCh/Cre-*Setd2*^*KO*^, mCh-SIIN/Cre-*Setd2*^*KO*^, and mCh-SIIN/Cre-*Rb1*^*KO*^. *n* = 3 mice per group for mCh/Cre-TS^WT^, mCh-SIIN/Cre-TS^WT^, mCh-SIIN/Cre-*Lkb1*^*KO*^, and mCh/Cre-*Rb1*^*KO*^.

To alter tumor suppressor genotype in the context of neoantigen expression, we next incorporated U6 promoter-driven single guide RNAs (sgRNAs) into the Lenti:mCh/Cre and Lenti:mCh-SIIN/Cre vectors (Lenti:mCh/Cre^sgRNA^ and Lenti:mCh-SIIN/Cre^sgRNA^; Fig. 3A). We initiated tumors in separate cohorts of *Kras*^*LSL-G12D/+*^*;Rosa26*^*LSL-Cas9::GFP/LSL-Cas9::GFP*^ (*K;Cas9*) mice with Lenti:mCh/Cre^sgRNA^ and Lenti:mCh-SIIN/Cre^sgRNA^ vectors that express functionally validated sgRNAs targeting one of three commonly inactivated tumor suppressor genes in lung adenocarcinoma (*Lkb1*, *Setd2*, or *Rb1*) or express an inert non-targeting sgRNA to generate control tumors driven solely by oncogenic KRAS (TS^WT^) (Fig. 3B, and Fig. S2). To best capture changes in tumor outgrowth associated with potent neoantigen expression, lungs were analyzed 12 weeks after tumor initiation. Unexpectedly, mCherry-SIINFEKL expression differentially influenced tumor growth across each genetic context (Fig. 3C, D). In TS^WT^ and *Lkb1*^*KO*^ tumors, mCherry-SIINFEKL expression led to a distinct, but non-significant, reduction in tumor area. Conversely, in the context of *Setd2*^*KO*^ and *Rb1*^*KO*^, mCherry-SIINFEKL expression had no impact on tumor growth. Quantification of the CRISPR-target inactivation showed a 40% reduction in the proportion of tumors deficient for the targeted gene’s protein in *Lkb1*^*KO*^ tumors expressing mCherry-SIINFEKL compared to *Lkb1*^*KO*^ tumors expressing mCherry alone (Fig. S2B). This effect was less prominent in the context of *Setd2*^*KO*^ (9% reduction) and non-significant in the context of *Rb1*^*KO*^ (6% reduction) (Fig. S2D and F).

In each genetic and immunogenic context, we assessed mCherry expression via IHC. Consistent with initial analyses (Fig. 1E, F), the majority of TS^WT^ tumors initiated by Lenti:mCh/Cre^sgRNA^ were mCherry^Positive^, as were *Lkb1*^*KO*^, *Setd2*^*KO*^, or *Rb1*^*KO*^ tumors initiated by Lenti:mCh/Cre^sgRNA^ vectors (Fig. 3E, F). Also consistent with initial data (Fig. 1E, F), a significantly lower percentage of TS^WT^ tumors initiated by Lenti:mCh-SIIN/Cre were mCherry^Positive^ (Fig. 3F). However, surprisingly, the propensity to select for mCherry-SIINFEKL silencing in cancer cells was distinct amongst *Lkb1*^*KO*^, *Setd2*^*KO*^, or *Rb1*^*KO*^ tumors. The vast majority of *Lkb1*^*KO*^ tumors lost mCherry expression, while *Setd2*^*KO*^ and *Rb1*^*KO*^ tumors remained similarly mCherry^Positive^ to those initiated by Lenti:mCh/Cre^sgRNA^. Additionally, we directly measured mCherry expression and MHC-I/SIINFEKL antigen presentation on cancer cells via flow cytometry on tumor cells (Fig. S3). In each group, MHC-I expression was uniformly high but SIINFEKL presentation and mCherry expression were consistent with that observed via IHC (Fig. 3E–G, Fig. S4). These data point to a selection of cancer cells with mCherry-SIINFEKL silencing rather than a general loss of MHC-I expression or lack of antigen presentation. Taken together, the data suggest that while mCherry-SIINFEKL expression promotes immunoediting across different tumor suppressor genotypes, *Lkb1*^*KO*^ most potently drives tumor immunosurveillance and editing upon mCherry-SIINFEKL expression, indicated both by the strong selection for tumors with inefficient *Lkb1* inactivation and the strong selection for tumors that have silenced neoantigen expression. In the context of *Setd2*^*KO*^ and *Rb1*^*KO*^, immunoediting and immune evasion likely occur through a different mechanism that does not necessitate neoantigen silencing.

Taken together, these data demonstrate that potent neoantigen expression induces different levels of immunosurveillance depending on the tumor genotype and most interestingly suggest that specific tumor suppressor gene inactivations that have the strongest effects promoting tumor growth may unexpectedly also have an outsized effect to promote early immunosurveillance.

### Tumor suppressor genotype differentially regulates immune responses to neoantigens

**Fig. 4:**
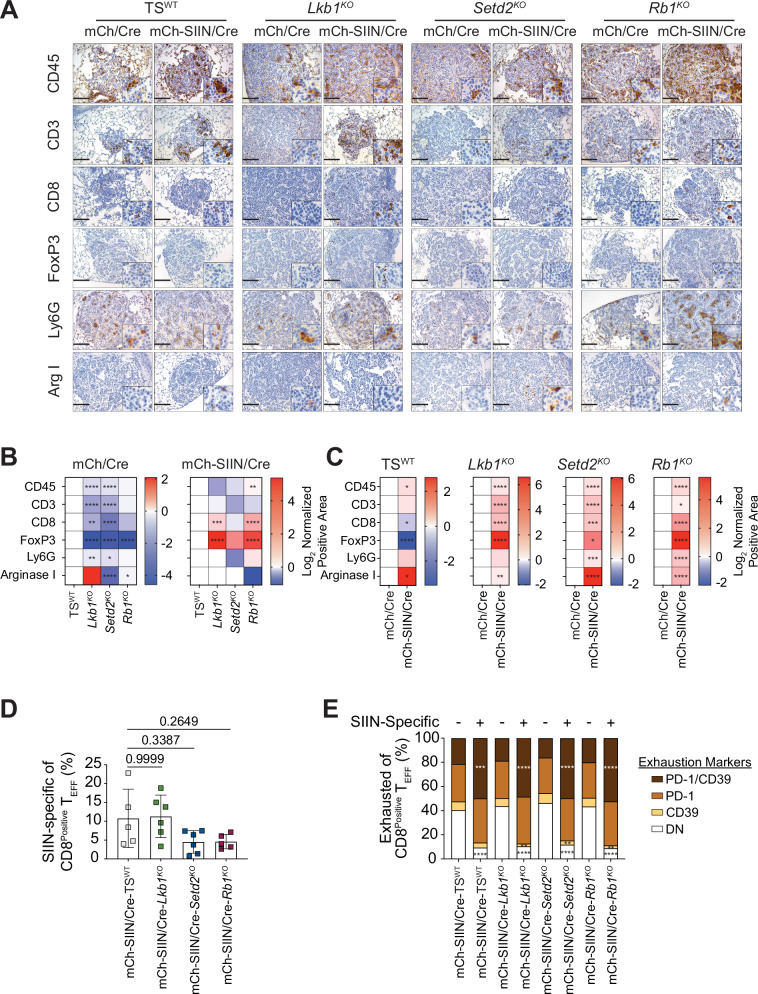
Tumor suppressor genotype differentially modulates immune responses in the context of potent antigen. **A** Representative 20x IHC images for the immune markers CD45, CD3, CD8, FoxP3, Ly6G, and Arginase I for all 8 experimental groups outlined in Fig. 3B. Insets are 3x magnified. Scale bar is 119um. IHC was performed on *n* = 4 mice per group except for mCh/Cre-Rb1^KO^, where *n* = 3. No independent repetitions of this staining were conducted. **B** Heat map depicting Log_2_ fold change in positive area for each IHC marker. Data are normalized to Lenti:mCh/Cre vector for each genotype. Asterisk indicates significance by Kruskal-Wallis test. Plot shows fold change in mean, but all individual values were used to calculate statistical significance. **C** Heat map depicting Log_2_ fold change in positive area for each IHC marker across all Lenti:mCh/Cre groups (left), or all Lenti:mCh-SIIN/Cre groups (right). Data are normalized to TS^WT^. Asterisk indicates significance by Kruskal-Wallis test. **D** Percentage of SIINFEKL-specific CD8^Positive^ T_EFF_ recruited to lungs of mice bearing mCh-SIIN/Cre tumors with either *Lkb1*, *Setd2*, or *Rb1* inactivation, as compared to TS^WT^ tumors. Statistical significance was determined a Kruskal-Wallis test. Error bars represent mean ± standard deviation. *n* = 6 for mCh-SIIN/Cre-*Lkb1*^*KO*^ and mCh-SIIN/Cre-*Setd2*^*KO*^, and *n* = 5 for mCh-SIIN/Cre-TS^WT^ and mCh-SIIN/Cre-*Rb1*^*KO*^. No independent repetition of this analysis was conducted. **E** Stacked plot representing exhaustion of SIINFEKL-specific and non-SIINFEKL-specific CD8^Positive^ T_EFF_ measured by PD-1 and CD39 positivity. Plot shows mean percentage per exhausted subtype, but all individual values were used to calculate statistical significance by nonparametric Student’s *t* tests. Asterisk indicates level of significance.

To gain insight into whether tumor suppressor genotype impacts the immune response to neoantigens, we first used IHC to quantify immune infiltration in lung tumors initiated with each Lenti:mCh/Cre^sgRNA^ or Lenti:mCh-SIIN/Cre ^sgRNA^ vector. In tumors initiated by Lenti:mCh/Cre^sgRNA^ vectors, tumor suppressor gene inactivation promoted immunosuppression, with a strong (*Lkb1*^*KO*^ and *Setd2*^*KO*^) or mild (*Rb1*^*KO*^) reduction in general CD45^Positive^ immune infiltration as well as decreases in CD8^Positive^ T cells, FoxP3^Positive^ T_regs_, Ly6G^Positive^ neutrophils, and Arginase I^Positive^ myeloid derived suppressor cells (MDSCs) (Fig. 4A, B, and Fig. S5). Across every tumor suppressor genotype, CD45^Positive^, CD3^Positive^ and CD8^Positive^ immune cells were more prevalent in tumors initiated by Lenti:mCh-SIIN/Cre^sgRNA^ compared to tumors initiated by the corresponding Lenti:mCh/Cre^sgRNA^ vectors (Fig. 4A, C and Fig. S5A–C). This suggests that mCherry-SIINFEKL expression elicits a more potent anti-tumor T cell response than mCherry alone, regardless of tumor suppressor genotype. However, infiltration of immunosuppressive cell types in tumors with SIINFEKL expression varied dramatically based on tumor suppressor genotype. Compared to TS^WT^ tumors initiated by Lenti:mCh-SIIN/Cre^sgRNA^, FoxP3^Positive^ regulatory T cell (T_reg_) infiltration was significantly higher in *Lkb1*^*KO*^ and *Rb1*^*KO*^ tumors but not in *Setd2*^*KO*^ tumors (Fig. 4A, B, and Fig. S5D). Additionally, comparing tumors initiated by Lenti:mCh/Cre^sgRNA^ and Lenti:mCh-SIIN/Cre^sgRNA^ revealed that both *Setd2*^*KO*^ and *Rb1*^*KO*^ tumors had high Arginase I^Positive^ MDSC and Ly6G^Positive^ neutrophil infiltration in the context of mCherry-SIINFEKL expression, which was not observed in TS^WT^ or *Lkb1*^*KO*^ tumors (Fig. 4C). While prior studies have linked *Lkb1* inactivation to increased neutrophil recruitment, these findings align with our previous work where LKB1 restoration in established tumors had no effects of neutrophil abundance^11,26^.

We next assessed T cell phenotypes by flow cytometry on tumor-bearing lungs. While there were no significant differences in the frequency of general CD3^Positive^, CD8^Positive^, or CD4^Positive^ T cells in total lung tissue across each genotype or immunogenic condition (Fig. S6, S7), both CD8^Positive^ central memory and CD4^Positive^ central memory T cells (T_CM_) were significantly reduced specifically in the context of *Lkb1*^*KO*^ tumor-bearing lungs, but not in *Setd2*^*KO*^ or *Rb1*^*KO*^ tumor-bearing lungs (Fig. S8A–D). Conversely, the percentage of SIINFEKL-specific T_EFF_ cells in the lungs of *Setd2*^*KO*^ and *Rb1*^*KO*^ tumor-bearing mice was two-fold lower than in lungs with TS^WT^ and *Lkb1*^*KO*^ tumors despite a similar frequency of total (SIIN-specific and non-SIIN-specific) T_EFF_ and naïve T (T_N_) cell populations across each genotype and immunogenic condition (Fig. 4D, Fig. S8A and B). Moreover, SIINFEKL-specific T_EFF_ cells were significantly more exhausted compared to non-SIINFEKL-specific T_EFF_ cells, as evidenced by the increased frequency of PD-1/CD39 double-positive cells (Fig. 4E). This phenotype of SIINFEKL-specific T_EFF_ cell exhaustion occurred regardless of tumor suppressor genotype. Together, these data suggest that mCherry-SIINFEKL expression successfully generates anti-tumor immune responses across each tumor suppressor genotype tested, but that distinct genotype-specific mechanisms allow subsequent immune evasion.

### Parallel analysis of tumor genotype across multiple immunogenic contexts via tumor barcoding

**Fig. 5:**
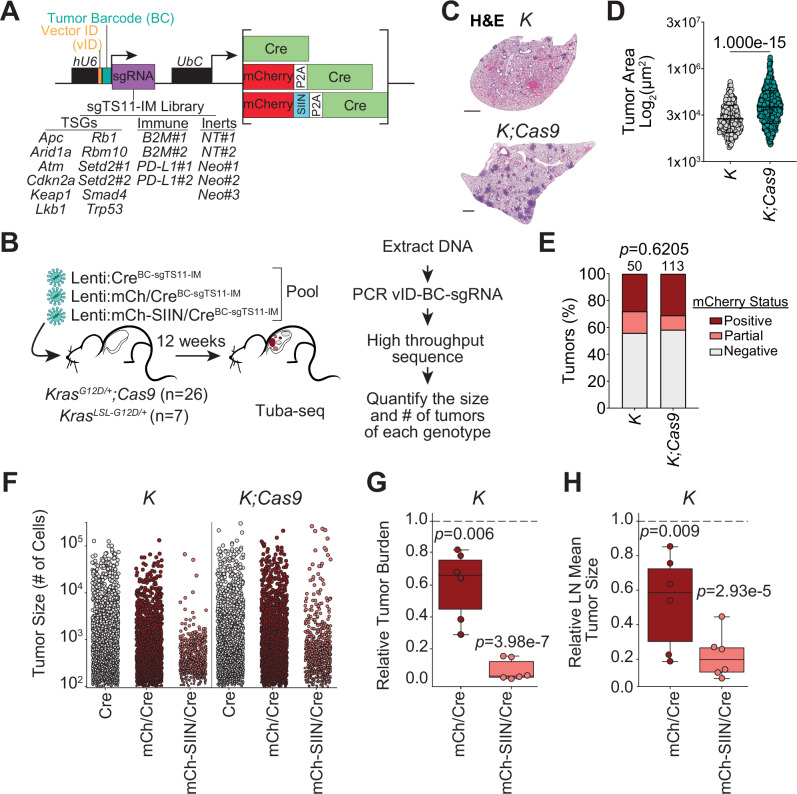
Multiplexed somatic genome editing and tumor barcoding uncover the impact of tumor suppressor gene inactivation on immunosurveillance. **A** Design of immunogenic series of lenti-vectors used for Tuba-seq screen. These lenti-vectors include a 4nt vector ID, a 10nt tumor barcode, as well as a library of sgRNAs targeting frequently mutated tumor suppressor genes in lung adenocarcinoma, immunomodulatory genes, as well as inert, non-targeting sgRNAs. **B Left:** Experimental design. Tumors were initiated in either *K* (*n* = 7) or *K;Cas9* (*n* = 26) mice using a pool of Lenti:Cre, Lenti:mCh/Cre, and Lenti:mCh-SIIN/Cre each containing the sgRNA library depicted in Fig. 5A. **Right:** Flow chart summarizing the Tuba-seq pipeline from sacrificing mice to analyzing sequencing results. **C** Scans of a representative H&E stained lung lobe from a *K* or *KC* mouse at 5x magnification. Scale bar is 1 mm. **D** Quantification of individual tumor area in *K* vs *K;Cas9* mice. Statistical significance was determined using an unpaired, two-tailed Student’s *t*-test. Error bars represent mean ± standard deviation. For *K* mice, *n* = 258 tumors from *n* = 3 mice. For *K;Cas9* mice, *n* = 628 tumors from *n* = 5 mice. **E** Qualitative assessment of mCherry expression in *K* (*n* = 3) and *K;Cas9* (*n* = 5) mice based on IHC from Supplementary Fig. 5D. Number of individual tumors analyzed for each group is indicated on graph. Significance was determined using a chi-squared test. **F** Jitter plot of all tumor size for barcoded clonal tumors across each immunogenic context (Cre^BC-sgTS11-IM^, mCh/Cre^BC-sgTS11-IM^, and mCh-SIIN/Cre^BC-sgTS11-IM^) in *K* mice and all TS^WT^ tumors in *K;Cas9* mice. **G**, **H** Tumor burden (**H**) or LN mean tumor size (**I**) of mCh/Cre^BC-sgTS11-IM^ and mCh-SIIN/Cre^BC-sgTS11-IM^ tumors relative to Cre^BC-sgTS11-IM^ tumors across *n* = 6 *K* mice. Box plots show the median (center line), interquartile range (box; 25th–75th percentiles), and whiskers extending to 1.5x the interquartile range. Individual data points from each mouse are overlaid as dots. One-sample t-tests were used to determine if the estimates were significantly different from 1.

To assess the impact of multiple tumor suppressor genotypes in parallel across different immunogenic contexts in a highly quantitative manner, we generated sgRNA-expressing versions of Lenti:Cre, Lenti:mCh/Cre, and Lenti:mCh-SIIN/Cre for tumor barcoding coupled with high-throughput sequencing (Tuba-seq, Fig. 5A)^23,27–31^. Tuba-seq is based on lentiviral-mediated integration of barcode sequences into the genome of the initiating cell of each lung tumor clone. To enable pooling of different immunogenic vectors and the generation of different tumor suppressor genotypes, we inserted a 4-nucleotide vector ID (vID; which is unique to each immunogenic vector) and a diverse tumor barcode (BC; which is unique to each clonal tumor) within the 5′ region of the U6 promoter directly preceding the sgRNA^23,27–31^. Thus, amplification and high-throughput sequencing of the vID-BC-sgRNA region from bulk tumor-bearing lung DNA can quantify the size of each tumor (BC reads), as well as the genotype (sgRNA) and immunogenic context (vID).

We expanded an sgRNA library that we have extensively validated which targets 11 tumor suppressor genes that are commonly inactivated in human lung adenocarcinoma (TS11, Fig. 5A)^23,27–31^. Additionally, we included sgRNAs targeting the immunomodulatory (IM) genes beta-2-microglobulin (*B2M*) and *CD274* (*PD-L1*) as controls that should have opposite effects on tumor outgrowth in a highly immunogenic context, as well as inert/non-targeting (sgInert) control sgRNAs (21 total sgRNAs in each vector; Fig. 5A). A pool of all barcoded sgRNAs was cloned into each vector to generate Lenti:Cre^BC-sgTS11-IM^, Lenti:mCh/Cre^BC-sgTS11-IM^, and Lenti:mCh-SIIN/Cre^BC-sgTS11-IM^ (Fig. 5A, Fig. S9A, Fig. S10, and Methods). We transduced *K* (*n* = 7) and *K;Cas9* (*n* = 26) mice with a single pool containing the Lenti:Cre^BC-sgTS11-IM^, Lenti:mCh/Cre^BC-sgTS11-IM^, and Lenti:mCh-SIIN/Cre^BC-sgTS11-IM^ vectors (63 vectors total) and analyzed lungs 12 weeks after tumor initiation (Fig. 5B and C, Fig. S9B). As expected, due to inactivation of potent tumor suppressors, tumor area in *K;Cas9* mice was greater than in *K* mice (Fig. 5C and D, Fig. S9B). Tumors in *K* mice were all adenomas, whereas *K;Cas9* mice had only very rare (~1%) adenocarcinomas (6 out of 628 tumors). A subset of tumors was mCherry^Positive^ consistent with mCherry expression in a fraction of tumors initiated by Lenti:mCh/Cre^BC-sgTS11-IM^ and Lenti:mCh-SIIN/Cre^BC-sgTS11-IM^ (Fig. 5E, Fig. S9B and C). The vID-BC-sgRNA region was PCR amplified from genomic DNA isolated from tumor-bearing lungs, followed by high-throughput sequencing and Tuba-seq analysis (Fig. 5B, Fig. S11A and B, and Methods).

To establish the baseline effects of expressing mild or strong neoantigens on immunosurveillance in TS^WT^ tumors, we quantified the relative impact of mCherry or mCherry-SIINFEKL expression on tumor burden and tumor size compared to poorly immunogenic tumors initiated by Lenti:Cre^BC-sgTS11-IM^. Tumors initiated with either Lenti:mCh/Cre^BC-sgTS11-IM^ or Lenti:mCh-SIIN/Cre^BC-sgTS11-IM^ had a significantly lower tumor burden and smaller log normal mean tumor size (Fig. 5F–H, Fig. S11C–F). Moreover, Lenti:mCh-SIIN/Cre^BC-sgTS11-IM^ reduced tumor burden and tumor size more than Lenti:mCh/Cre^BC-sgTS11-IM^. Taken together, these findings provide additional quantitative support for the increasing tumor immunogenicity of each lentiviral-based vector and establish Tuba-seq as a method to quantitatively and precisely assess the impact of neoantigen expression on immunosurveillance in different immunogenic contexts.

### CRISPR-mediated inactivation of mediators of immune surveillance predictably impacts tumor size

**Fig. 6:**
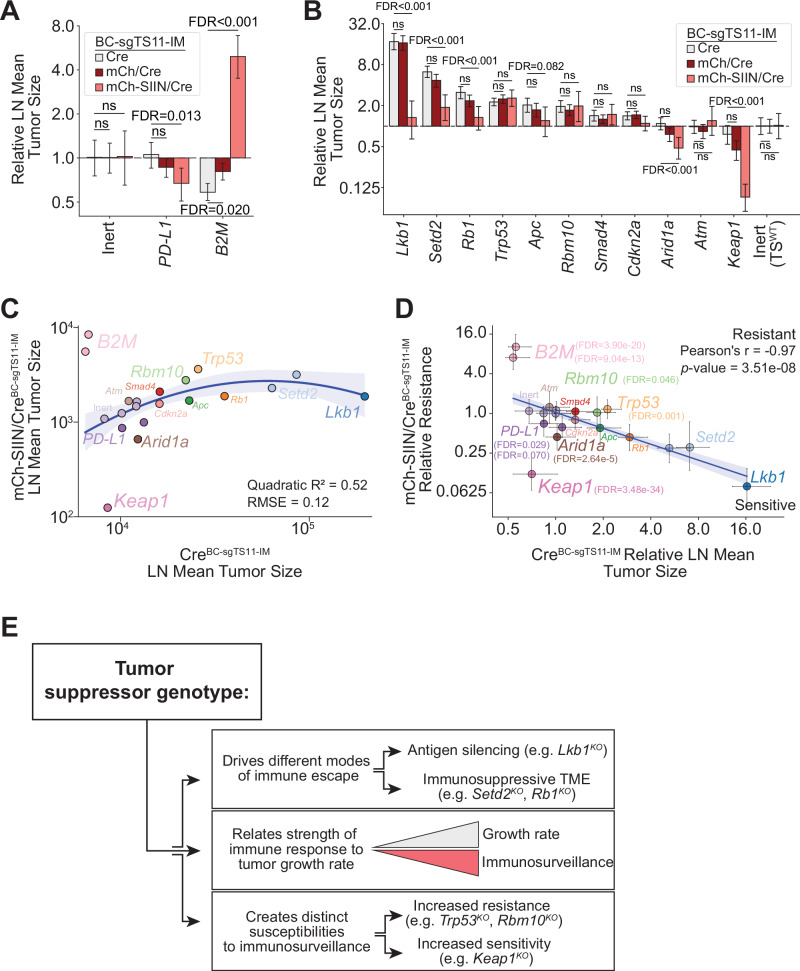
Highly proliferative tumor suppressor genotypes exhibit lower resistance to immunosurveillance. **A** Quantification of relative LN mean tumor size for the TS^WT,^ *PD-L1*^*KO*^, and *B2M*^*KO*^ genotypes initiated with Lenti:Cre^BC-sgTS11-IM^, Lenti:mCh/Cre^BC-sgTS11-IM^, or Lenti:mCh-SIIN/Cre^BC-sgTS11-IM^ across *n* = 24 *K;Cas9* mice. Data are shown as medians with 95% confidence intervals from 10,000 bootstrap iterations. Empirical two-sided *p*values were computed from bootstrap comparisons between groups and adjusted for multiple testing using FDR correction. **B** Quantification of relative LN mean tumor size for the 11 tumor suppressor genotypes initiated with Lenti:Cre^BC-sgTS11-IM^, Lenti:mCh/Cre^BC-sgTS11-IM^, or Lenti:mCh-SIIN/Cre^BC-sgTS11-IM^ in *K;Cas9* mice. Data are shown as medians with 95% confidence intervals from 10,000 bootstrap iterations. Empirical two-sided *p-*values were computed from bootstrap comparisons between groups and adjusted for multiple testing using FDR correction. **C** Quadratic fit of the LN mean tumor size between Lenti:Cre^BC-sgTS11-IM^ and Lenti:mCh-SIIN/Cre^BC-sgTS11-IM^ across different sgRNA in the BC-sgTS11-IM pool. **D** The relationship between the Relative Resistance metric of mCh-SIIN/Cre^BC-sgTS11-IM^ tumors and the relative LN mean tumor size of Cre^BC-sgTS11-IM^ tumors across different sgRNAs in the BC-sgTS11-IM pool. *p*-values indicate the significance of each gene’s deviation from the global LN mean tumor size versus resistance trend. For each gene, a z-score was computed from the vertical residual relative to a linear regression fit between LN mean tumor size and resistance. Two-sided *p*-values were calculated assuming normally distributed residuals and adjusted for multiple testing using FDR correction. Data are shown as medians with 95% confidence intervals from 10,000 bootstrap iterations. **E** One-at-a-time gene knockout approaches reveal that tumor suppressor genotype is an important mediator of the immune response mounted against the highly immunogenic SIINFEKL neoantigen. In the context of *Setd2*^*KO*^ and *Rb1* ^*KO*^, tumor suppressor gene inactivation sufficiently promotes immunoevasion such that tumors can grow despite sustained neoantigen expression. However, *Lkb1* ^*KO*^ tumors require the selection of neoplastic cells which have silenced neoantigen expression and surface presentation in order to sufficiently evade immune detection. Using a multiplexed high throughput in vivo screen combining CRISPR/Cas9-mediated genome editing, neoantigen expression, and tumor barcoding, we simultaneously investigated the impact of 11 tumor suppressor gene inactivations on immunosurveillance in three distinct and increasingly immunogenic contexts. Surprisingly, tumor suppressor genotypes that most strongly promote tumor outgrowth in poorly immunogenic contexts are most sensitive to immunosurveillance in highly immunogenic contexts. However, specific tumor suppressor gene inactivations were exceptions where *Trp53*^*KO*^ and *Rbm10*^*KO*^ promoted resistance to immunosurveillance and *Keap1* ^*KO*^ promoting exceptional sensitivity to immunosurveillance.

*B2M* is an essential component of the major histocompatibility complex class I (MHC-I). Loss of MHC-I through *B2M* inactivation drives resistance to immune checkpoint therapy and is predicted to facilitate resistance to immunoediting in neoantigen-expressing tumors^7–9^. However, MHC-I expression is a determinant of ‘*self’* and inhibits innate immune responses that cull MHC-I negative cells^32,33^. As such, *B2M*^*KO*^ may have multifaceted effects on tumor number and tumor size in vivo. Indeed, relative to Inert (TS^WT^) tumors, *B2M*^*KO*^ greatly reduced the size of poorly immunogenic Cre tumors (Fig. 6A, Fig. S12, S13A and B). However, *B2M*^*KO*^ only slightly reduced the size of the mildly immunogenic mCherry tumors and greatly increased the size of the highly immunogenic mCherry-SIINFEKL tumors. Furthermore, *B2M*^*KO*^ increased tumor initiation across all immunogenic conditions (Fig. S13C). These data suggest that while loss of ‘*self’* is generally detrimental to tumor growth, the benefit of no longer presenting mildly (mCherry) or highly (mCherry-SIINFEKL) immunogenic neoantigens outweighs this detriment. In contrast, inactivation of *PD-L1* should sensitize tumors to immunoediting. *PD-L1*^*KO*^ had no effect on the size of Cre tumors, and only a mildly negative effect on mCherry tumors. However, *PD-L1*^*KO*^ greatly reduced the size and number of highly immunogenic mCherry-SIIN tumors, indicative of enhanced immunoediting (Fig. 6A, Fig. S13, A to C). These results further underscore the graded immunogenicity of Lenti:Cre^BC-sgTS11-IM^, Lenti:mCh/Cre^BC-sgTS11-IM^, and Lenti:mCh-SIIN/Cre^BC-sgTS11-IM^ within autochthonous tumors and demonstrate our ability to discern complex effects of tumor genotype on the susceptibility to immune surveillance.

### Tumor suppressor gene inactivation has multifaceted effects on immune surveillance in immunogenic tumors

To determine the impact of tumor suppressor gene inactivation on immunosurveillance, we quantified metrics of tumorigenesis for each tumor genotype relative to TS^WT^ tumors in each immunogenic condition (Fig. S12, Methods). In tumors initiated by Lenti:Cre^BC-sgTS11-IM^, tumor suppressor gene inactivation generated the expected tumor growth-promoting effects (Fig. 6B, Fig. S13D–F)^23,29^. In the context of each tumor suppressor gene inactivation, expression of neoantigens generally had a growth suppressing effect that correlated with the potency of each neoantigenic context (Fig. 6B). However, *Trp53*^*KO*^*, Rbm10*^*KO*^, and to a lesser extent *Smad4*^*KO*^ tumors initiated by Lenti:mCh-SIIN/Cre^BC-sgTS11-IM^ were similar in relative tumor size to those initiated by Lenti:Cre^BC-sgTS11-IM^, suggesting that inactivation of these genes promotes resistance to tumor immunosurveillance. In contrast, *Keap1*^*KO*^ and *Arid1a*^*KO*^ tumors initiated by Lenti:mCh-SIIN/Cre^BC-sgTS11-IM^ were significantly smaller in relative tumor size to TS^WT^ tumors, suggesting that inactivation of these genes further promotes tumor immunosurveillance (Fig. 6B).

The differential effects of tumor suppressor gene inactivation across immunogenic contexts prompted us to determine if there was a relationship between potency of tumor suppression in poorly immunogenic contexts and the sensitivity to immune surveillance in the highly immunogenic context. To determine whether specific genetic contexts strongly affected tumor immunosurveillance, we directly compared the log-normal mean tumor size in tumors initiated by Lenti:Cre^BC-TS11-IM^ to that of tumors initiated by Lenti:mCh-SIIN/Cre^BC-TS11-IM^. Unexpectedly, tumor genotypes that more strongly promoted tumor growth in the poorly immunogenic context were significantly more susceptible to immune-mediated clearance in the highly immunogenic context (Fig. 6C, Fig. S14A). This trend was not observed when comparing tumors initiated by Lenti:Cre^BC-TS11-IM^ to tumors initiated by Lenti:mCh/Cre^BC-TS11-IM^, and as such, this proliferation-dependent sensitivity to immunosurveillance appears reliant on a highly immunogenic stimulus (Fig. S14B).

To directly assess the sensitivity of tumors to immune surveillance across genotypes, we calculated a metric of relative resistance (R) by normalizing the relative LN mean tumor size in the highly immunogenic context to the relative LN mean tumor size in the poorly immunogenic context. Instead of a constant R across genotypes, there was a clear negative linear correlation between resistance and tumor growth in the poorly immunogenic context, which did not occur in the context of tumors initiated by Lenti:mCh/Cre^BC-sgTS11-IM^, suggesting that more proliferative tumors are more sensitive to immune surveillance in highly immunogenic conditions (Fig. 6D and Fig. S14C–E). Through these analyses, we identified *Keap1*^*KO*^, *Arid1a*^*KO*^*, Trp53*^*KO*^, and *Rbm10*^*KO*^ as outliers (Fig. 6D). That *Keap1*^*KO*^ and *Arid1a*^*KO*^ tumors are positioned below the trend-line supports their increased sensitivity to immunosurveillance, while *Trp53*^*KO*^ and *Rbm10*^*KO*^ positioning above the trend-line suggests that they drive resistance to immunosurveillance.

All together, these findings uncovered both a general negative correlation between resistance to immunosurveillance and proliferation rate and highlighted specific tumor genotypes that further sculpt tumor immunosurveillance during early tumor development (Fig. 6E).

## Discussion

To create personalized immunotherapy approaches for cancer, it is critical to understand the impact of tumor genotype on the natural mechanics of immune surveillance. In this study, we describe two complementary approaches for assessing the impact of tumor suppressor genotype on immunosurveillance in autochthonous lung cancer models. Using individual gene knockout models, we investigated the effect of *Lkb1*, *Setd2*, and *Rb1* inactivation on immunoediting in KRAS-driven tumors under discrete immunogenic conditions. These single-gene knockout studies provided important mechanistic insight by allowing evaluation of neoantigen expression and presentation, as well as immune cell recruitment, activation, and exhaustion. Our data show that *Lkb1*^*KO*^, *Setd2*^*KO*^, and *Rb1*^*KO*^ tumors exhibited different levels of immunosurveillance in response to a highly immunogenic stimulus, and each escaped immune pressures through distinct regulation of neoantigen expression, T cell engagement, and recruitment of immunosuppressive cell subtypes (Fig. 6E). However, the breadth of analyses with single gene knockout models is limited by the need for large numbers of mice to test multiple immunogenic contexts. To overcome this, we integrated vectors that express defined antigens with multiplex in vivo tumor barcoding that is coupled with high-throughput sequencing (Tuba-seq) and quantitatively assessed 14 tumor genotypes across three immunogenic conditions in parallel (Fig. 5, A and B). While the pooled Tuba-seq approach mimics the genetic heterogeneity seen in human cancers, it also allows for potential inter-tumor crosstalk or field effects, in which one tumor clone’s genotype may influence the immunosurveillance of neighboring clones. However, such field effects would only serve to limit the ability to detect cell autonomous gene-specific effects in the heterogeneous pool, suggesting that our detected Tuba-Seq *hits* must have a relatively outsized effect to modulate immune surveillance. Regardless, while pooled approaches are essential for capturing broad quantitative patterns, they are best paired with one-at-a-time gene knockout studies to fully resolve how tumor genotypes regulate immune responses. Together, these complementary strategies establish a toolkit to generate conditional tumor models with predictable immunogenicity across genotypes and to identify both positive and negative regulators of immunosurveillance.

Our data indicate that across TS genotypes, the ability for a tumor to expand in poorly immunogenic conditions predicts the extent of immunosurveillance in highly immunogenic conditions. While it is possible that highly immunogenic conditions impose an upper limit on tumor size, our data demonstrate that there is an inverse relationship between TS genotypes that strongly promote tumor growth in poorly immunogenic conditions and the extent of tumor immunosurveillance. In fact, the relationship between the degree of immunosurveillance due to expression of strong neoantigens and the tumor expansion rate due to tumor genotype seems to strengthen in a non-linear manner (Fig. 6C). This contrasts with prior findings from transplantable cancer cell line models reporting a general association between tumor suppressor loss and immune evasion^17^. This apparent discrepancy is most likely driven by the many differences between subcutaneous transplantation of in vitro manipulated cancer cell lines and the in vivo transformation of normal epithelial cells growing in their natural setting. These very different scenarios likely examine distinct aspects of immune surveillance that deserve additional attention. We speculate that our approach may afford insight into the role of immune pressure during the early phases of cancer formation and thus reveal important features of cancer etiology that may inform strategies for cancer interception.

In contrast to the broad effects related to tumor growth potential, we found that specific tumor suppressor gene inactivations limit immune surveillance, while others surprisingly exacerbate immune surveillance. *Trp53* or *Rbm10*-deficient tumors are relatively resistant to immune surveillance. Consistently, multiple studies have associated *Trp53* inactivation with immune evasion, and p53 restoration enhances anti-tumor immunity^34–39^. However, more work is needed to understand how *Rbm10* inactivation promotes resistance to immunosurveillance in the context of potent neoantigens, as fully established RBM10-deficient tumors have been shown to stimulate anti-tumor pathways^16,40,41^. In contrast, *Arid1a* or *Keap1*-deficient tumors are relatively sensitive to immunosurveillance in the context of strong neoantigen expression. *Arid1a* inactivation is associated with anti-tumor immunity and greater progression-free survival in patients on anti-PD-1 therapy across multiple cancer types, including non-small cell lung cancer^42,43^. *Arid1a* inactivation has also been shown to promote anti-tumor immunity via increased R-loop-derived cytosolic single-stranded DNA that activates inflammatory signaling pathways^44^.

Interestingly, *Keap1*-deficient tumors, which were the most sensitive to immunosurveillance, are more commonly associated with immunosuppression and resistance to anti-PD-1/PD-L1 therapies^19,45,46^. This seemingly discrepant result may point to *Keap1* inactivation as an early driver of immunosurveillance, which in turn selects for tumor escape mechanisms that drive immune evasion and subsequent resistance to PD-1/PD-L1 therapy. This parallels our findings with *Lkb1* inactivation, where, despite its well-described immunosuppressive role, our data show that *Lkb1* inactivation induces early immunosurveillance (Fig. 3F and G, Fig. S2A and B, Fig. S4A and B)^10,11,13,14^. Notably, while *Lkb1* and/or *Keap1* inactivation is associated with resistance to PD-1/PD-L1 blockade, these genotypes increased sensitivity to dual checkpoint therapy with CTLA-4 in analogous preclinical KRAS-driven lung cancer models and patients^47^. This suggests that early induction of immunosurveillance by these genotypes may lead to immune-evasive mechanisms that only dual blockade therapy can overcome. Future experiments testing several sgRNAs per gene across both early and late-stage tumor development may provide further support for our observations and additionally uncover how these genotypes shape tumor-immune dynamics over time.

Our study provides evidence that the driver mutations responsible for malignant transformation shape a tumor’s susceptibility to immunoediting and the mechanisms by which immunoediting occurs. These insights may inform patient selection and uncover therapeutic vulnerabilities that increase efficacy of immune checkpoint therapies^12,13,15,18,43,45,47^.

## Methods

### Ethics statement

The research conducted in this study was conducted ethically and complies with all relevant guidelines and regulations. Animal studies were performed under compliance with Institutional Animal Care and Use Committee (IACUC) at University of Pennsylvania (#804774). Tumors did not exceed the combined diameter of 3.0 cm permitted by this IACUC protocol.

### Animal Studies

All work was performed under compliance with Institutional Animal Care and Use Committee at the University of Pennsylvania (#804774). *Kras*^*LSL-G12D*^ (*K*) mice (Jax stock number 008179), *Rosa26*^*LSL-Cas9::EGFP*^ (*Cas9*) mice (Jax stock number 026175), and *Rag1*^*-/-*^ mice (Jax stock number 002216) were maintained on a B6J background. Tumors were transduced via endotracheal inhalation of a lentiviral-based vectors that expresses Cre-recombinase by 20 weeks of age^48^. To initially determine how each vector modulates immunoediting, *K* mice were transduced with 1×10^5^ plaque forming units (PFU) per mouse of Lenti:Cre, Lenti:mCh/Cre, or Lenti:mCh-SIIN/Cre. To test how specific tumor suppressor genes impact immunoediting, *K;Cas9* mice were transduced with 6×10^4^ PFU of Lenti:mCh/Cre or Lenti:mCh-SIIN/Cre containing an inert, non-targeting sgRNA or a sgRNA targeting *Lkb1*, *Setd2*, or *Rb1*. For the Tuba-seq screen, each *K;Cas9* mouse was transduced with a combination of Lenti:Cre^BC-sgTS11-IM^, Lenti:mCh/Cre^BC-sgTS11-IM^, and Lenti:mCh-SIIN/Cre^BC-sgTS11-IM^ that contained the barcoded sgRNA pool (6×10^4^ PFU per virus, 1.8×10^5^ PFU total per mouse). *K* mice in this screen were transduced with the same three viruses but using 1×10^5^ PFU per virus per mouse (3×10^5^ PFU total per mouse). All animal studies were randomized in ‘control’ or ‘treated’ groups. However, all animals housed within the same cage were generally placed within the same treatment group. Animal sex was randomized at the time of treatment group assignment. Studies were balanced by sex, and no sex-specific effects were observed. Mice were housed in a vivarium with regulated light-dark cycles (12 h), ambient temperatures (20–24 °C), and relative humidity (45–65%). Fluorescent images of GFP-or mCherry-positive tumors in mouse lungs were captured using a Leica M80 compact stereomicroscope.

### Immunohistochemistry

Lungs were inflated using a 10% neutral-buffered formalin and then placed into the formalin solution overnight at room temperature before dehydration in a graded alcohol series. Paraffin-embedded and H&E sections were produced by the Penn Molecular Pathology and Imaging core. Immunohistochemistry was performed after a citrate-based antigen retrieval using the following antibodies: CD45 (1:100, Biolegend, 103102), CD3 (1:700, Abcam, ab5690), mCherry (1:500, Novus Biologicals, NBP2-25156SS), LKB1 (1:250, Cell Signaling, 13031S), RB (1:50, Abcam, ab181616), H3K36me3 (1:2000, Abcam, ab9050), CD8 (1:500, Cell Signaling, 98941S, clone D4W2Z), FoxP3 (1:500, Cell Signaling, 12653S, clone D6O8R), Ly6G (1:500, Biolegend, 127601, clone IA8), and Arginase I (1:1000, Thermo Scientific, PA5-29645). Primary antibodies were incubated overnight at 4 °C. After primary antibody staining, an ABC reagent for rabbit (Vector Laboratories, PK-4001) or rat (Vector Laboratories, PK-6104) and ImmPACT DAB (Vector laboratories, SK-4105) were used per manufacturer’s instructions. Sections were counterstained with Hematoxylin (1:10, Sigma-Aldrich, GHS316-500ML). All micrographs were captured on a Leica DMI6000B inverted light and fluorescence microscope. IHC staining was quantified on ImageJ using Mean Gray Value as previously described^49^. All mean gray values were then divided by 255 to convert the value to percent positive area.

Individual tumor area and tumor burden were analyzed using ImageJ. Each tumor was individually circled on ImageJ using the freehand selection tool. The area was measured for each tumor in pixels on ImageJ. Tumor burden was calculated by dividing the sum of tumor area within a lung divided by the total lung area. GraphPad Prism 10 was then used to graph tumor metrics and conduct statistical analyses.

Qualitative assessments of mCherry were done using 20x IHC images. Each image was assessed and banked into one of three groups: negative, partial, or positive depending on its mCherry expression status. The number of tumors in each category was summed and divided by the total number of tumors assessed to determine percentages for each group. GraphPad Prism 10 was then used to graph tumor metrics and conduct statistical analyses. Chi-squared or Fisher’s Exact analyses were used to determine statistical significance.

### Plasmids

All vectors used in these experiments were derived from LentiCRISPRv2Cre (Addgene plasmid #82415). The Ef-1α promoter and Cas9 gene were removed from the plasmid by cutting with EcoRI and BamHI restriction enzymes. All restriction digests were run according to protocols found using NEBcloner (New England Biolabs). Then, a Gibson assembly reaction was used to insert the UbC promoter alone or to insert UbC-mCherry to generate Lenti:Cre or Lenti:mCh/Cre. Lenti:mCh-SIIN/Cre was then generated by linearizing Lenti:mCh/Cre plasmid with XhoI and performing a second Gibson reaction to insert the SIINFEKL DNA sequence directly after mCherry. Each new plasmid was transformed and individual colonies were sequenced by Sanger sequencing to confirm successful cloning.

For sgRNA expression from the U6 promoter, it is necessary to maintain a 23 nt gap between the promoter TATA box and the first transcribed G nucleotide. To account for the vID and BC sequence, the hU6 promoter, spacer, and part of the chimeric sgRNA backbone were removed using KpnI and AfeI. A modified insert containing a shortened spacer and a vector ID was inserted into each vector by Gibson assembly. This insert also restored the hU6 and chimeric sgRNA backbone sequences. vID insertion was confirmed by Sanger sequencing transformed colonies.

### Tuba-seq Plasmid Library Generation and Validation and Individual sgRNA Insertion

sgRNA sequences for Tuba-seq were previously validated and published^23^. Additional sgRNAs targeting ß−2M and PD-L1 were generated using the Sanjana Lab CRISPR Cas9 library design tool (http://guides.sanjanalab.org). ß−2M sgRNA #1: 5′-GTATGTATCAGTCTCAGTGG-3′, ß−2M sgRNA #2: 5′-AGACAAGCACCAGAAAGACC-3′. PD-L1 sgRNA #1: 5′-GTTTACTATCACGGCTCCAA-3′, PD-L1 sgRNA #2:−5′ GGATGATCAGCTCCGCTGTG-3′. Each sgRNA oligo contained the following sequence: 5′- GGAGACCTGCGTCTCACACCHHHHHHHHHHG-sgRNA sequence-GTTTAGAGACGGCTCCGCGC- 3′. The 10nt tumor barcodes only consisted of A, C, or T nucleotides to minimize the chance of early initiation of transcription at an upstream G nucleotide. Oligos were ordered separately and then pooled together at 1 ng/uL for PCR amplification using NEB Fusion HS Flex Enzyme and Buffer (New England Biolabs, M0535S),10 mM dNTPs, and primers (Forward: 5′ GGAGACCTGCGTCTCACACC 3′, Reverse: 5′ GCGCGGAGCCGTCTCTAAAC 3′). PCR conditions were as follows: 98 °C for 2 min, 98 °C for 8 s*, 65 °C for 12 s*, 72 °C for 10 s*, 72 °C for 5 min, then 4 °C indefinitely. The starred conditions were repeated for 20 cycles. PCR product was run on a 2% agarose gel and gel purified using the QIAquick Gel Extraction Kit (Qiagen, 28704). Simultaneously, Lenti:Cre, Lenti:mCh/Cre, or Lenti:mCh-SIIN/Cre plasmids were digested with BsmBI-v2 (New England Biolabs, R0739S). The digest was run on a 1% agarose gel and the digested vector was excised and purified from the gel using the QIAquick Gel Extraction Kit.

Both the sgRNA oligo and the digested vector were further purified using an ethanol precipitation. Then, a golden gate reaction was performed by mixing 500 ng of digested vector, 100 ng sgRNA oligo insert, 10 units Esp3I (Thermo Fisher, ER0452), 3000 units T7 ligase (Enzymatics, L6020L), 1 mM DTT, and 1 mM ATP. Samples were cycled between 37 °C and 20 °C every 5 minutes overnight for 100 cycles. The new plasmid libraries were purified using the MinElute PCR Purification Kit (Qiagen, 28004) and eluted in 10 uL of water.

Plasmid libraries were transformed using MegaX DH10B T1^R^ Electrocomp™ Cells (Invitrogen, C640003). 2 uL of the golden gate product was mixed with 20 uL of electrocompetent cells and then electroporated in a 1 mm cuvette using the following pulse conditions: 25 µF capacitance, 200 Ω resistance, and 2000 V voltage. Transformed cells underwent 1 h of recovery and then were transferred to a flask containing 200 mL LB for an overnight incubation in a 30 °C shaker. The next day, plasmids were isolated using the PureLink™ Expi Endotoxin-Free Maxi Plasmid Purification Kit (Invitrogen, A31217).

After library generation, sgRNA and tumor barcode diversity were determined by PCR amplification of the sgRNA and tumor barcode region of each plasmid library and sequencing using a MiSeq platform (Azenta Life Sciences). The primers were: Forward: 5′ CCGTAACTTGAAAGTATTTCGATTTCTTGGC 3′, Reverse: 5′ CGGTGCCACTTTTTCAAGTTG 3′.

To clone individual sgRNAs into each vector, the same cloning strategy was used. After the Golden Gate cloning reaction, samples were transformed using One Shot™ Stbl3™ Chemically Competent *E. coli* (Invitrogen, C737303). Individual colonies were picked to confirm the presence of the sgRNA. A single colony with a known tumor barcode sequence was then chosen and a bacterial stock was made.

### Cell lines

All cell lines were grown in DMEM supplemented with GlutaMAX (Invitrogen, 10566016), 10% fetal bovine serum, and gentamicin (Invitrogen, 15750060) at 37 °C and 5% CO_2_.

To create ‘spike in’ cells for Tuba-seq, LG1233 cells were transduced with Lenti:mCh/Cre-PuroR (derived from Lenti-CRISPRv2Puro—Addgene #98290) that contained a library of GFP-targeting sgRNAs with unique tumor barcode sequences. The GFP sgRNA sequence was: 5′ CAAGCAGAAGAACGGCATCA 3′. A GFP sgRNA was used since it was not included in the sgRNA library for Tuba-seq and could therefore be distinguished as benchmark DNA rather than tumor DNA. Cells were transduced in multiple wells of a 6 well plate using a serial dilution of virus in each well to limit the chance of multiple lentiviral particles transducing a single cell. Transduced cells were selected for using a Puromycin selection as described above. Once selection was complete, 1 × 10^3^ cells from each initial well were plated in a 15 cm plate to generate single clones. Each clone was selected and expanded until DNA could be collected using the Qiagen DNeasy Blood & Tissue Kit (Qiagen, 69504) as instructed. The barcode and sgRNA sequence were amplified from the genomic DNA using the following primer set: Forward: 5′ CCGTAACTTGAAAGTATTTCGATTTCTTGGC 3′, Reverse: 5′ CGGTGCCACTTTTTCAAGTTG 3′. PCR amplicons were Sanger sequenced to confirm the presence of the GFP sgRNA and to determine the tumor barcode sequence associated with each clone. 3 benchmark cell lines were established with the following tumor barcode sequences: CTATTAACAA, CCACTTTCCT, and TACATTATTA.

### Flow cytometry

Lungs were harvested from mice and 1-3 lobes per mouse were set aside for flow cytometry. For flow cytometry on tumor cells, lung tissue was cut into small pieces and 1 mL of digestion buffer was added to each sample. Each 1 mL of digestion buffer contained 700 uL HBSS media (-Mg^2+^ -Ca^2+^), 100 uL Trypsin-EDTA (0.25%), 100 uL Collagenase IV (Worthington Biochemical Corp., LS004188, from 10 mg/mL stock dissolved in HBSS), 0.5U Dispase (Corning, 354235, 5 U/mL stock). Samples were shaken at 37 °C for 45 min at 200 rpm to digest the tissue. Tissue was further dissociated using a p200 pipette. 2 mL of quench buffer was added to each sample to halt tissue digestion. Each 2 mL of Quench buffer contained 200 uL fetal bovine serum and 7.5 uL DNase (Roche Diagnostics, 10104159001, from 10 mg/mL stock dissolved in water) diluted in 1.8 mL Leibovitz’s L-15 media (Gibco, 11415064). For immunophenotyping of T cells, lung tissue was digested in a gentleMACS C tube (Miltenyi Biotec, 130-093-237) using the gentleMACS Octo Dissociator. Each 1 mL of digestion buffer contained 5 uL fetal bovine serum, 78 uL Collagenase IV (Worthington Biochemical Corp., LS004188, from 10 mg/mL stock dissolved in HBSS), and 4 uL DNase (Roche Diagnostics, 10104159001, from 10 mg/mL stock dissolved in water) dissolved in 913 uL HBSS. Each sample received 3.5 mL of digestion buffer, and double the volume of quench buffer (RPMI with 2% FBS) was used to stop the digestion.

All samples were then passed through a 45um filter and red blood cells were lysed with 1 mL ACK lysis buffer (Gibco, A1049201) for 5 min. The lysis reaction was then quenched with 9 mL PBS. Samples were then stained a cocktail of primary antibodies and viability dye for in the dark 30 min at 4 °C. LIVE/DEAD™ Fixable Olive (Invitrogen, L34977) or LIVE/DEAD™ Fixable Near IR (Invitrogen, L34992) were used as directed by the manufacturer. The antibodies used were: CD45 (1:200, eFluor 450, Invitrogen, 48-0451-82, clone 30-F11), H2Kb (1:200, BV510, Biolegend, 116523, clone AF6-88.5), H2Kb-SIINFEKL (1:100, APC, Biolegend, 141605, clone 25-D1.16), CD3 (1:100, PE-Cy7, BD Pharmingen, 552774, clone 145-2C11), CD4 (1:200, BV711, Invitrogen, 407-0042-80, clone RM4-5), CD8 (1:200, APC-eFluor 780, Invitrogen, 47-0081-82, clone 53-6.7), CD62-L (1:200, BV480, BD Biosciences, 746726, clone MEL-14), CD44 (1:200, PerCP-Cy5.5, Tonbo Biosciences, 65-0441-UO25, clone IM7), H2Kb-SIINFEKL Tetramer (1:200, Alexa Fluor 647, NIH Tetramer Core Facility), PD-1 (1:200, SuperBright 600, Invitrogen, 63-9985-80, clone J43), and CD39 (1:200, PE, Biolegend, 143803, clone Duha59).

Cells were then washed twice with FACS buffer. Finally, cells were resuspended in FACS buffer and flow cytometry was performed using an Attune NxT flow cytometer (Thermo Fisher) and the Attune Flow Cytometry Software v5.3.0. Data were analyzed in FlowJo v10.8.1. Tumor cells were gated on CD45^Negative^GFP^Positive^ live singlets (Fig. S3). Gating for T cell immunophenotyping is shown in Fig. S6.

### Lentiviral-based vector production

HEK 293FT cells were transfected with lentiviral plasmid, ∆8.2, and VSV-G plasmids in a 4:3:1 ratio diluted into Opti-MEM™ I Reduced Serum Medium (Invitrogen, 31985062). Polyethylenimine was used to increase the transfection efficiency. 24 hr after transfection, media was replaced with fresh DMEM supplemented with 25 mmol/L HEPES (Gibco, 15630-080) and 3 mmol/L caffeine (Sigma, C0750). The next day, virus media was collected from each plate and filtered through a 0.45um PVDF filter (Foxx Life Sciences, 378-3415-OEM). Then, virus media was centrifuged at 107,000xg for 2 h at 4 °C. The remaining virus media was poured out and virus pellets soaked overnight in 100 uL of PBS at 4 °C. After this, pellets were triturated and vortexed at 4 °C for 15 min before being centrifuged for 30 s at 16,000xg to remove debris. Lentivirus was aliquoted and stored at −80 °C for later use.

Lentiviruses were titered using Green-Go cells, which are derived from 3TZ cells and contain a Cre-dependent GFP reporter^50^. 2×10^5^ cells were plated in 6-well plates, and 24 hours later lentivirus was added at 10, 1, and 0.1 μL per well. Cells were analyzed by flow cytometry for GFP expression 48 hours following transduction, and viral titer was calculated accordingly.

For Tuba-seq, the plasmid sgRNA libraries generated for Lenti:Cre^BC-sgTS11-IM^, Lenti:mCh/Cre^BC-sgTS11-IM^, and Lenti:mCh-SIIN/Cre^BC-sgTS11-IM^ were individually made into lentivirus. The three viruses were titered separately and mixed prior to endotracheal administration.

### Genomic DNA Extraction for Tuba-seq

Lung lobes were harvested from mice and weighed. Then, each lung was placed into 10 mL of digestion buffer containing 100 mM NaCl, 20 mM Tris, 10 mM EDTA, 0.5% SDS, and 100 uL of 20 mg/mL Proteinase K as previously described^23^. 5×10^5^ cells from each of the three benchmark cell lines was added to each sample. Tissue was cut into small pieces and incubated at 55 °C overnight. Once tissue was digested the next day, DNA was extracted from 5 mL of each digested sample by phenol-chloroform extraction. DNA yield and quality was determined using a NanoDrop One^C^ (Thermo Fisher).

### Library preparation for sequencing

Library preparation occurred in two steps using a nested PCR strategy. First, the vID, BC and sgRNA sequences were amplified by PCR. Then, Illumina adapter sequences were added to each product in a second, shorter round of PCR.

To amplify the tumor barcode and sgRNA sequence in the first round of PCR, 32 ug of gDNA was used as template, split over 8 reactions with a final volume of 100 uL each. Q5 Hot Start High-Fidelity 2x Master Mix (New England Biolabs, M0494X) was mixed with gDNA and a universal forward and reverse primer set (Forward: 5′ CGTGACGTAGAAAGTAATAATTTCTTGGGTAG 3′, Reverse: 5′ GAGGCCGAATTCAAAAAAGCACC 3′). PCR conditions were as follows: 94 °C for 2 min, 94 °C for 30 s*, 57 °C for 30 s*, 68 °C for 30 s*, 72 °C for 7 min, then 4 °C indefinitely. Starred conditions were repeated for 32 cycles. A small volume of the final product was run on a 2% agarose gel to confirm barcode and sgRNA amplification. Then, a double size selection was performed on the remaining PCR product to remove gDNA and primer dimer using AmPure XP beads (Beckman Colter, A63881).

Next, a second PCR was used to add unique dual-indexed Illumina adapters to each mouse sample for sequencing. The unique dual-indexed primers contained the following general sequence: Forward: 5′- AATGATACGGCGACCACCGAGATCTACAC- 8nt i5 index-ACACTCTTTCCCTACACGACGCTCTTCCGATCT- 7-9 random nucleotides-CCGTAACTTGAAAGTATTTCG- 3′, Reverse: 5′- CAAGCAGAAGACGGCATACGAGAT-reverse complement of 8nt i7 index- GTGACTGGAGTTCAGACGTGTGCTCTTCCGATCT- 7-9 random nucleotides- CGGTGCCACTTTTTCAAGTTG- 3′. Illumina TruSeq i5 and i7 index sequences were used and each mouse received a unique combination of both index sequences. Each forward and reverse primer included 7-9 random nucleotides (indicated as N) directly upstream of the plasmid-binding region of the primer to counteract the limited nucleotide diversity in the amplicon library during sequencing, since no PhiX spike-in was used. To set up the PCR for each mouse in the experiment, Q5 Hot Start High-Fidelity 2x Master Mix was mixed with a unique indexed forward and reverse primer set and the size-selected amplicon from the previous PCR. The final reaction volume per tube was 100uL. PCR conditions were as follows: 94 °C for 2 min, 94 °C for 30 s*, 56 °C for 30 s*, 68 °C for 30 s*, 72 °C for 7 min, then 4 °C indefinitely. The starred conditions were repeated for 5 cycles to ensure Illumina adapter sequences were added without further amplifying the original PCR product. Reactions from each mouse were pooled into a single tube and 100uL of the pool was run on a 3% agarose gel. The band with the correct fragment length was cut from the gel and gel extracted using the Qiagen MinElute Gel Extraction Kit (Qiagen, 28604). Samples from each mouse were then pooled together and contaminants were removed from the pooled library using an ethanol precipitation. The final concentration and purity of the library was read on a NanoDrop One^C^ (Thermo Fisher) and samples were submitted for sequencing (Azenta Life Sciences). The pooled sample was sequenced on the Illumina HiSeq platform using a sequencing read length of 2 × 150 bp.

### Raw reads parsing and cleaning

Paired-end reads are first merged using AdapterRemoval^51^, and merged reads are parsed using regular expressions to identify the 4-nucleotide vID, 10-nucleotide BC and 20-nucleotide sgRNA sequence. For identifying sgRNA sequences, we required a perfect match with the designed sequences, as a mutated sgRNA could cause reduced efficiency or off-target effects. When we encountered low-frequency clonal barcodes within a 1-hamming distance of high-frequency clonal barcodes, we attributed them to sequencing or PCR errors (spurious reads). These low-frequency barcodes were merged with barcodes of higher frequencies. Finally, total reads for each vID-BC-sgRNA combination are calculated.

### Clonal tumor size estimation

Read counts associated with clonal tumors are converted into absolute neoplastic cell numbers by normalizing the BC read counts of tumor cells to the BC reads of the ‘spike-in’ cells added just before tissue digestion. For each sample, we check the read ratio among the three ‘spike-in’ cell lines. If one ‘spike-in’ cell line is under-amplified or over-sampled compared to the other two, we only used the other two to calculate the cell number conversion. Otherwise, all three ‘spike-in’ cell lines were used for the conversion. Samples were sequenced sufficiently deep such that across all experiments we achieved at least 15 cells per read. To perform statistical comparisons of tumor genotypes, we imposed a minimum tumor size cutoff of 100 cells to distinguish potential non-expansion cells, spurious tumors from sequencing errors and truly expanding tumors.

### Plasmid library quality control

To ensure equal representation of sgRNAs across Lenti:Cre^BC-sgTS11-IM^, Lenti:mCh/Cre^BC-sgTS11-IM^, and Lenti:mCh-SIIN/Cre^BC-sgTS11-IM^ libraries, we sequenced the plasmid libraries and confirmed that the read fractions for individual sgRNAs correlated highly across all three vectors (Pearson’s r ≥ 0.99, Fig. S10A–C). Furthermore, we validated that the sgRNA representation in tumors from *K* mice exhibited a strong correlation with plasmid representation of each genotype (Pearson’s r ≥ 0.95, Fig. S10D–F). Additionally, we ensured that all designed sgRNAs were present in the library and that the number of clonal barcodes associated with each sgRNA was sufficiently large.

### Filtering low quality samples

First, we removed one K mouse where Lenti:mCh-SIIN/Cre^BC-sgTS11-IM^ did not reduce tumor number or area as observed across other *K* and *K;Cas9* mice.

Then, we identified potential contamination during sample preparation by evaluating the overlap of Lenti:Cre^BC-sgTS11-IM^ tumors with shared vID-BC-sgRNA combinations (Figure S10G). A specific vID-BC-sgRNA combination appearing in more than one sample can either be attributed to random sampling events, as all tumors originate from the same pool of lentiviral particles, or to contamination between samples. If the overlap of vID-BC-sgRNA combinations is due to random sampling, the number of tumors with the same vID-BC-sgRNA combinations appearing in both samples should be predictable as a function of the total number of tumors in each sample. Therefore, we used the total tumor counts from both samples to predict the expected number of tumors with shared vID-BC-sgRNA combinations (Fig. S10G and H). Upon applying this approach, we identified a sample pair—sample 29791 and sample 29837—with a significantly higher number of shared vID-BC-sgRNA combinations than expected. Further investigation into the tumor sizes within these samples revealed two distinct groups: one that aligned well with the predicted overlap due to random sampling, and another indicative of contamination, specifically from sample 29791 to sample 29837 (Fig. S10I). Considering that ~50% of the tumors in sample 29837 exhibited shared vID-barcode-sgRNAs, we concluded that sample 29837 was severely contaminated by sample 29791. Due to the extent of the contamination, we decided to exclude sample 29837 from further analysis.

Finally, we identified samples in which gene knockouts had unexpected effects using principal component analysis (PCA) plots using both the relative geometric mean of tumor size and relative tumor counts as metrics. We identified one sample with a distinct pattern across both metrics and therefore excluded this sample from further analysis.

### Summary statistics for overall growth rate

To quantify the impact of each gene on tumor growth, we used three key measures: the size of tumors at defined percentiles of the distribution (specifically the 50^th^, 70^th^, 80^th^, 90^th^, and 95^th^ percentile tumor sizes), the log-normal mean (LN mean) size and geometric mean size^28,52^. To standardize the gene knockout effect across different immune contexts, we employed adaptive sampling^53^. Using tumors initiated by Lenti:Cre^BC-sgTS11-IM^ as the reference, this approach ensured an equal number of tumors were sampled per PFU of lentiviral-like virus for each sgRNA in each immune context, facilitating consistent calculation of tumor metrics.

### Summary statistics for tumor number and tumor burden

In addition to the tumor size metrics, we characterized the effects of gene inactivation on tumorigenesis by evaluating the number of tumors (“tumor number”) and total neoplastic cell number (“tumor burden”) associated with each genotype. Unlike tumor size metrics, tumor number and burden are linearly influenced by lentiviral titer and sensitive to differences in the representation of each immunogenic vector in the viral pool. As such, to assess the extent to which a given gene (*X)* affects tumor number, we therefore normalized the number of sg*X* tumors in *K;Cas9* mice by the number of sg*X* tumors in the *K* mice:

$$	{{{\rm{Tumor}}}}\; {{{\rm{number}}}}\; {{{\rm{for}}}}\; {{{\rm{sg}}}}X{{{\rm{tumors}}}}=\\ 	 \frac{{{{\rm{Number}}}}\, {{{\rm{of}}}}\; {{{\rm{sg}}}}X{{{\rm{tumors}}}}\; {{{\rm{in}}}}{K;Cas}9{{{\rm{mice}}}}}{{{{\rm{Number}}}}\; {{{\rm{of}}}}\; {{{\rm{sg}}}}{X}{{{\rm{tumors}}}}\; {{{\rm{in}}}}K{{{\rm{mice}}}}}$$

Analogous to the calculation of relative tumor number, we characterized the effect of each gene on tumor burden by first normalizing the sg*X* tumor burden in *K;Cas9* mice to the burden in *K* mice:

$$	{{{\rm{Tumor}}}}\; {{{\rm{burden}}}}\; {{{\rm{for}}}}\; {{{\rm{sg}}}}X{{{\rm{tumors}}}}=\\ 	 \frac{{{{\rm{Total}}}}\; {{{\rm{neoplastic}}}}\; {{{\rm{cell}}}}\; {{{\rm{number}}}}\; {{{\rm{of}}}}\; {{{\rm{sg}}}}X{{{\rm{tumors}}}}\; {{{\rm{in}}}}{K;Cas}9{{{\rm{mice}}}}}{{{{\rm{Total}}}}\; {{{\rm{neoplastic}}}}\; {{{\rm{cell}}}}\; {{{\rm{number}}}}\; {{{\rm{of}}}}\; {{{\rm{sg}}}}X{{{\rm{tumors}}}}\; {{{\rm{in}}}}K{{{\rm{mice}}}}}$$

### Model for genetic and immune effect on tumor metrics

We used a mathematical model to dissect the contributions of genetic context, immune response, and genotype-specific immune response to tumor fitness:

1 $${M}_{i,j}={M}_{0}\cdot {G}_{i}\cdot {I}_{j}\cdot G{I}_{i,j}$$

*M*_*i,j*_ is a tumor metric for tumor targeted by sgRNA *i* (refer as genotype *i* hereafter) under the immune context *j*. sg*Inert* (*i = Inert*) is the reference genotype and the immune context faced by the tumor initiated by Lenti:Cre^BC-sgTS11-IM^ (Control tumors) is the reference immune context. *M*_*0*_ is the baseline tumor metric for sg*Inert* tumor under the control context. *G*_*i*_ represents the genetic effect of genotype *i* on tumor fitness. *G*_*Inert*_ = 1 and a *G*_*i*_ > 1 indicate a tumor suppressor effect. *I*_*j*_ represents the effect of immune context *j* on tumor fitness. *I*_*Control*_ = 1 and a *I*_*j*_ < 1 indicates the immune context *j* suppresses tumor growth more than the control immune context. *GI*_*i,j*_ captures the interaction between the genotype *i* and the immune context *j* and indicates whether the genotype *i* confers resistance or susceptibility to the immune pressure*. GI*_*Inert,j*_ = *GI*_*i,Control*_ = 1. A *GI*_*i,j*_ > 1 indicates the focal genotype *i* is more resistant to the immune response than sg*Inert* tumor under the immune context *j*.

To measure the genetic effect of gene knockout (*G*_*i*_), we calculated the relative tumor metrics under the control immune context (*RM*_*i,j=Control*_):

2 $$R{M}_{i,j}=\frac{{M}_{i,j}}{{M}_{{Inert},j}}=\frac{{M}_{0}\cdot {G}_{i}\cdot {I}_{j}\cdot G{I}_{i,j}}{{M}_{0}\cdot {G}_{{Inert}}\cdot {I}_{j}\cdot G{I}_{{Inert},j}}={G}_{i}\cdot G{I}_{i,j}$$

When *j* = *Control*, Equation(2) becomes:

3 $$R{M}_{i,{Control}}={G}_{i}\cdot G{I}_{i,{control}}={G}_{i}$$

To measure the effect of the immune context (*I*_*j*_), we calculated the resistance (*R*_*Inert,j*_) of tumors to a specific immune context j by dividing a tumor metrics of sgInert tumors under the context *j* by the corresponding metrics under the immune control context:

4 $${R}_{{Inert},j}=\frac{{M}_{{Inert},j}}{{M}_{{Inert},{Control}}}=\frac{{M}_{0}\cdot {G}_{{Inert}}\cdot {I}_{j}\cdot G{I}_{{Inert},j}}{{M}_{0}\cdot {G}_{{Inert}}\cdot {I}_{{Control}}\cdot G{I}_{{Inert},{Control}}}={I}_{j}$$

To measure the genotype-specific response of genotype *i* under the immune context *j*, we calculated the relative resistance (*RR*) by diving the *RM* under the immune context *j* (Equation(2)) by the *RM* under the control immune context (Equation(3)):

5 $$R{R}_{i,j}=\frac{R{M}_{i,j}}{{{RM}}_{i,{Control}}}=\frac{{G}_{i}\cdot G{I}_{i,j}}{{G}_{i}}=G{I}_{i,j}$$

### Calculation of confidence intervals and *p*-values for tumor growth and number metrics

Confidence intervals and *p*-values were calculated using bootstrap resampling to capture variability both between and within mice. We used a two-step nested bootstrap: first resampling mice, then resampling tumors within each mouse, repeating this process 10,000 times. The 95% confidence intervals were derived from the 2.5th and 97.5th percentiles of the bootstrapped values.

The null hypothesis assumes no genotype effect on tumor growth, implying a test statistic equal to 1. Two-sided *p*-values were calculated as:

$$p=2\cdot \min \left\{\Pr \left(T > 1\right),\Pr \left(T > 1\right)\right\}$$

where *T* represents the test statistic and probabilities were empirically estimated from bootstrap resampling. To adjust for multiple testing, *p*-values were corrected using the Benjamini-Hochberg procedure^54^.

### Fitting linear and quadratic models to assess relationships between tumor size across immune contexts

To explore the relationship between tumor size and tumor number across different immune contexts for the same genotype, we performed linear and quadratic fittings. All genotypes, except *B2M* and *PD-L1*, were initially included in the analysis. Tumor sizes were log-transformed to normalize distributions and stabilize variance.

We began by fitting a quadratic model to the relationship between the LN mean tumor sizes of Lenti:mCh-SIIN/Cre^BC-sgTS11-IM^ tumors and Lenti:Cre^BC-sgTS11-IM^ tumors. To account for potential outliers, we applied the RANSAC (Random Sample Consensus) algorithm during model fitting, enabling robust identification of outlier genes. After removing outliers, both linear and quadratic models were refit to the remaining data. The performance of these models was evaluated using the coefficient of determination (R²) and residual analysis to assess goodness-of-fit and determine the appropriateness of each model. Similar approaches were used to model the relationship between LN mean tumor sizes of Lenti:mCh/Cre^BC-sgTS11-IM^ tumors and Lenti:Cre^BC-sgTS11-IM^ tumors.

### Reporting summary

Further information on research design is available in the Nature Portfolio Reporting Summary linked to this article.

## Supplementary information

Supplementary Information

Reporting Summary

Transparent Peer Review file

## Source data

Source Data

## Data Availability

The Tuba-seq data generated in this study have been deposited in the NCBI Gene Expression Omnibus (GEO) database under accession code: GSE294730 (https://www.ncbi.nlm.nih.gov/geo/query/acc.cgi?acc=GSE294730). Source data are provided with this paper.

## Code availability

Downstream data processing, statistical analyses, and visualization were performed using custom Python scripts (Python 3.12), using standard scientific computing libraries including NumPy, pandas, and SciPy. Data visualization was performed using matplotlib and seaborn. Raw sequencing data were processed using AdapterRemoval (v2.3.1) and Bartender (v1.1). Code is available at GitHub (https://github.com/JasperXuEvolution/UltraSeqImmune) and archived at Zenodo (10.5281/zenodo.19393965)^55^.

## References

[1] Shankaran, V. et al. IFNgamma and lymphocytes prevent primary tumour development and shape tumour immunogenicity. *Nature* **410**, 1107–1111 (2001). 11323675 10.1038/35074122

[2] Schreiber, R. D., Old, L. J. & Smyth, M. J. Cancer immunoediting: integrating immunity’s roles in cancer suppression and promotion. *Science* **331**, 1565–1570 (2011). 21436444 10.1126/science.1203486

[3] DuPage, M. et al. Endogenous T cell responses to antigens expressed in lung adenocarcinomas delay malignant tumor progression. *Cancer Cell* **19**, 72–85 (2011). 21251614 10.1016/j.ccr.2010.11.011 PMC3069809

[4] Chang, C. H. et al. Metabolic Competition in the Tumor Microenvironment Is a Driver of Cancer Progression. *Cell*. **162**, 1229–1241 (2015). 26321679 10.1016/j.cell.2015.08.016 PMC4864363

[5] Joshi, N. S. et al. Regulatory T Cells in Tumor-Associated Tertiary Lymphoid Structures Suppress Anti-tumor T Cell Responses. *Immunity* **43**, 579–590 (2015). 26341400 10.1016/j.immuni.2015.08.006 PMC4826619

[6] Lavin, Y. et al. Innate Immune Landscape in Early Lung Adenocarcinoma by Paired Single-Cell Analyses. *Cell* **169**, 750–765 e717 (2017). 28475900 10.1016/j.cell.2017.04.014 PMC5737939

[7] Burr, M. L. et al. An Evolutionarily Conserved Function of Polycomb Silences the MHC Class I Antigen Presentation Pathway and Enables Immune Evasion in Cancer. *Cancer Cell* **36**, 385–401 e388 (2019). 31564637 10.1016/j.ccell.2019.08.008 PMC6876280

[8] Rosenthal, R. et al. Neoantigen-directed immune escape in lung cancer evolution. *Nature* **567**, 479–485 (2019). 30894752 10.1038/s41586-019-1032-7 PMC6954100

[9] Zhao, Y. et al. B2M gene expression shapes the immune landscape of lung adenocarcinoma and determines the response to immunotherapy. *Immunology* **164**, 507–523 (2021). 34115389 10.1111/imm.13384 PMC8517590

[10] Skoulidis, F. et al. Co-occurring genomic alterations define major subsets of KRAS-mutant lung adenocarcinoma with distinct biology, immune profiles, and therapeutic vulnerabilities. *Cancer Discov.* **5**, 860–877 (2015). 26069186 10.1158/2159-8290.CD-14-1236 PMC4527963

[11] Koyama, S. et al. STK11/LKB1 Deficiency Promotes Neutrophil Recruitment and Proinflammatory Cytokine Production to Suppress T-cell Activity in the Lung Tumor Microenvironment. *Cancer Res*. **76**, 999–1008 (2016). 26833127 10.1158/0008-5472.CAN-15-1439 PMC4775354

[12] Dong, Z. Y. et al. Potential Predictive Value of TP53 and KRAS Mutation Status for Response to PD-1 Blockade Immunotherapy in Lung Adenocarcinoma. *Clin. Cancer Res*. **23**, 3012–3024 (2017). 28039262 10.1158/1078-0432.CCR-16-2554

[13] Skoulidis, F. et al. STK11/LKB1 Mutations and PD-1 Inhibitor Resistance in KRAS-Mutant Lung Adenocarcinoma. *Cancer Discov.* **8**, 822–835 (2018). 29773717 10.1158/2159-8290.CD-18-0099 PMC6030433

[14] Kitajima, S. et al. Suppression of STING Associated with LKB1 Loss in KRAS-Driven. *Lung Cancer Cancer Discov.* **9**, 34–45 (2019). 30297358 10.1158/2159-8290.CD-18-0689 PMC6328329

[15] Gutiontov, S. I. et al. CDKN2A loss-of-function predicts immunotherapy resistance in non-small cell lung cancer. *Sci. Rep.* **11**, 20059 (2021). 34625620 10.1038/s41598-021-99524-1 PMC8501138

[16] Liu, B. et al. RBM10 Deficiency Is Associated With Increased Immune Activity in Lung Adenocarcinoma. *Front Oncol.* **11**, 677826 (2021). 34367963 10.3389/fonc.2021.677826 PMC8336464

[17] Martin, T. D. et al. The adaptive immune system is a major driver of selection for tumor suppressor gene inactivation. *Science* **373**, 1327–1335 (2021). 34529489 10.1126/science.abg5784 PMC13397623

[18] Wang, Q. et al. RB1 aberrations predict outcomes of immune checkpoint inhibitor combination therapy in NSCLC. *Front Oncol.* **13**, 1172728 (2023). 37441425 10.3389/fonc.2023.1172728 PMC10334286

[19] Zavitsanou, A. M. et al. KEAP1 mutation in lung adenocarcinoma promotes immune evasion and immunotherapy resistance. *Cell Rep.* **42**, 113295 (2023). 37889752 10.1016/j.celrep.2023.113295 PMC10755970

[20] Jee, J. et al. Automated real-world data integration improves cancer outcome prediction. *Nature* **636**, 728–736 (2024). 39506116 10.1038/s41586-024-08167-5 PMC11655358

[21] Jackson, E. L. et al. Analysis of lung tumor initiation and progression using conditional expression of oncogenic K-ras. *Genes Dev.* **15**, 3243–3248 (2001). 11751630 10.1101/gad.943001 PMC312845

[22] Jackson, E. L. et al. The differential effects of mutant p53 alleles on advanced murine lung cancer. *Cancer Res*. **65**, 10280–10288 (2005). 16288016 10.1158/0008-5472.CAN-05-2193

[23] Rogers, Z. N. et al. A quantitative and multiplexed approach to uncover the fitness landscape of tumor suppression in vivo. *Nat. Methods* **14**, 737–742 (2017). 28530655 10.1038/nmeth.4297 PMC5495136

[24] McFadden, D. G. et al. Mutational landscape of EGFR-, MYC-, and Kras-driven genetically engineered mouse models of lung adenocarcinoma. *Proc. Natl. Acad. Sci. USA*. **113**, E6409–E6417 (2016). 27702896 10.1073/pnas.1613601113 PMC5081629

[25] Westcott, P. M. et al. The mutational landscapes of genetic and chemical models of Kras-driven lung cancer. *Nature* **517**, 489–492 (2015). 25363767 10.1038/nature13898 PMC4304785

[26] Murray, C. W. et al. LKB1 drives stasis and C/EBP-mediated reprogramming to an alveolar type II fate in lung cancer. *Nat. Commun.* **13**, 1090 (2022). 35228570 10.1038/s41467-022-28619-8 PMC8885825

[27] Rogers, Z. N. et al. Mapping the in vivo fitness landscape of lung adenocarcinoma tumor suppression in mice. *Nat. Genet* **50**, 483–486 (2018). 29610476 10.1038/s41588-018-0083-2 PMC6061949

[28] Cai, H. et al. A Functional Taxonomy of Tumor Suppression in Oncogenic KRAS-Driven Lung Cancer. *Cancer Discov.* **11**, 1754–1773 (2021). 33608386 10.1158/2159-8290.CD-20-1325 PMC8292166

[29] Foggetti, G. et al. Genetic Determinants of EGFR-Driven Lung Cancer Growth and Therapeutic Response In Vivo. *Cancer Discov.* **11**, 1736–1753 (2021). 33707235 10.1158/2159-8290.CD-20-1385 PMC8530463

[30] Blair, L. M. et al. Oncogenic context shapes the fitness landscape of tumor suppression. *Nat. Commun.* **14**, 6422 (2023). 37828026 10.1038/s41467-023-42156-y PMC10570323

[31] Y. J. Tang, et al., Functional mapping of epigenomic regulators uncovers coordinated tumor suppression by the HBO1 and MLL1 complexes. *Cancer Discov* (2025). 10.1158/2159-8290.CD-24-1565 PMC12469823 40997327

[32] Karlhofer, F. M., Ribaudo, R. K. & Yokoyama, W. M. MHC class I alloantigen specificity of Ly-49 + IL-2-activated natural killer cells. *Nature* **358**, 66–70 (1992). 1614533 10.1038/358066a0

[33] Long, E. O., Kim, H. S., Liu, D., Peterson, M. E. & Rajagopalan, S. Controlling natural killer cell responses: integration of signals for activation and inhibition. *Annu Rev. Immunol.* **31**, 227–258 (2013). 23516982 10.1146/annurev-immunol-020711-075005 PMC3868343

[34] Xue, W. et al. Senescence and tumour clearance is triggered by p53 restoration in murine liver carcinomas. *Nature* **445**, 656–660 (2007). 17251933 10.1038/nature05529 PMC4601097

[35] Guo, G. et al. Trp53 inactivation in the tumor microenvironment promotes tumor progression by expanding the immunosuppressive lymphoid-like stromal network. *Cancer Res*. **73**, 1668–1675 (2013). 23319800 10.1158/0008-5472.CAN-12-3810 PMC3602383

[36] Guo, G., Yu, M., Xiao, W., Celis, E. & Cui, Y. Local Activation of p53 in the Tumor Microenvironment Overcomes Immune Suppression and Enhances Antitumor Immunity. *Cancer Res*. **77**, 2292–2305 (2017). 28280037 10.1158/0008-5472.CAN-16-2832 PMC5465961

[37] Chollat-Namy, M. et al. The pharmalogical reactivation of p53 function improves breast tumor cell lysis by granzyme B and NK cells through induction of autophagy. *Cell Death Dis.* **10**, 695 (2019). 31541080 10.1038/s41419-019-1950-1 PMC6754511

[38] Blagih, J. et al. Cancer-Specific Loss of p53 Leads to a Modulation of Myeloid and T Cell Responses. *Cell Rep.* **30**, 481–496 e486 (2020). 31940491 10.1016/j.celrep.2019.12.028 PMC6963783

[39] Ghosh, M., Saha, S., Li, J., Montrose, D. C. & Martinez, L. A. p53 engages the cGAS/STING cytosolic DNA sensing pathway for tumor suppression. *Mol. Cell* **83**, 266–280 e266 (2023). 36638783 10.1016/j.molcel.2022.12.023 PMC9993620

[40] Atsumi, T. et al. Rbm10 regulates inflammation development via alternative splicing of Dnmt3b. *Int Immunol.* **29**, 581–591 (2017). 29309623 10.1093/intimm/dxx067

[41] Pozzi, B. et al. Dengue virus targets RBM10 deregulating host cell splicing and innate immune response. *Nucleic Acids Res*. **48**, 6824–6838 (2020). 32432721 10.1093/nar/gkaa340 PMC7337517

[42] Li, L., Li, M., Jiang, Z. & Wang, X. ARID1A Mutations Are Associated with Increased Immune Activity in Gastrointestinal Cancer. *Cells* **8**, 678 (2019). 31277418 10.3390/cells8070678 PMC6678467

[43] Okamura, R. et al. ARID1A alterations function as a biomarker for longer progression-free survival after anti-PD-1/PD-L1 immunotherapy. *J. Immunother. Cancer* **8**, e000438 (2020). 32111729 10.1136/jitc-2019-000438 PMC7057434

[44] Maxwell, M. B. et al. ARID1A suppresses R-loop-mediated STING-type I interferon pathway activation of anti-tumor immunity. *Cell* **187**, 3390–3408 e3319 (2024). 38754421 10.1016/j.cell.2024.04.025 PMC11193641

[45] Fox, D. B. et al. Downregulation of KEAP1 in melanoma promotes resistance to immune checkpoint blockade. *NPJ Precis Oncol.* **7**, 25 (2023). 36864091 10.1038/s41698-023-00362-3 PMC9981575

[46] Occhiuto, C. J. & Liby, K. T. KEAP1-Mutant Lung Cancers Weaken Anti-Tumor Immunity and Promote an M2-like Macrophage Phenotype. *Int J. Mol. Sci.* **25**, 3510 (2024). 38542481 10.3390/ijms25063510 PMC10970780

[47] F. Skoulidis, et al., CTLA4 blockade abrogates KEAP1/STK11-related resistance to PD-(L)1 inhibitors. *Nature* (2024). 10.1038/s41586-024-07943-7 PMC11560846 39385035

[48] DuPage, M., Dooley, A. L. & Jacks, T. Conditional mouse lung cancer models using adenoviral or lentiviral delivery of Cre recombinase. *Nat. Protoc.* **4**, 1064–1072 (2009). 19561589 10.1038/nprot.2009.95 PMC2757265

[49] Crowe, A. R. & Yue, W. Semi-quantitative Determination of Protein Expression using Immunohistochemistry Staining and Analysis: An Integrated Protocol. *Bio Protoc.* **9**, e3465 (2019). 31867411 10.21769/BioProtoc.3465 PMC6924920

[50] Sanchez-Rivera, F. J. et al. Rapid modelling of cooperating genetic events in cancer through somatic genome editing. *Nature* **516**, 428–431 (2014). 25337879 10.1038/nature13906 PMC4292871

[51] Schubert, M., Lindgreen, S. & Orlando, L. AdapterRemoval v2: rapid adapter trimming, identification, and read merging. *BMC Res Notes* **9**, 88 (2016). 26868221 10.1186/s13104-016-1900-2 PMC4751634

[52] Li, C. et al. Quantitative In Vivo Analyses Reveal a Complex Pharmacogenomic Landscape in Lung Adenocarcinoma. *Cancer Res*. **81**, 4570–4580 (2021). 34215621 10.1158/0008-5472.CAN-21-0716 PMC8416777

[53] E. G. Shuldiner, et al, Aging represses lung tumorigenesis and alters tumor suppression. *bioRxiv* (2024). 10.1038/s43587-025-00986-z PMC12616358 41188600

[54] Benjamini, Y. & Hochberg, Y. Controlling the False Discovery Rate - a Practical and Powerful Approach to Multiple Testing. *J. R. Stat. Soc. Ser. B-Stat. Methodol.* **57**, 289–300 (1995).

[55] H. Xu, Ultra-Seq Immunoediting Analysis. Zenodo. 10.5281/zenodo.19393965 (2026).

