# Supplementary material for: Tumor suppressor genotype influences the extent and mode of immunosurveillance in lung cancer
Source: Nat Commun. 2026 Jun 15;17:7534. doi: 10.1038/s41467-026-74023-x (PMC13408662; doi:10.1038/s41467-026-74023-x)
Supplement: Supplementary file 1 — Supplementary Information [file 41467_2026_74023_MOESM1_ESM.pdf]

Tumor suppressor genotype influences the extent and mode of immunosurveillance in lung cancer

Authors: Keren M. Adler[†,1,2,3], Haiqing Xu[†,4,5], Amy C. Gladstein[1,2,3], Valerie M. Irizarry-Negron[1,2,3], Maggie R. Robertson[1,2], Katherine R. Doerig[1,2,3], Dmitri A. Petrov[5], Monte M. Winslow[*4,5,6] and David M. Feldser[*,1,2,3]

Affiliations:
[1]Department of Cancer Biology, Perelman School of Medicine, University of Pennsylvania; Philadelphia, PA, USA.

[2]Abramson Family Cancer Research Institute, Perelman School of Medicine, University of Pennsylvania; Philadelphia, PA, USA.

[3]Cell and Molecular Biology Graduate Group, Perelman School of Medicine, University of Pennsylvania; Philadelphia, PA, USA.

[4]Department of Genetics, Stanford University School of Medicine; Stanford, CA, USA.

[5]Department of Biology, Stanford University School of Medicine; Stanford, CA, USA.

[6]Department of Pathology, Stanford University School of Medicine; Stanford, CA, USA.

[†] Equal Contribution
[*] Correspondence: mwinslow@stanford.edu, dfeldser@upenn.edu

Supplementary Information

Supplementary Figures 1-14

SUPPLEMENTARY FIGURES

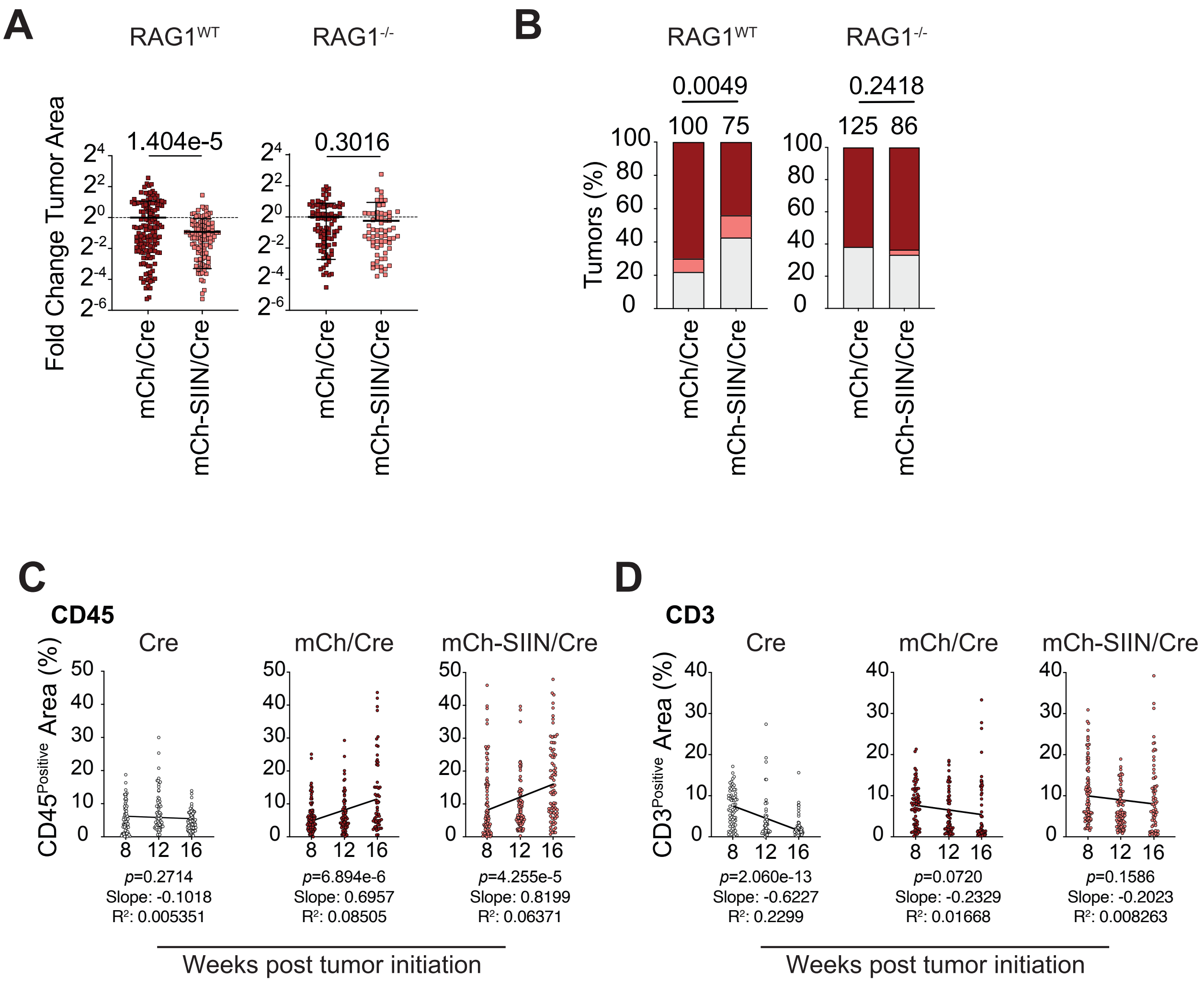

Fig. S1. An adaptive immune response is required to drive SIINFEKL-mediated immunoediting. A. Fold change in tumor area in RAG1^{WT} mice versus RAG1$^{-/-}$ mice, graphed using a Log_2 scale. Significance was determined using Student's *t* tests. n=10 mice per group for mCh/Cre-TS^{WT}, and n=9 mice per group for mCh-SIIN/Cre (*K;Cas9* mice). n=5 mice per group for mCh/Cre, and n=4 mice per group for mCh-SIIN/Cre (*K;Cas9;Rag1$^{-/-}$* mice). **B.** Proportion of mCherry^{Positive} tumors initiated by Lenti:mCh/Cre and Lenti:mCh-SIIN/Cre in RAG1^{WT} mice versus RAG1$^{-/-}$ mice. Significance was determined using Fisher's exact tests. Mouse number per group is the same as denoted in Fig. S1C. **C and D.** Quantification of CD45 (A) or CD3 (B) IHC as shown in Fig. 2, C and D with linear regression overlay.

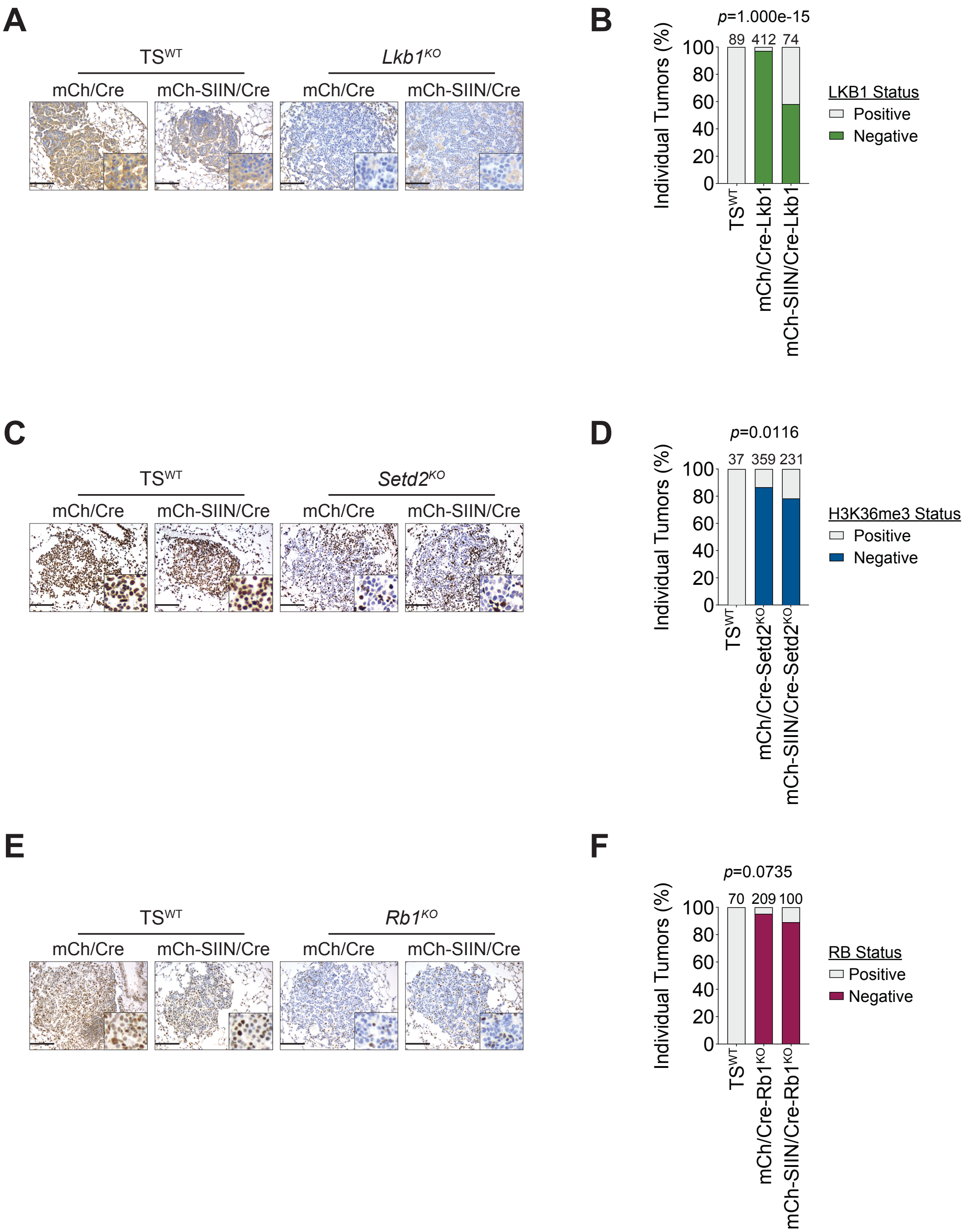

Fig. S2. Functional validation of CRISPR-mediated inactivation of *Lkb1*, *Rb1*, or *Setd2* in lung tumors. A. IHC for LKB1 in *K;Cas9* mice harboring no gene inactivation (TS^{WT}, left) or *Lkb1* inactivation ($Lkb1^{KO}$, right). Representative images are 20x with 3x magnified insets. Scale bar is 119um. **B.** Qualitative assessment of LKB1 expression by IHC to determine knockout efficiency. Significance was determined using a Chi-square test between mCh/Cre and mCh-SIIN/Cre $Lkb1^{KO}$ groups. Number of individual tumors analyzed is indicated on graph. n=10 mice per group for mCh/Cre-$Lkb1^{KO}$, and n=8 mice per group for mCh-SIIN/Cre-$Lkb1^{KO}$. For TS^{WT} analysis, n=1 mCh/Cre mouse and n=1 mCh-SIIN/Cre mouse (used as a positive staining controls). **C.** IHC for H3K36me3 in *K;Cas9* mice harboring no gene inactivation (TS^{WT}, left) or *Setd2* inactivation ($Setd2^{KO}$, right). Representative images are 20x with 3x magnified insets.

Scale bar is 119um. **D.** Qualitative assessment of H3K36me3 expression by IHC to determine *Setd2* knockout efficiency. Significance was determined using a Chi-square test between mCh/Cre and mCh-SIIN/Cre $Setd2^{KO}$ groups. Number of individual tumors analyzed is indicated on graph. n=10 mice per group for mCh/Cre-$Setd2^{KO}$, and n=8 mice per group for mCh-SIIN/Cre-$Setd2^{KO}$. For TS^{WT} analysis, n=1 mCh/Cre mouse and n=1 mCh-SIIN/Cre mouse (used as a positive staining controls). **E.** IHC for RB in *K;Cas9* mice harboring no gene inactivation (TS^{WT}, left) or *Rb1* inactivation ($Rb1^{KO}$, right). Representative images are 20x with 3x magnified insets. Scale bar is 119um. **F.** Qualitative assessment of RB expression by IHC to determine knockout efficiency. Significance was determined using a Chi-square test between mCh/Cre and mCh-SIIN/Cre $Rb1^{KO}$ groups. Number of individual tumors analyzed is indicated on graph. n=9 mice per group for mCh/Cre-$Rb1^{KO}$, and n=8 mice per group for mCh-SIIN/Cre-$Rb1^{KO}$. For TS^{WT} analysis, n=1 mCh/Cre mouse and n=1 mCh-SIIN/Cre mouse (used as a positive staining controls).

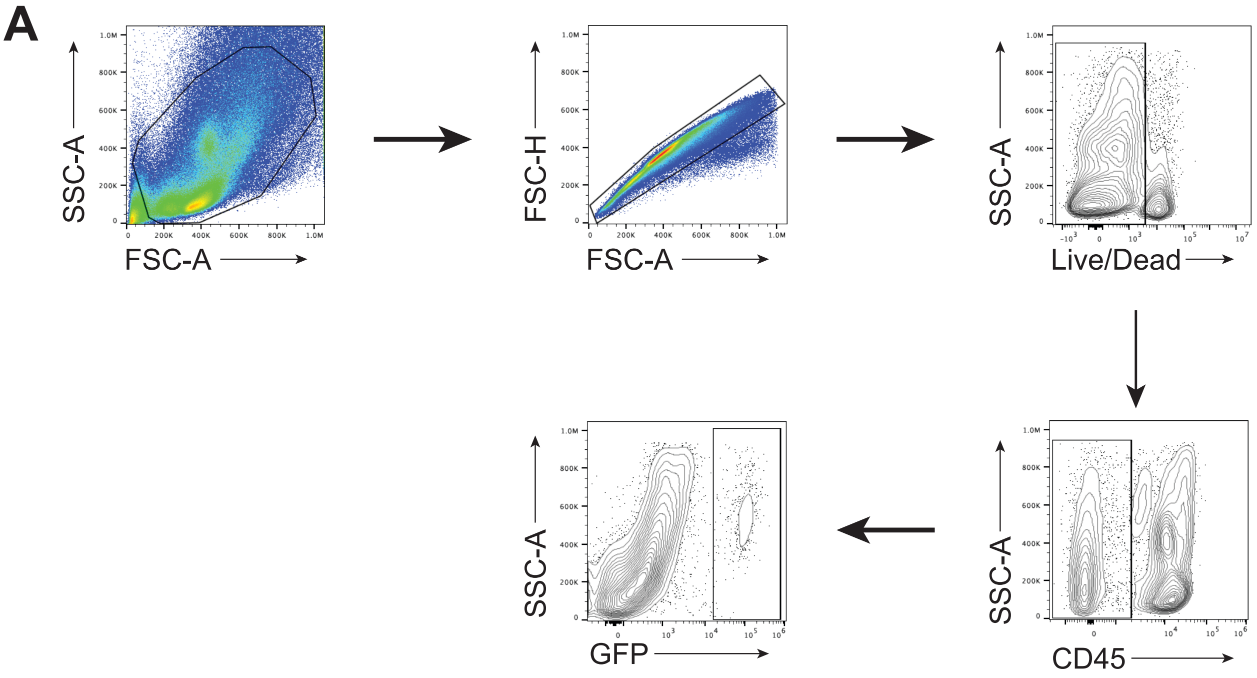

Fig. S3. Gating scheme for *in vivo* analysis of neoplastic cells. A. Gating strategy for flow cytometry to analyze GFP^{Positive} neoplastic cells.

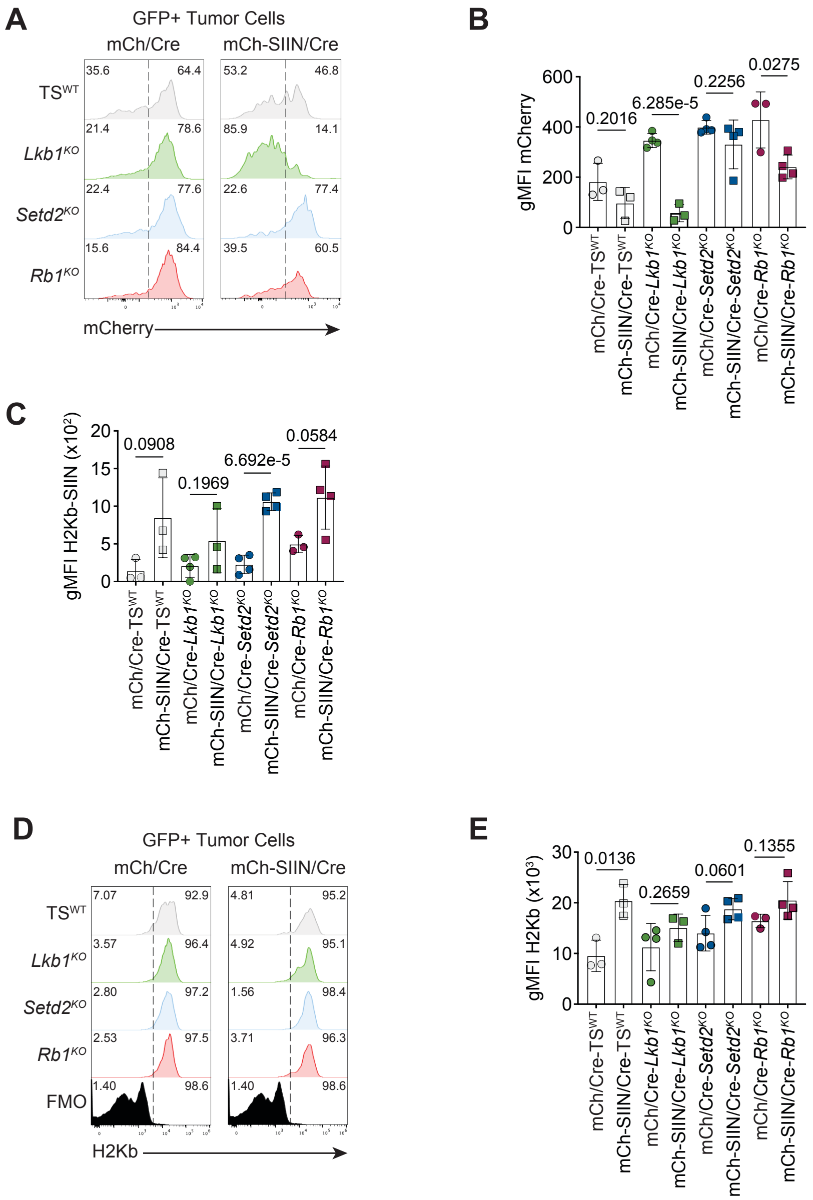

Fig. S4. Tumors with *Setd2*^{KO} or *Rb1*^{KO} maintain mCherry expression and antigen presentation in a highly immunogenic context. A. Histogram plots showing mCherry expression measured by flow cytometry across each genotype specified. Plots are separated based on initiating vector and are normalized to mode. Dotted line separates positive and negative populations and value in the upper corner indicates the percentage of cells in that population. **B.** Quantification of gMFI of mCherry expression as measured by flow cytometry, corresponding to the flow plots shown in Supplementary Figure 2A. Statistical significance was determined using unpaired Student's *t*-tests. Error bars represent mean ± standard deviation. n=4 for all experimental groups except for mCh/Cre-TS^{WT}, mCh/Cre-*Rb1*^{KO}, mCh-SIIN/Cre-TS^{WT}, and mCh-SIIN/Cre-*Lkb1*^{KO} (n=3). **C.** Quantification of gMFI of H2Kb-SIINFEKL presentation as measured by flow cytometry, corresponding to the flow plots shown in Figure 3G. Statistical significance was determined using unpaired Student's *t*-tests. Error bars represent mean ± standard deviation. n=4 for all experimental groups except for mCh/Cre-TS^{WT}, mCh/Cre-*Rb1*^{KO}, mCh-SIIN/Cre-TS^{WT}, and mCh-SIIN/Cre-*Lkb1*^{KO} (n=3). **D.** Histograms

showing H2Kb presentation on tumor cells measured by flow cytometry across each genotype specified. Plots are separated based on initiating vector and normalized to mode. Dotted line separates positive and negative populations and value in the upper corner indicates the percentage of cells in that population. **E.** gMFI of H2Kb presentation as measured by flow cytometry. Statistical significance was determined using unpaired Student's *t*-tests. Error bars represent mean ± standard deviation. n=4 for all experimental groups except for mCh/Cre-TS^{WT}, mCh/Cre-$Rb1^{KO}$, mCh-SIIN/Cre-TS^{WT}, and mCh-SIIN/Cre-$Lkb1^{KO}$ (n=3).

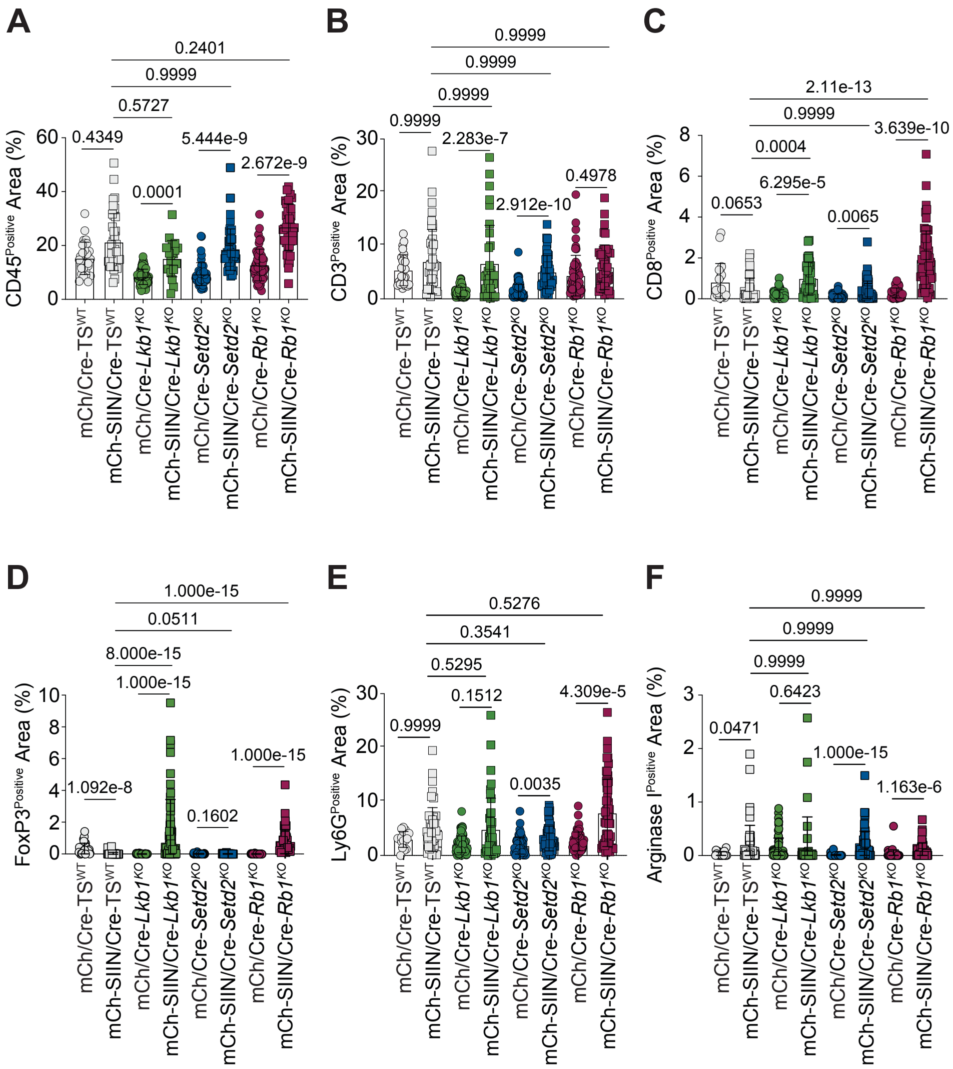

Fig. S5. SIINFEKL promotes immune infiltration across tumor suppressor genotypes. Statistical significance for all IHC quantifications in Fig. S5 were determined using Kruskal-Wallis tests. Error bars represent mean ± standard deviation. **A.** Percent CD45 positive tumor area. mCh/Cre-TS^{WT} n=28 tumors from n=4 mice. mCh-SIIN/Cre-TS^{WT} n=37 tumors from n=4 mice. mCh/Cre-*Lkb1*^{KO} n= 76 tumors from n=4 mice. mCh-SIIN/Cre-*Lkb1*^{KO} n=20 tumors from n=4 mice. mCh/Cre-*Setd2*^{KO} n= 45 tumors from n=4 mice. mCh-SIIN/Cre-*Setd2*^{KO} n=45 tumors from n=4 mice. mCh/Cre-*Rb1*^{KO} n= 49 tumors from n=3 mice. mCh-SIIN/Cre-*Rb1*^{KO} n=42 tumors from n=4 mice. **B.** Percent CD3 positive tumor area. mCh/Cre-TS^{WT} n=27 tumors from n=4 mice. mCh-SIIN/Cre-TS^{WT},n=45 tumors from n=4 mice. mCh/Cre-*Lkb1*^{KO} n= 73 tumors from n=4 mice. mCh-SIIN/Cre-*Lkb1*^{KO} n=34 tumors from n=4 mice. mCh/Cre-*Setd2*^{KO} n= 65 tumors from n=4 mice. mCh-SIIN/Cre-*Setd2*^{KO} n=42 tumors from n=4 mice. mCh/Cre-*Rb1*^{KO} n= 52 tumors from n=3 mice. mCh-SIIN/Cre-*Rb1*^{KO}, n=45 tumors from n=4 mice. **C.** Percent CD8 positive tumor area. mCh/Cre-TS^{WT} n=21 tumors from n=4 mice. mCh-SIIN/Cre-TS^{WT} n=50 tumors from n=4 mice. mCh/Cre-*Lkb1*^{KO} n= 72 tumors from n=4 mice. mCh-SIIN/Cre-*Lkb1*^{KO} n= 37 tumors from n=4 mice. mCh/Cre-*Setd2*^{KO} n= 68 tumors from n=4 mice. mCh-SIIN/Cre-*Setd2*^{KO} n=65 tumors from n=4 mice. mCh/Cre-*Rb1*^{KO} n= 38 tumors from n=3 mice. mCh-SIIN/Cre-*Rb1*^{KO} n=59 tumors from n=4 mice. **D.** Percent FoxP3 positive tumor area. mCh/Cre-

TS^{WT} n=36 tumors from n=4 mice. mCh-SIIN/Cre-TS^{WT},n=53 tumors from n=4 mice. mCh/Cre-*Lkb1*^{KO} n= 67 tumors from n=4 mice. mCh-SIIN/Cre-*Lkb1*^{KO} n= 63 tumors from n=4 mice. mCh/Cre-*Setd2*^{KO} n= 57 tumors from n=4 mice. mCh-SIIN/Cre-*Setd2*^{KO} n=49 tumors from n=4 mice. mCh/Cre-*Rb1*^{KO} n= 52 tumors from n=3 mice. mCh-SIIN/Cre-*Rb1*^{KO} n=54 tumors from n=4 mice. **E.** Percent Ly6G positive tumor area. mCh/Cre-TS^{WT} n=17 tumors from n=4 mice. For mCh-SIIN/Cre-TS^{WT}, n=49 tumors from n=4 mice. For mCh/Cre-*Lkb1*^{KO}, n= 81 tumors from n=4 mice. mCh-SIIN/Cre-*Lkb1*^{KO} n=42 tumors from n=4 mice. mCh/Cre-*Setd2*^{KO} n=68 tumors from n=4 mice. mCh-SIIN/Cre-*Setd2*^{KO} n=65 tumors from n=4 mice. mCh/Cre-*Rb1*^{KO} n= 53 tumors from n=3 mice. mCh-SIIN/Cre-*Rb1*^{KO} n=55 tumors from n=4 mice. **F.** Percent Arginase I positive tumor area. mCh/Cre-TS^{WT} n=20 tumors from n=4 mice. mCh-SIIN/Cre-TS^{WT} n=52 tumors from n=4 mice. mCh/Cre-*Lkb1*^{KO} n= 78 tumors from n=4 mice. mCh-SIIN/Cre-*Lkb1*^{KO} n=36 tumors from n=4 mice. mCh/Cre-*Setd2*^{KO} n=68 tumors from n=4 mice. mCh-SIIN/Cre-*Setd2*^{KO} n=67 tumors from n=4 mice. mCh/Cre-*Rb1*^{KO} n=47 tumors from n=3 mice. mCh-SIIN/Cre-*Rb1*^{KO} n=51 tumors from n=4 mice.

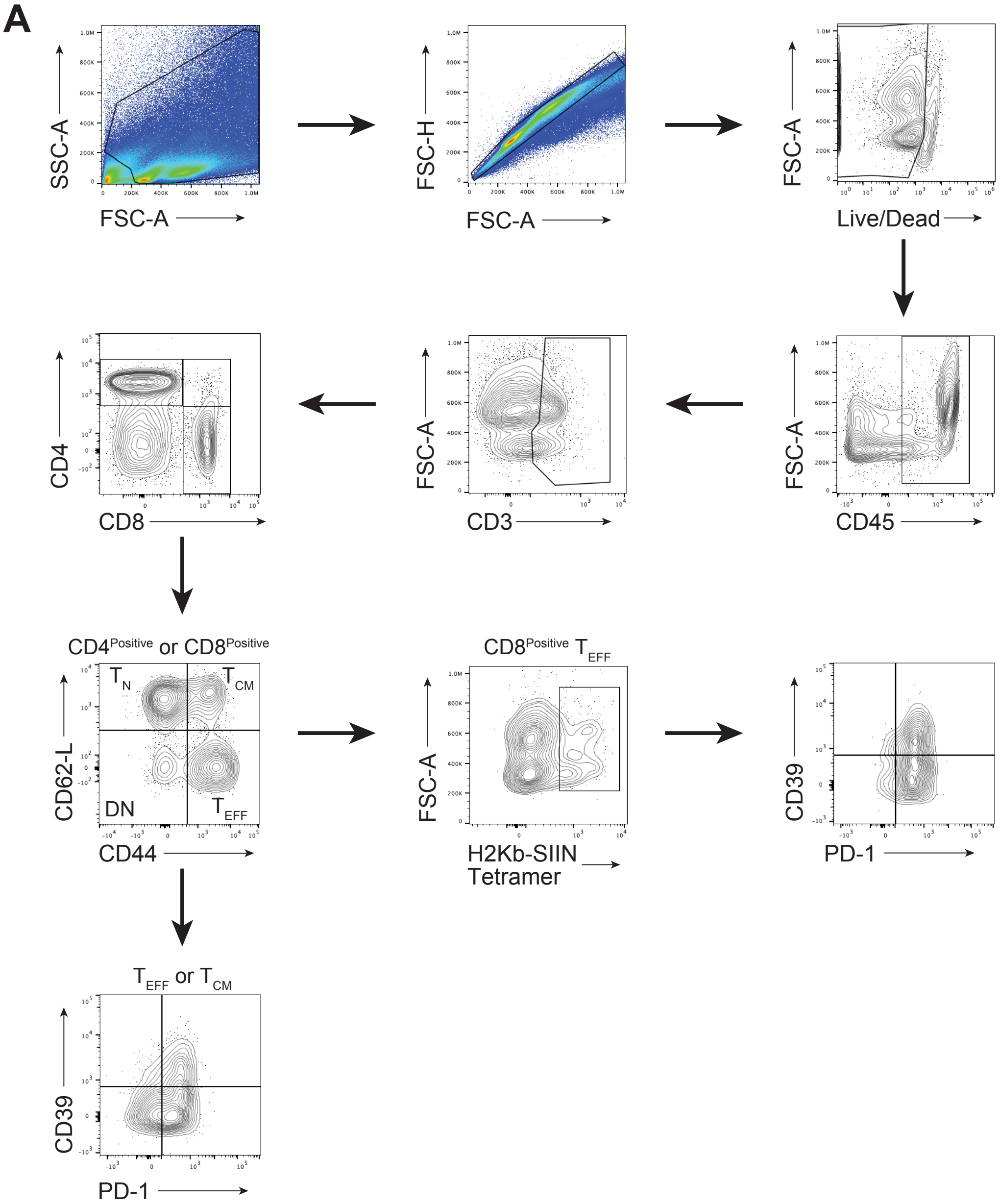

Fig. S6. Gating scheme for *in vivo* assessment of T cell phenotypes in response to potent neoantigen expression. A. Gating strategy for flow cytometry to analyze T cell subsets from lungs of tumor-bearing mice.

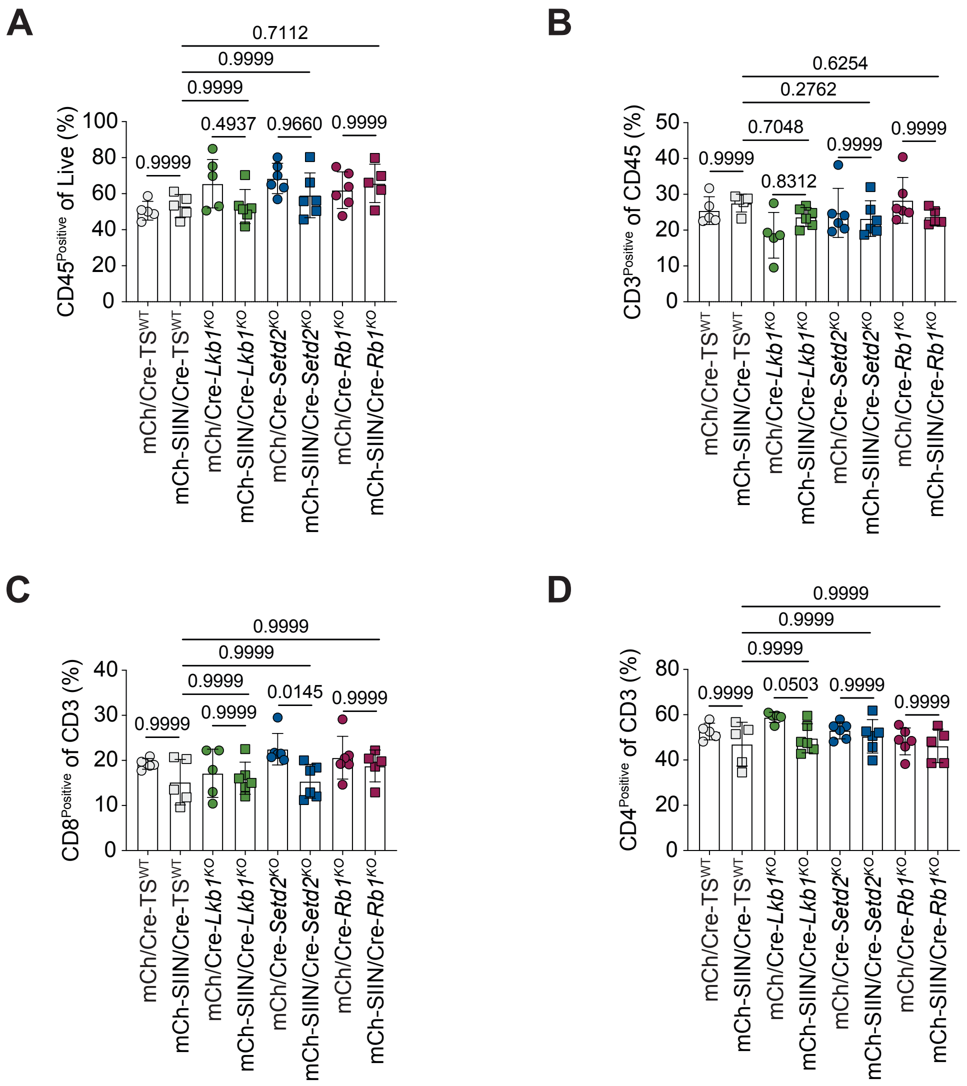

Fig. S7. Recruitment of T cells to tumor-bearing lungs upon potent neoantigen expression. A to D. Percentage of CD45^{Positive} (A), CD3^{Positive} (B), CD8^{Positive} (C), and CD4^{Positive} (D) immune cells in lungs of tumor-bearing mice as measured by flow cytometry. Statistical significance was determined using Kruskal-Wallis tests. Error bars represent mean ± standard deviation. n=6 for mCh-SIIN/Cre-*Lkb1*^{KO}, mCh/Cre-*Setd2*^{KO}, mCh-SIIN/Cre-*Setd2*^{KO}, and mCh/Cre-*Rb1*^{KO}. n=5 for mCh/Cre-TS^{WT}, mCh-SIIN/Cre-TS^{WT}, mCh/Cre-*Lkb1*^{KO}, and mCh-SIIN/Cre-*Rb1*^{KO}.

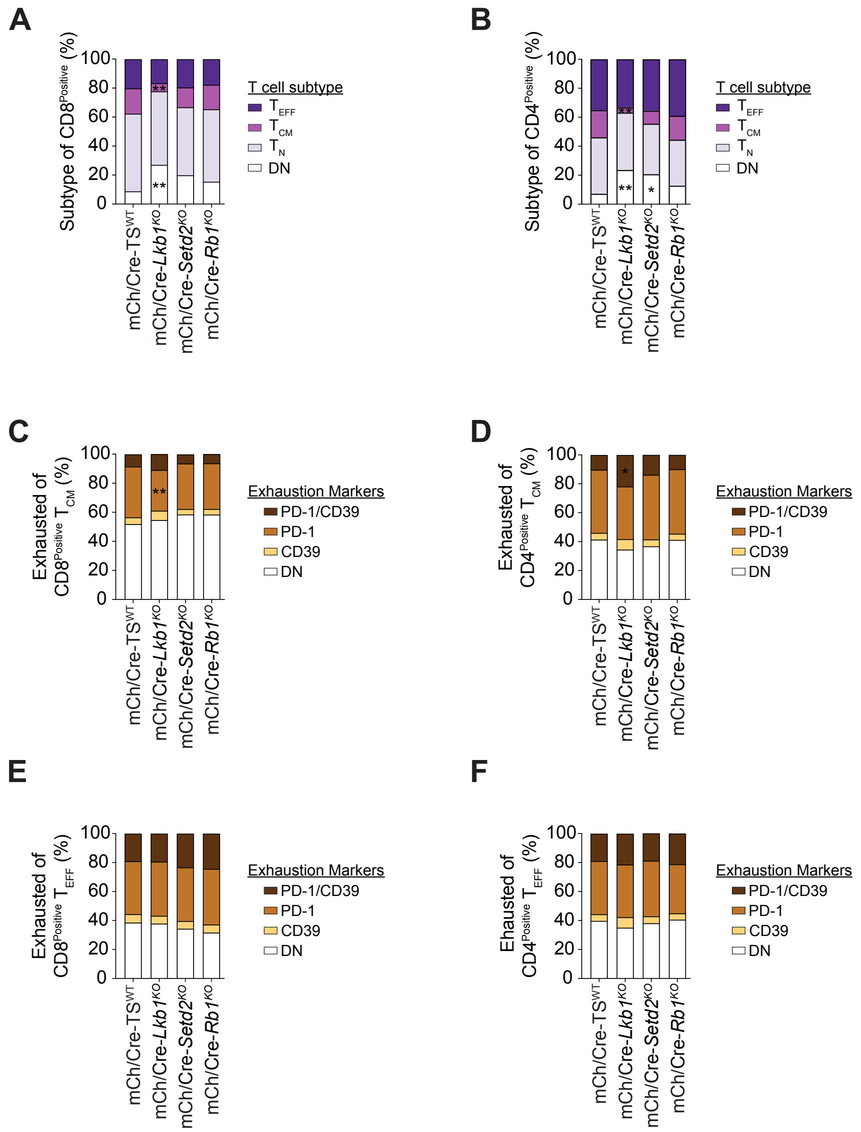

Fig. S8. Exhaustion of SIIN-nonspecific T cell subtypes across tumor suppressor genotypes. A and B. Stacked plot representing CD8^{Positive} (A) or CD4^{Positive} (B) T cells broken down into T_{EFF}, T_{CM}, T_N, and DN subsets, as measured by flow cytometry using CD44 and CD62-L. Plot shows mean value per exhausted subtype, but all individual values were used to calculate statistical significance by Kruskal-Wallis tests. Asterisk indicates level of significance. n=6 for mCh-mCh/Cre-*Setd2*^{KO} and mCh/Cre-*Rb1*^{KO}. n=5 for mCh/Cre-TS^{WT} and mCh/Cre-*Lkb1*^{KO}. **C to F.** Stacked plot representing exhaustion of different T cell subsets as measured by flow cytometry using PD-1 and CD39 markers. Exhausted CD8^{Positive} T_{CM} are shown in C, and exhausted CD4^{Positive} T_{CM} are shown in D. Exhausted CD8^{Positive} T_{EFF} are shown in E, and exhausted CD4^{Positive} T_{EFF} are shown in F. Plot shows mean percentage per exhausted subtype, but all individual values were used to calculate statistical significance by Kruskal-Wallis tests. Asterisk indicates level of significance. n=6 for mCh-mCh/Cre-*Setd2*^{KO} and mCh/Cre-*Rb1*^{KO}. n=5 for mCh/Cre-TS^{WT} and mCh/Cre-*Lkb1*^{KO}.

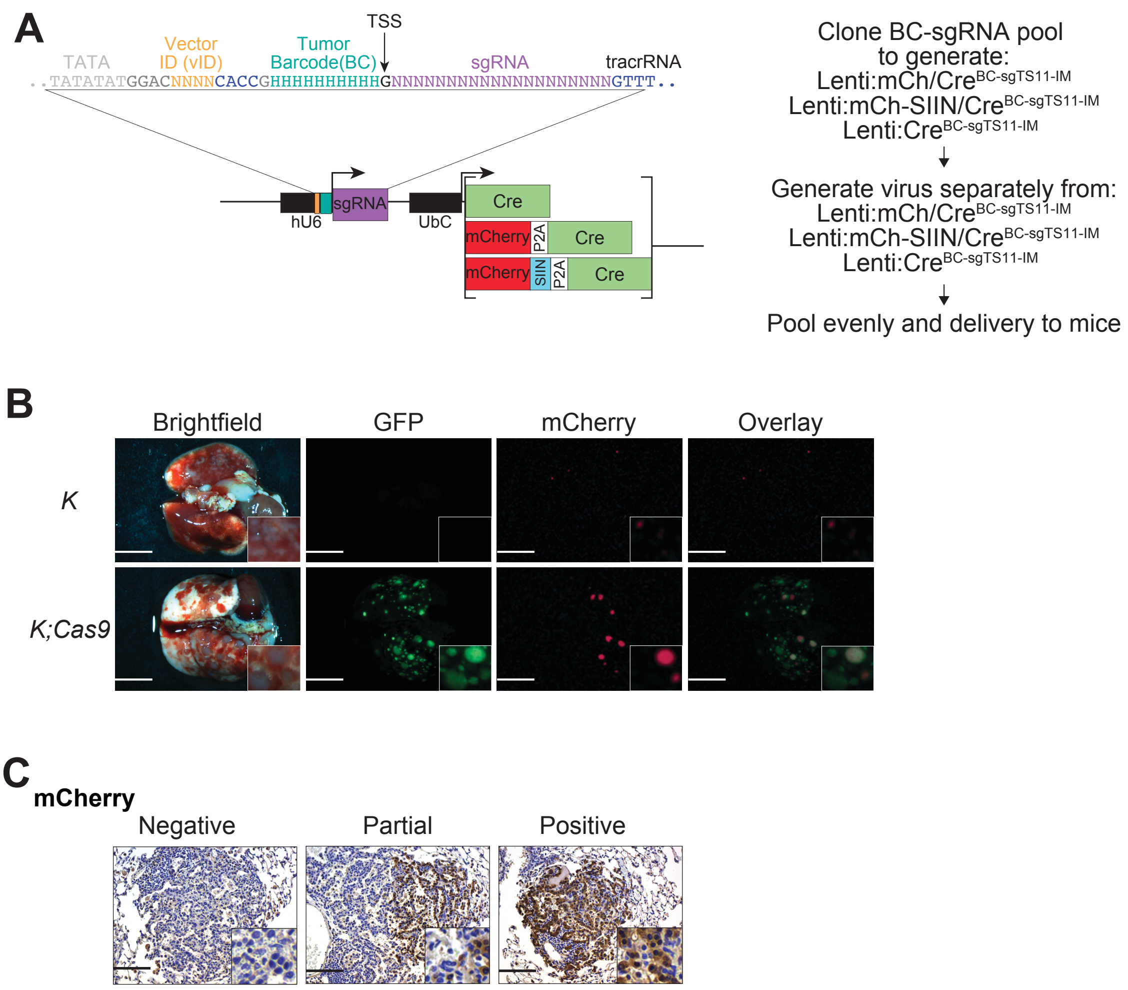

Fig. S9. The BC-sgTS11-IM pool sufficiently promotes tumor outgrowth and a subset of tumors are mCherry^{Positive}. A. Outline of strategy to generate lentiviral vector pools for Tuba-seq. **B.** Bright field, GFP, and mCherry images of whole dissected lungs. An overlay of the GFP and mCherry images is shown on the far right. Scale bar is 4.4mm. **C.** IHC for mCherry in *K* and *K;Cas9* mice. 20x Representative images of different mCherry expression statuses: negative, partial, or positive. Insets are 3x magnified. Scale bar is 119um.

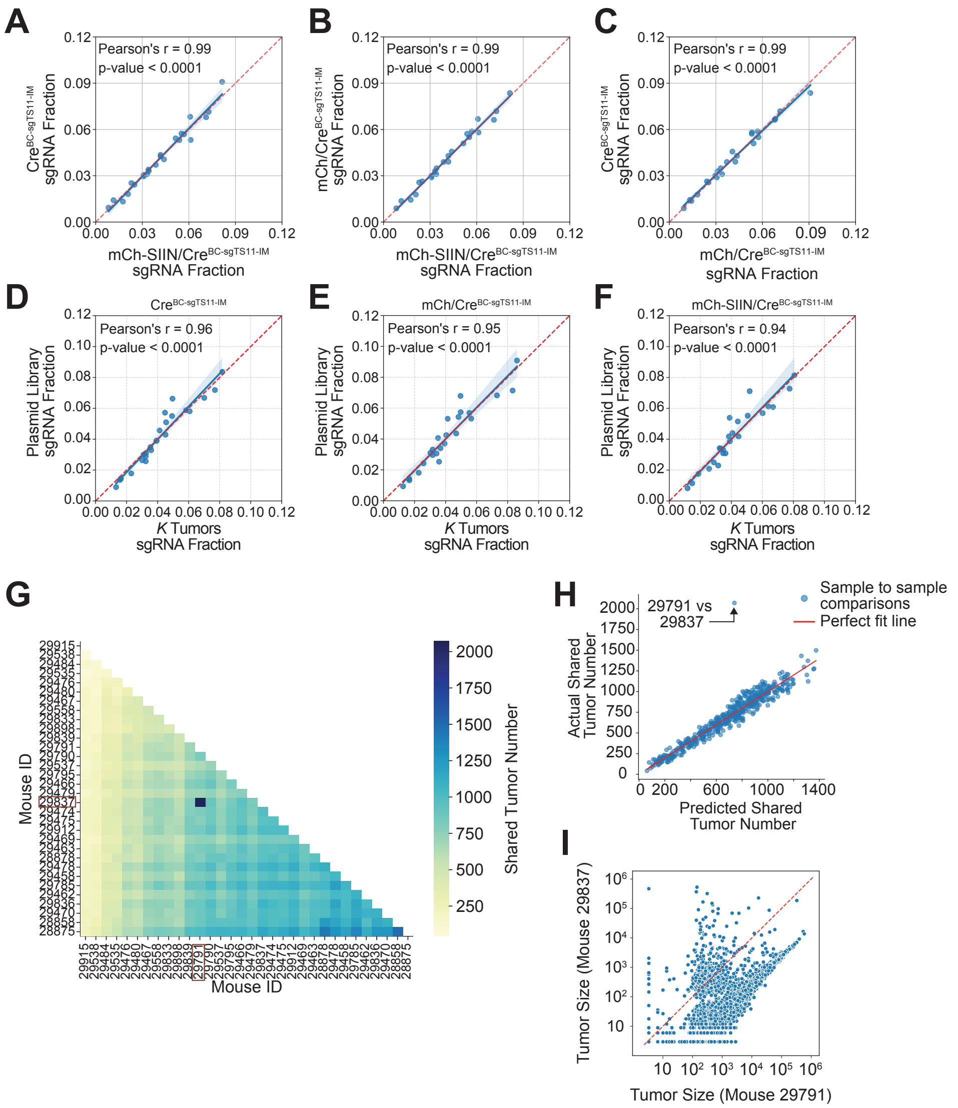

Fig. S10. Assessing the quality of sgRNA representation and library preparation after next generation sequencing. A to C. Correlation of sgRNA read fractions of individual sgRNAs from each plasmid library. Each comparison is indicated in the corresponding graph. Pearson's *r* was calculated as the correlation of sgRNA read fractions between two vectors. **D to F.** Correlation of sgRNA read fractions from plasmid library and *K* tumors initiated with Lenti:Cre$^{BC\text{-}sgTS11\text{-}IM}$ (D), Lenti:mCh/Cre$^{BC\text{-}sgTS11\text{-}IM}$ (E), or Lenti:mCh-SIIN/Cre$^{BC\text{-}sgTS11\text{-}IM}$ (F). Pearson's *r* was calculated as the correlation of sgRNA representation between the plasmid library and K tumors. **G.** Heatmap of shared tumors with the same BC-sgRNA sequence in Cre$^{BC\text{-}sgTS11\text{-}IM}$ tumors. **H.** Plot of actual versus expected shared tumor counts with the same BC-sgRNA sequence from Mouse 29791 and 29837 that identifies a higher number of shared tumors than expected due to random sampling. **I.** Tumor size from Mouse 29791 and 29837 showing a subset of tumors that aligns well with the predicted overlap value due to random sampling and another subset of tumors that indicate contamination.

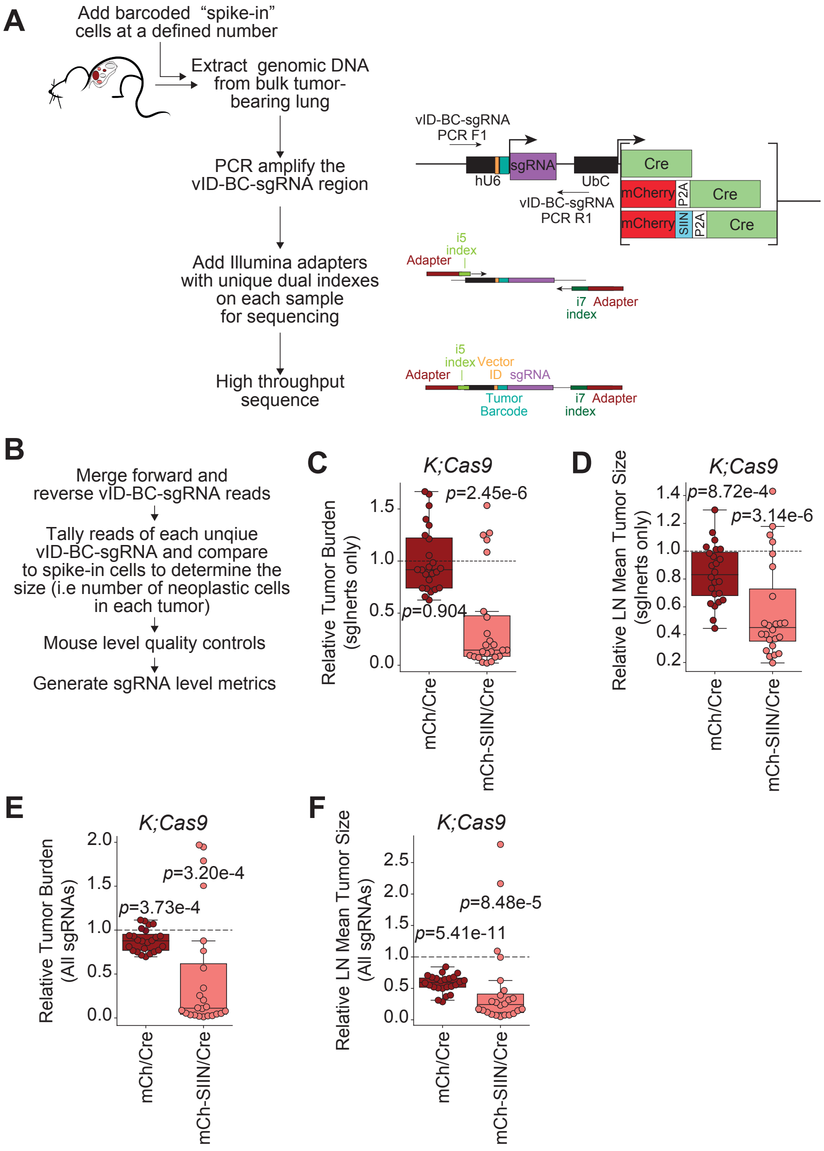

Fig. S11. Tuba-seq library preparation and sequencing analysis reveals increasing neoantigen potency restricts tumor outgrowth. A. Schematic of nested PCR strategy used for Tuba-seq library preparation before next-generation sequencing. **B.** General summary of steps involved in Tuba-seq analysis after next-generation sequencing. See Methods for more details. **C and D.** Tumor burden (E) or LN mean tumor size (F) of mCh/Cre$^{BC\text{-}sgTS11\text{-}IM}$ and mCh-SIIN/Cre$^{BC\text{-}sgTS11\text{-}IM}$ tumors relative to Cre$^{BC\text{-}sgTS11\text{-}IM}$ tumors across *K;Cas9* mice, focusing exclusively on TS^{WT} tumors. Box plots show the median (center line), interquartile range (box; 25th–75th percentiles), and whiskers extending to 1.5x the interquartile range. Individual data points from each mouse are overlaid as dots. One-sample t-tests were used to determine if the estimates were significantly different from 1. **E and F.** Tumor burden (E) or LN mean tumor size (F) of mCh/Cre$^{BC\text{-}sgTS11\text{-}IM}$ and mCh-SIIN/Cre$^{BC\text{-}sgTS11\text{-}IM}$ tumors relative to Cre$^{BC\text{-}sgTS11\text{-}IM}$ tumors in *K;Cas9* mice looking at all sgRNAs except for those targeting *PD-L1* and *B2M*. Box plots show the median (center line), interquartile range (box; 25th–75th percentiles), and whiskers extending to 1.5x the interquartile range. Individual data points from each mouse are overlaid as dots. One-sample t-tests were used to determine if the estimates were significantly different from 1.

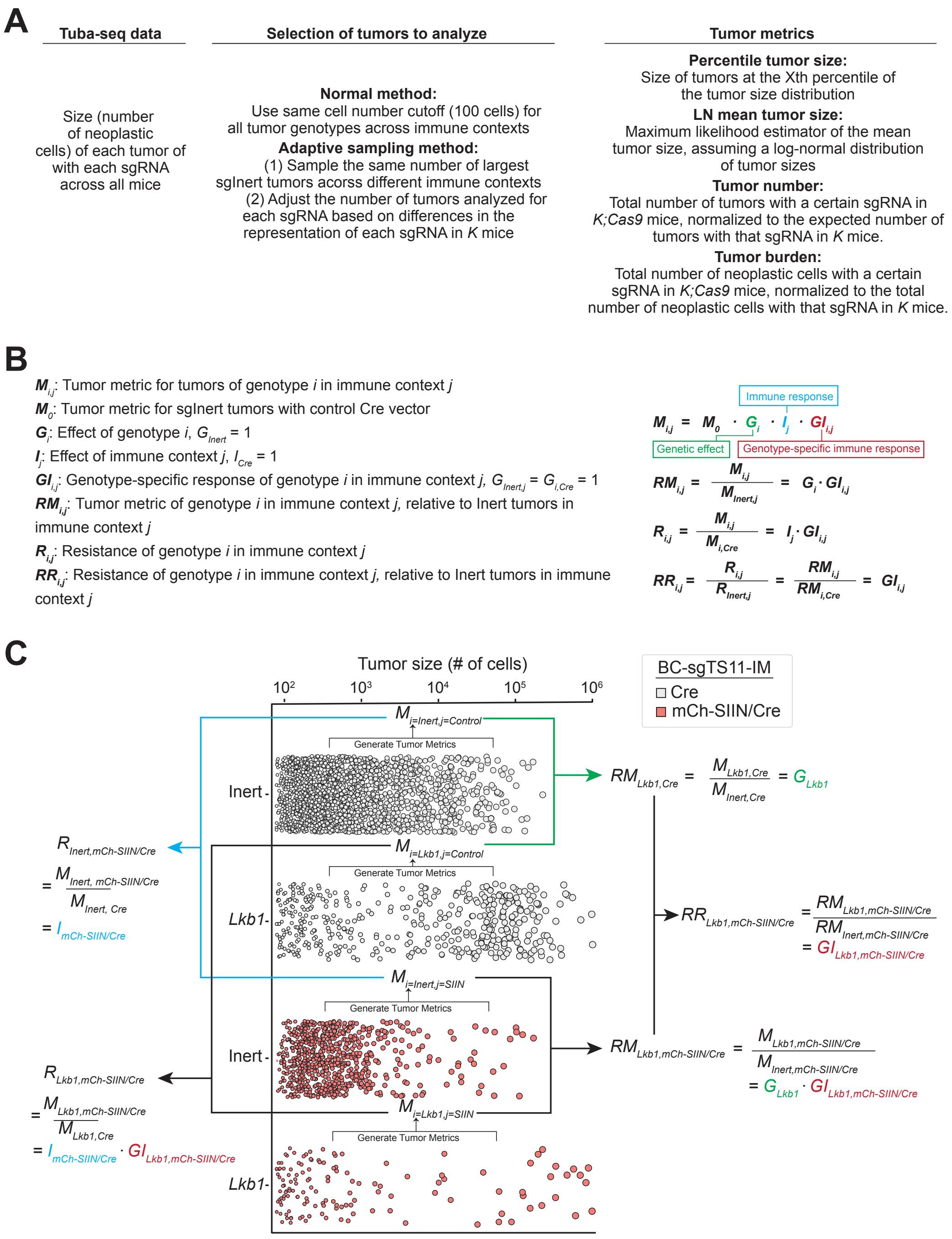

Fig. S12. Model and methodology for disentangling tumor fitness contribution. A. Explanation of methods and metrics used to analyze Tuba-seq data. **B.** Mathematical explanation of analysis methods used to distinguish the effects of tumor suppressor gene inactivation and immunogenic context on overall tumor fitness. **C.** Illustrations demonstrating the application of the mathematical model to dissect the contributions of genetic effects, immune responses, and genotype-specific immune responses to tumor fitness using *Lkb1*^{KO} as an example.

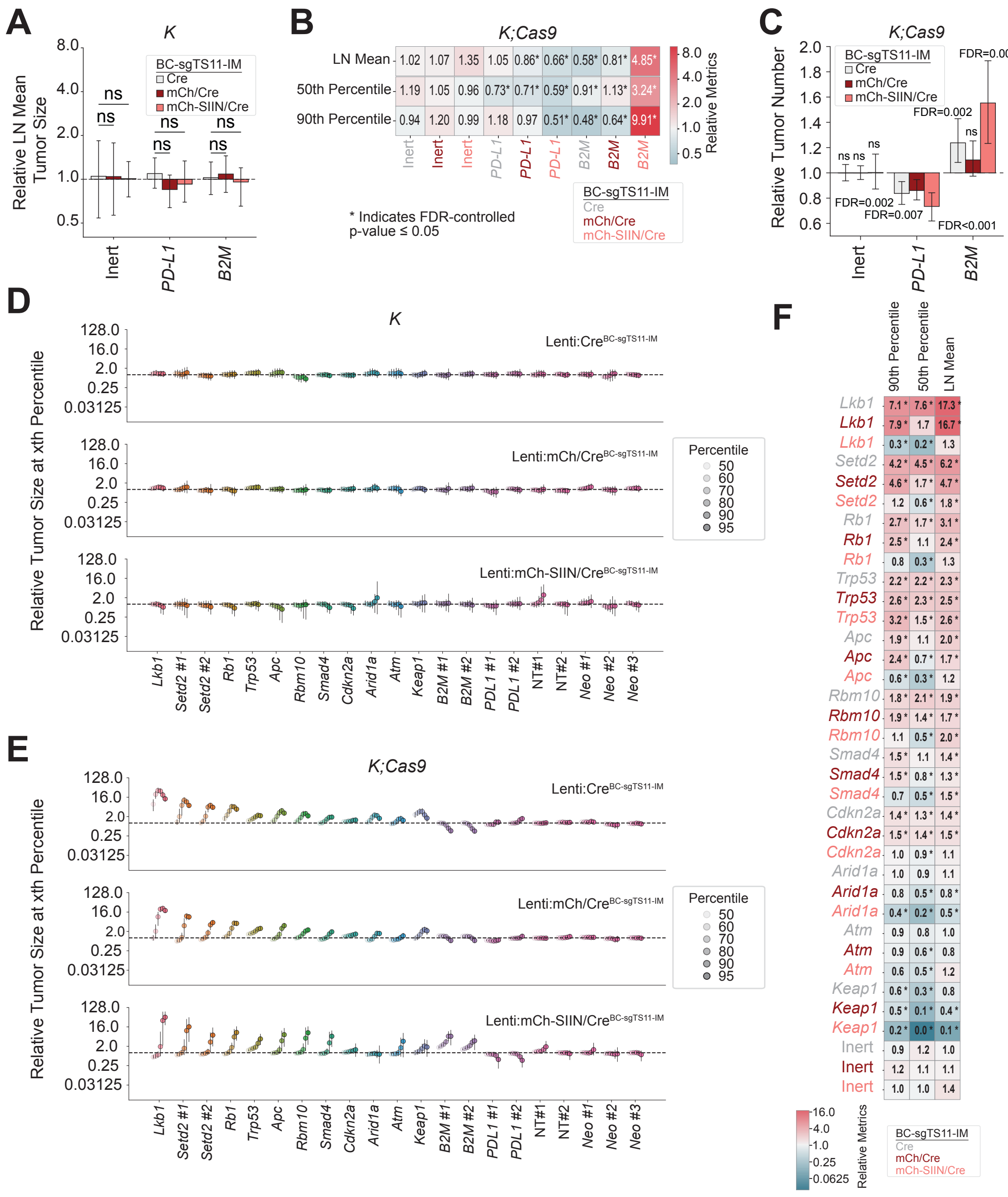

Fig. S13. Immunogenic context shapes the effect of immunomodulatory or tumor suppressor gene inactivation on tumor outgrowth. A. Quantification of relative LN mean tumor size for the TS^{WT,} *PD-L1^{KO}*, and *B2M^{KO}* genotypes initiated with Lenti:Cre$^{BC\text{-}sgTS11\text{-}IM}$, Lenti:mCh/Cre$^{BC\text{-}sgTS11\text{-}IM}$, or Lenti:mCh-SIIN/Cre$^{BC\text{-}sgTS11\text{-}IM}$ in *K* mice. Data are presented as medians with 95% confidence intervals derived from bootstrap resampling (10,000 iterations). Empirical two-sided *p*-values were computed from bootstrap comparisons between groups and adjusted for multiple testing using FDR correction. **B.** Heat map depicting relative tumor metrics for the TS^{WT,} *PD-L1^{KO}*, and *B2M^{KO}* genotypes initiated with Lenti:Cre$^{BC\text{-}sgTS11\text{-}IM}$, Lenti:mCh/Cre$^{BC\text{-}sgTS11\text{-}IM}$, or Lenti:mCh-SIIN/Cre$^{BC\text{-}sgTS11\text{-}IM}$ in *K;Cas9* mice. Raw values are shown in heatmap and asterisk indicates FDR-controlled *p*-value≤ 0.05. **C.** Quantification of relative tumor number for the TS^{WT,} *PD-L1^{KO}*, and *B2M^{KO}* genotypes initiated with Lenti:Cre$^{BC\text{-}sgTS11\text{-}IM}$, Lenti:mCh/Cre$^{BC\text{-}sgTS11\text{-}IM}$, or Lenti:mCh-SIIN/Cre$^{BC\text{-}sgTS11\text{-}IM}$ in *K;Cas9* mice. Data are presented as medians with 95% confidence intervals derived from bootstrap resampling (10,000 iterations). Empirical *P*

values were calculated as the proportion of bootstrap iterations in which relative tumor number exceeded or fell below 1. Two-sided *P* values were obtained by doubling the one-sided values, followed by FDR correction for multiple testing. **D and E.** Relative tumor sizes at the indicated percentiles for each sgRNA in the BC-sgTS11-IM library in *K* (D) or *K;Cas9* (E) mice. Data are presented as medians with 95% confidence intervals derived from bootstrap resampling (10,000 iterations). **F.** Heat map depicting relative metrics in *K;Cas9* mice for tumors initiated with Lenti:Cre$^{BC\text{-}sgTS11\text{-}IM}$, Lenti:mCh/Cre$^{BC\text{-}sgTS11\text{-}IM}$, or Lenti:mCh-SIIN/Cre$^{BC\text{-}sgTS11\text{-}IM}$ for all tumor suppressor genotypes. Raw values are shown in heatmap and asterisk indicates FDR-controlled *p*-value≤ 0.05.

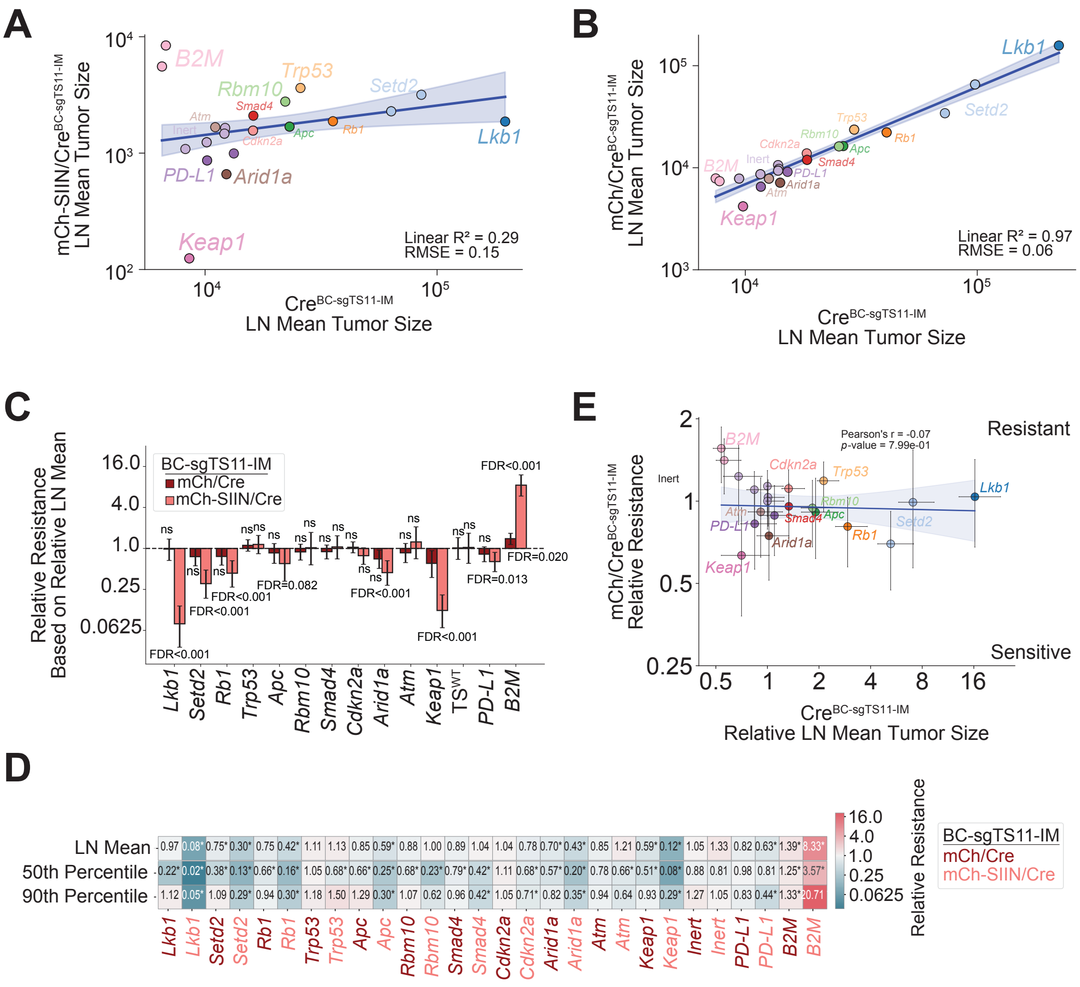

Fig. S14. Tumor suppressor genotype modulates a tumor's susceptibility to immunosurveillance upon potent neoantigen expression. A and B. LN mean tumor size of Cre$^{BC\text{-}sgTS11\text{-}IM}$ tumors versus mCh-SIIN/Cre$^{BC\text{-}sgTS11\text{-}IM}$ (A) or mCh/Cre$^{BC\text{-}sgTS11\text{-}IM}$ (B) tumors with a linear fit. **C.** Relative resistance metric calculated based on LN mean tumor size for tumors initiated by Lenti:mCh/Cre$^{BC\text{-}sgTS11\text{-}IM}$ or Lenti:mCh-SIIN/Cre$^{BC\text{-}sgTS11\text{-}IM}$ across each genotype. Data are presented as medians with 95% confidence intervals derived from bootstrap resampling (10,000 iterations). Empirical *P* values were calculated as the proportion of bootstrap iterations in which relative tumor number exceeded or fell below 1. Two-sided *P* values were obtained by doubling the one-sided values, followed by FDR correction for multiple testing. **D.** Heatmap depiction of relative resistance calculated based on different tumor metrics for tumors initiated by Lenti:mCh/Cre$^{BC\text{-}sgTS11\text{-}IM}$ or Lenti:mCh-SIIN/Cre$^{BC\text{-}sgTS11\text{-}IM}$ across each genotype. Raw values are shown in heatmap and asterisk indicates FDR-controlled *p*-value≤ 0.05. **E.** The relationship between the Relative Resistance metric of mCh-SIIN/Cre$^{BC\text{-}sgTS11\text{-}IM}$ tumors and the LN mean tumor size of Lenti:Cre$^{BC\text{-}sgTS11\text{-}IM}$ tumors for each sgRNA in the BC-sgTS11-IM pool. For each gene, a z-score was computed from the vertical residual relative to a linear regression fit between LN mean tumor size and resistance. Two-sided *p*-values were calculated assuming normally distributed residuals and adjusted for multiple testing using FDR correction. No genes significantly deviated from the fitted relationship (FDR-adjusted *P* value >= 0.1for all dots). Data are shown as medians with 95% confidence intervals from 10,000 bootstrap iterations.
