# Supplementary material for: Tumor suppressor genotype influences the extent and mode of immunosurveillance in lung cancer
Source: Nat Commun. 2026 Jun 15;17:7534. doi: 10.1038/s41467-026-74023-x (PMC13408662; doi:10.1038/s41467-026-74023-x)
Supplement: Supplementary file 2 — Reporting Summary [file 41467_2026_74023_MOESM2_ESM.pdf]

nature portfolio

Corresponding author(s): David Feldser, Monte Winslow

Last updated by author(s): Apr 8, 2026

Reporting Summary

Nature Portfolio wishes to improve the reproducibility of the work that we publish. This form provides structure for consistency and transparency in reporting. For further information on Nature Portfolio policies, see our Editorial Policies and the Editorial Policy Checklist.

Statistics

For all statistical analyses, confirm that the following items are present in the figure legend, table legend, main text, or Methods section.

n/a	Confirmed	
☐	☒	The exact sample size (*n*) for each experimental group/condition, given as a discrete number and unit of measurement
☐	☒	A statement on whether measurements were taken from distinct samples or whether the same sample was measured repeatedly
☐	☒	The statistical test(s) used AND whether they are one- or two-sided<br>*Only common tests should be described solely by name; describe more complex techniques in the Methods section.*
☒	☐	A description of all covariates tested
☐	☒	A description of any assumptions or corrections, such as tests of normality and adjustment for multiple comparisons
☐	☒	A full description of the statistical parameters including central tendency (e.g. means) or other basic estimates (e.g. regression coefficient) AND variation (e.g. standard deviation) or associated estimates of uncertainty (e.g. confidence intervals)
☐	☒	For null hypothesis testing, the test statistic (e.g. *F*, *t*, *r*) with confidence intervals, effect sizes, degrees of freedom and *P* value noted<br>*Give P values as exact values whenever suitable.*
☒	☐	For Bayesian analysis, information on the choice of priors and Markov chain Monte Carlo settings
☒	☐	For hierarchical and complex designs, identification of the appropriate level for tests and full reporting of outcomes
☐	☒	Estimates of effect sizes (e.g. Cohen's *d*, Pearson's *r*), indicating how they were calculated

Our web collection on statistics for biologists contains articles on many of the points above.

Software and code

Policy information about availability of computer code

Data collection	Attune Flow Cytometry Software v5.3.0 was used to collect flow cytometry data.
Data analysis	ImageJ was used for histological assessments and quantifications. Flow cytometry gating was conducted on FlowJo software v10.8.1. Quantitative and statistical analysis of histological and flow cytometric data was conducted using GraphPad Prism 10. Raw sequencing data were processed using AdapterRemoval (v2.3.1) and Bartender (v1.1). Python 3.12 was used for all the downstream analyses and data visualization. Code used in this study is available at GitHub (https://github.com/JasperXuEvolution/UltraSeqImmune) and has been archived at Zenodo (https://doi.org/10.5281/zenodo.19393965).

For manuscripts utilizing custom algorithms or software that are central to the research but not yet described in published literature, software must be made available to editors and reviewers. We strongly encourage code deposition in a community repository (e.g. GitHub). See the Nature Portfolio guidelines for submitting code & software for further information.

Data

Policy information about availability of data

All manuscripts must include a data availability statement. This statement should provide the following information, where applicable:

- Accession codes, unique identifiers, or web links for publicly available datasets
- A description of any restrictions on data availability
- For clinical datasets or third party data, please ensure that the statement adheres to our policy

The sequencing data set generated and analyzed during the current study can be accessed from the NCBI Gene Expression Omnibus database with the following accession number: GSE294730.

Research involving human participants, their data, or biological material

Policy information about studies with human participants or human data. See also policy information about sex, gender (identity/presentation), and sexual orientation and race, ethnicity and racism.

Reporting on sex and gender	Our study did not use human research participants or biological material.
Reporting on race, ethnicity, or other socially relevant groupings	Our study did not use human research participants or biological material.
Population characteristics	Our study did not use human research participants or biological material.
Recruitment	Our study did not use human research participants or biological material.
Ethics oversight	Our study did not use human research participants or biological material.

Note that full information on the approval of the study protocol must also be provided in the manuscript.

Field-specific reporting

Please select the one below that is the best fit for your research. If you are not sure, read the appropriate sections before making your selection.

☒ Life sciences ☐ Behavioural & social sciences ☐ Ecological, evolutionary & environmental sciences

For a reference copy of the document with all sections, see nature.com/documents/nr-reporting-summary-flat.pdf

Life sciences study design

All studies must disclose on these points even when the disclosure is negative.

Sample size	No statistical method was used to predetermine sample size. The size of each animal cohort was determined by estimating biologically relevant effect sizes between control and experimental groups and then using the minimum number of animals that could reveal statistical significance using the indicated tests of significance.
Data exclusions	One sample was discarded due to contaminated sequencing reads from another sample. Another sample was excluded because the Lenti:mCh-SIIN/Cre vector did not reduce tumor burden as expected. Finally, one sample was removed because the knockout effects of canonical genes were inconsistent with other mice of the same genotype based on PCA analysis. These exclusion criteria were pre-established, and their rationale is described in detail in the Methods.
Replication	Animals were enrolled into in vivo experiments as they became available until sufficient numbers were obtained to determine experimental outcomes.
Randomization	Allocation of mice into experimental groups was random.
Blinding	Investigators were blinded to sample identity and group when conducting histological and immunohistochemical analyses.

Reporting for specific materials, systems and methods

We require information from authors about some types of materials, experimental systems and methods used in many studies. Here, indicate whether each material, system or method listed is relevant to your study. If you are not sure if a list item applies to your research, read the appropriate section before selecting a response.

Materials & experimental systems

n/a	Involved in the study	
☐	☒	Antibodies
☐	☒	Eukaryotic cell lines
☒	☐	Palaeontology and archaeology
☐	☒	Animals and other organisms
☒	☐	Clinical data
☒	☐	Dual use research of concern
☒	☐	Plants

Methods

n/a	Involved in the study	
☒	☐	ChIP-seq
☐	☒	Flow cytometry
☒	☐	MRI-based neuroimaging

Antibodies

Antibodies used	Antibodies used for immunohistochemistry:<br>CD45 (1:100, Biolegend, 103102), CD3 (1:700, Abcam, ab5690), mCherry (1:500, Novus Biologicals, NBP2-25156SS), LKB1 (1:250, Cell Signaling, 13031S), RB (1:50, Abcam, ab181616), H3K36me3 (1:2000, Abcam, ab9050), CD8 (1:500, Cell Signaling, 98941S, clone D4W2Z), FoxP3 (1:500, Cell Signaling, 12653S, clone D6O8R), Ly6G (1:500, Biolegend, 127601, clone IA8), and Arginase I (1:1000, Thermo Scientific, PA5-29645).<br><br>Primary antibodies used for flow cytometry:<br>LIVE/DEAD™ Fixable Near IR (Invitrogen, L34992), CD45 (1:200, eFluor 450, Invitrogen, 48-0451-82, clone 30-F11), H2Kb (1:200, BV510, Biolegend, 116523, clone AF6-88.5), H2Kb-SIINFEKL (1:100, APC, Biolegend, 141605, clone 25-D1.16), CD3 (1:100, PE-Cy7, BD Pharmingen, 552774, clone 145-2C11), CD4 (1:200, BV711, Invitrogen, 407-0042-80, clone RM4-5), CD8 (1:200, APC-eFluor 780, Invitrogen, 47-0081-82, clone 53-6.7), CD62-L (1:200, BV480, BD Biosciences, 746726, clone MEL-14), CD44 (1:200, PerCP-Cy5.5, Tonbo Biosciences, 65-0441-UO25, clone IM7), H2Kb-SIINFEKL Tetramer (1:200, Alexa Fluor 647, NIH Tetramer Core Facility), PD-1 (1:200, SuperBright 600, Invitrogen, 63-9985-80, clone J43), and CD39 (1:200, PE, Biolegend, 143803, clone Duha59).
Validation	All antibodies were used in accordance to the manufacturer guidelines and have been well documented in the literature. When possible, antibodies were validated beyond the manufacturers data sheet specification by staining slides by immunohistochemistry with known deficiencies for expression of the antigen.

Eukaryotic cell lines

Policy information about cell lines and Sex and Gender in Research

Cell line source(s)	HEK293FT cells used for lentivirus production were obtained from Invitrogen. GreenGo cells used for titering lentivirus were obtained from Tyler Jack's Laboratory and are a derivative of NIH3T3 cells. The mouse lung adenocarcinoma cell line LG1233 used to generate benchmark cells for Tuba-seq were obtained from Tyler Jack's Laboratory.
Authentication	NIH3T3-GreenGo cells were validated by measuring Cre induced GFP expression. All cell lines were validated with PCR genotyping for expected alleles.
Mycoplasma contamination	All cell lines used in study tested negative for mycoplasma contamination.
Commonly misidentified lines (See ICLAC register)	We have not utilized any misidentified cell lines in our study.

Animals and other research organisms

Policy information about studies involving animals; ARRIVE guidelines recommended for reporting animal research, and Sex and Gender in Research

Laboratory animals	This study utilized laboratory animals of the species mus musculus. All mice utilized are of C57Bl/6J background. Tumors were initiated by 20 weeks of age.
Wild animals	This study did not involve wild animals.
Reporting on sex	In vivo experiments were sex balanced, to the best of our ability.
Field-collected samples	This study did not utilize field-collected samples.
Ethics oversight	Our animal protocol was approved by the University Laboratory Animal Resources (ULAR) at the University of Pennsylvania and the IACUC #804774.

Note that full information on the approval of the study protocol must also be provided in the manuscript.

Plants

Seed stocks

This study did not utilize any seed stocks or plant materials.

Novel plant genotypes

This study did not utilize any seed stocks or plant materials.

Authentication

This study did not utilize any seed stocks or plant materials.

Flow Cytometry

Plots

Confirm that:

☒ The axis labels state the marker and fluorochrome used (e.g. CD4-FITC).

☒ The axis scales are clearly visible. Include numbers along axes only for bottom left plot of group (a 'group' is an analysis of identical markers).

☐ All plots are contour plots with outliers or pseudocolor plots.

☒ A numerical value for number of cells or percentage (with statistics) is provided.

Methodology

Sample preparation

Lungs were harvested from mice and 1-3 lobes per mouse were set aside for flow cytometry. For flow cytometry on tumor cells, lung tissue was cut into small pieces and 1mL of digestion buffer was added to each sample. Each 1mL of digestion buffer contained 700uL HBSS media (-Mg2+ -Ca2+), 100 uL Trypsin-EDTA (0.25%), 100uL Collagenase IV (Worthington Biochemical Corp., LS004188, from 10 mg/mL stock dissolved in HBSS), 0.5U Dispase (Corning, 354235, 5U/mL stock). Samples were shaken at 37ºC for 45 min at 200 rpm to digest the tissue. Tissue was further dissociated using a p200 pipette. 2mL of quench buffer was added to each sample to halt tissue digestion. Each 2mL of Quench buffer contained 200 uL fetal bovine serum and 7.5 uL DNase (Roche Diagnostics, 10104159001, from 10 mg/mL stock dissolved in water) diluted in 1.8 mL Leibovitz's L-15 media (Gibco, 11415064). For immunophenotyping of T cells, lung tissue was digested in a gentleMACS C tube (Miltenyi Biotec, 130-093-237) using the gentleMACS Octo Dissociator. Each 1mL of digestion buffer contained 5uL fetal bovine serum, 78uL Collagenase IV (Worthington Biochemical Corp., LS004188, from 10mg/mL stock dissolved in HBSS), and 4uL DNase (Roche Diagnostics, 10104159001, from 10mg/mL stock dissolved in water) dissolved in 913uL HBSS. Each sample received 3.5 mL of digestion buffer, and double the volume of quench buffer (RPMI with 2% FBS) was used to stop the digestion.

All samples were then passed through a 45um filter and red blood cells were lysed with 1 mL ACK lysis buffer (Gibco, A1049201) for 5 min. The lysis reaction was then quenched with 9 mL PBS. Samples were then stained a cocktail of primary antibodies and viability dye for in the dark 30 min at 4ºC. LIVE/DEAD™ Fixable Olive (Invitrogen, L34977) or LIVE/DEAD™ Fixable Near IR (Invitrogen, L34992) were used as directed by the manufacturer. The antibodies used were: CD45 (1:200, eFluor 450, Invitrogen, 48-0451-82, clone 30-F11), H2Kb (1:200, BV510, Biolegend, 116523, clone AF6-88.5), H2Kb-SIINFEKL (1:100, APC, Biolegend, 141605, clone 25-D1.16), CD3 (1:100, PE-Cy7, BD Pharmingen, 552774, clone 145-2C11), CD4 (1:200, BV711, Invitrogen, 407-0042-80, clone RM4-5), CD8 (1:200, APC-eFluor 780, Invitrogen, 47-0081-82, clone 53-6.7), CD62-L (1:200, BV480, BD Biosciences, 746726, clone MEL-14), CD44 (1:200, PerCP-Cy5.5, Tonbo Biosciences, 65-0441-UO25, clone IM7), H2Kb-SIINFEKL Tetramer (1:200, Alexa Fluor 647, NIH Tetramer Core Facility), PD-1 (1:200, SuperBright 600, Invitrogen, 63-9985-80, clone J43), and CD39 (1:200, PE, Biolegend, 143803, clone Duha59).

Cells were then washed twice with FACS buffer. Finally, cells were resuspended in FACS buffer and flow cytometry was performed using an Attune NxT flow cytometer (Thermo Fisher). Data were analyzed in FlowJo. Tumor cells were gated on CD45NegativeGFPPositive live singlets (Fig. S3). Gating for T cell immunophenotyping is shown in Fig. S6.

Instrument

The Thermo Scientific Attune NxT, Acoustic Focusing Cytometer, Model AFC2 was used for all flow cytometry experiments

Software

Attune Flow Cytometry Software was used to collect flow cytometry data. Data were analyzed using FlowJo software v10.8.1.

Cell population abundance

Reported as the percentage of total events after gating out debris.

Gating strategy

Debris was removed from the analysis using a FSC-A vs SSC-A plot. Then, singlets were selected using a FSC-A vs FSC-H plot. Then, live cells were selected by gating cells that were negative for LIVE/DEAD™ Fixable Near IR (Invitrogen, L34992) using SSC-A as the Y-axis.

Tumor and immune cell populations for in vivo experiments were defined as follows:
1. Tumor cells: CD45-GFP+

2. T cells: CD45+CD3+
3. CD8+ T cells: CD45+CD3+CD8+
4. CD4+ T cells: CD45+CD3+CD4+
5. Naïve T cells: CD45+CD3+ (CD4+ or CD8+)CD44-CD62L+
6. Central Memory T cells: CD45+CD3+ (CD4+ or CD8+)CD44+CD62L+
7. Effector T cells: CD45+CD3+ (CD4+ or CD8+)CD44+CD62L-
8. SIIN-specific T cells: CD45+CD3+ (CD4+ or CD8+)H2Kb-SIINFEKL Tetramer+
9. Exhausted T cells: CD45+CD3+ (CD4+ or CD8+) (CD39+ and/or PD-1+)

☒ Tick this box to confirm that a figure exemplifying the gating strategy is provided in the Supplementary Information.
