# Supplementary material for: Tumor suppressor genotype influences the extent and mode of immunosurveillance in lung cancer
Source: Nat Commun. 2026 Jun 15;17:7534. doi: 10.1038/s41467-026-74023-x (PMC13408662; doi:10.1038/s41467-026-74023-x)
Supplement: Supplementary file 3 — Transparent Peer Review file [file 41467_2026_74023_MOESM3_ESM.pdf]

nature portfolio

Peer Review File

Tumor suppressor genotype influences the extent and mode of immunosurveillance in lung cancer

Corresponding Author: Dr David Feldser

This file contains all reviewer reports in order by version, followed by all author rebuttals in order by version.

Version 0:

Reviewer comments:

Reviewer #1

(Remarks to the Author)
In this report, Adler et al. engineer several mouse models based on a combination of KrasG12D knock-in, Lenti:Cre, Lenti:mCh/Cre, or Lenti:mCh/SIIN/Cre (where SIIN is a highly immunogenic MHC-1 pepetide) to evaluate the impact of 14 tumor suppressor genes, B2M, and PDL1 on immunoediting and immunosurveillance. The authors first compare the effects of the TSGs alone in each immune context where Cre is the least immunogenic and the SIIN construct is the most immunogenic. The authors show that each TSG has a variable effect on immunoediting depending on which immunogenic background was used. Tumors harboring inactivation of Lkb1, Setd2, or Rb1 were each significantly immunoedited in highly immunogenic conditions, but that each escaped immune pressures through distinct mechanisms that were related to differential regulation of neoantigen expression and recruitment of different immune cell subtypes. In addition, the authors employed a tumor barcoding strategy to simultaneously assess the effect of the TSGs across the 3 levels of immunogenicity in parallel.

The authors found a strong positive relationship between the rate of tumor growth after inactivation of specific tumor suppressor genes in poorly immunogenic tumors and the sensitivity of genotypically identical tumors to immunosurveillance in highly immunogenic contexts. The authors show that inactivation of Keap1 or Arid1a increased the tumor's susceptibility to immunosurveillance despite having only small pro-growth effects on tumor outgrowth in poorly immunogenic contexts. Whereas the authors show that inactivation of Trp53 or Rbm10 limited immunosurveillance regardless of immunogenic context.

Overall, this manuscript is well written, and the author's conclusions are supported by the data shown.

Since the primary purpose of this manuscript is to establish a mouse model of Kras driven lung cancer, tumor suppressor gene inactivation and their interactions with the immune system to share with the community, the lack of mechanistic insight into the tumor intrinsic effects of TSG inactivation and the microenvironment is probably ok. I look forward to seeing further characterization of this model in the future. It will also be interesting to see if this model can be used to better understand how the immune system works during early tumorigenesis.

I have only a minor comment:

There are a few grammatical errors throughout.

Reviewer #2

(Remarks to the Author)
I co-reviewed this manuscript with one of the reviewers who provided the listed reports. This is part of the Nature Communications initiative to facilitate training in peer review and to provide appropriate recognition for Early Career Researchers who co-review manuscripts.

Reviewer #3

(Remarks to the Author)
In this manuscript, Adler et al present a well-designed study addressing the role of tumor suppressor gene inactivation in shaping immunoediting during early lung tumorigenesis. Using a combination of neoantigen-expressing lenti vectors, CRISPR-based somatic gene editing, and Tuba-seq tumor barcoding within autochthonous mouse models, the authors examine how tumor genotype influences immune surveillance. The technically-savvy study presents a valuable experimental platform and provides highly relevant insight into tumor-immune interactions.

The strengths of the manuscript include:

Development of a lenti vector system that allows neoantigen expression linked to a fluorescent reporter (mCherry-SIIN) that enables tumor dynamics tracking in the context of CRISPR based gene editing of the tumors and Tuba-seq

High-throughput analysis of 11 tumor suppressor gene inactivations

Integration of Tuba-seq to examine tumor growth, Ag silencing, and immune pressure in vivo

The key finding—that the inactivation of certain tumor suppressor genes either sensitizes tumors to, or protects them from, immunoediting—is an important contribution. Particularly notable is the observation that tumor suppressors such as Keap1 and Arid1a, which confer only mild growth advantages in poorly immunogenic settings, nonetheless significantly increase susceptibility to immune surveillance when strong neoantigens are expressed. Conversely, Trp53 and Rbm10 inactivation limit immunoediting, consistent with emerging literature suggesting their role in immune escape (e.g., PMIDs 32312757).

However, the study also has critical limitations, which should be addressed in the interest of strengthening the manuscript:

Major Concern:

The central claim of the study is that tumor suppressor genotype shapes immunoediting and immune escape. However, the mechanisms underlying these effects are not defined, nor are the specific immune cells responsible for the observed outcomes identified.
For example, while the authors show that Lkb1-deficient tumors more frequently downregulate neoantigen expression, and that Setd2 or Rb1-deficient tumors do not, there is not insight into which immune cells drive this selection pressure, or whether differences in infiltration (e.g., CD8+ vs. Treg vs. MDSC) are causal or correlative. The authors conclude that distinct mechanisms of immune evasion are used across genotypes. While it is fair to say that precise mechanisms may be outside of the scope of the current study, I would suggest that at minimum CD8 depletion or pan-T depletion (or depletion of NK cells, etc) studies can be performed for a focused group of genotypes to clearly demonstrate role of well-established immune subsets in immune editing and the critical timing of immune responses in the dynamics of tumor growth. Showing that T cells play a role in immunoediting in some of the genotypes presented, but not others would significantly strengthen the author's argument that pathways of immune evasion are distinct between the genotypes presented.
Additional mechanistic insight (assessment of T cell exhaustion in tumors with retained antigen, staining for proliferation/apoptosis, etc) would also be valuable across the genotypes described.

Other Suggestions/concerns:

1) The authors should better contextualize some findings that differ from established associations in human tumors. For instance:
-Keap1 loss is typically associated with immune evasion in NSCLC (PMIDs 32312757, 37889752), yet here, Keap1KO tumors appear more immunogenic. This may be a question of timing of Keap1 loss, etc – but at minimum this should be discussed?
-Setd2 mutation is reported to be associated with immunotherapy response (PMID 39506116), suggesting Set2d mutation improves anti-tumor immune responses but this is not observed in Fig 6B.
-Lkb1 inactivation has been linked to neutrophil infiltration (PMID 26833127), but the data presented in Fig. 4A and 4B seem to suggest the opposite

2) Tuba-seq presents an exciting opportunity to simultaneously evaluate immune evasion of multiple tumor genotypes. However, it appears that antigenicity of individual tumors may be directly impacted by active immune responses against others – a particular concern when they share neoatigens (such as SIIN or Cas9). This is briefly addressed by the authors in comparing results in this experiment to those of "one-at-a-time" KOs of Lkb1/Setd2 – but seems like a more in depth discussion and greater analysis of TME is warranted.

3) The observation that Lkb1KO tumors exhibit extensive neoantigen silencing but relatively low tumor burden (Fig. 3D, 3F) is intriguing. One possibility is that these tumors experience intense immune pressure early and only a small fraction survive, perhaps with even small number of edited clones facing residual suppression from the immune system. It would be helpful if the authors could provide additional data (later timepoints or immune profiling of edited tumors) to clarify.
Given the availability of a previously published reversible Lkb1 knockout system (PMCID: PMC8885825) that the authors established, the team could also explore whether restoration of Lkb1 can reverse immune editing or if neoantigen loss is fixed. While this experiment may be beyond the scope of the current study, a brief discussion of this, and what the expeted outcome may imply for immune escape, would be welcome.

Reviewer #4

(Remarks to the Author)
In this manuscript, Adler and colleagues investigate how cancer driver mutations shape immunosurveillance during KRAS-driven lung tumorigenesis. By combining a set of increasingly immunogenic lentiviral vectors with CRISPR screening, the authors document how tumor suppressor gene inactivation regulates the level of immunosurveillance in emerging tumors. The authors show that, depending on tumor suppressor genotype, cancer cells use different strategies to escape the immune response and find that faster growing cancers are generally more susceptible to immunosurveillance. Overall, the manuscript is well-written and offers an interesting screening approach for the field to uncover new insights into how tumor genotypes influence cancer progression and immune evasion. However, a number of key controls are lacking and a few conclusions are overstated. The following points should be addressed prior to publication.

Major points:

1. The authors do not provide a time point zero control after lentiviral infection of the mouse lung (Figures 1 & 3). The initial infection rate in the mouse lung should be assessed to allow a more meaningful comparison of tumor burden between different conditions (equal virus titer does not necessarily mean equal infection rates).

2. Regarding the setup of their system, it seems the tumor burden is equal for all 3 vectors between weeks 8-12, only the Cre vector at week 16 is significantly different (Figure 1D). Given the n=4 and because this is an important feature in their system, do the authors have additional evidence that these vectors indeed result in differences in tumor growth (e.g. later time point)? Can the authors also quantify the number of tumors per lung, instead of only tumor burden? How many of these tumors are indeed malignant vs. adenomas, as assessed by pathology? Although the authors claim that tumors increase/do not increase from 8 to 16 weeks, no statistical test has been performed to compare 8 vs. 12 vs. 16 weeks.

3. The authors make the claim that there is a difference in the propensity to select for mCherry-SIIN silencing between Lkb1KO, Setd2KO, or Rb1KO tumors. Although the phenotype seems convincingly strong in Figure 3F, these claims are based on a single sgRNA for each tumor suppressor. The authors validate the on-target effect of these sgRNAs by IHC and point to previously published work, however, this does not confirm specificity. A second sgRNA should be used for Lkb1 KO, Setd2 KO, and Rb1 KO to further ensure that the strong difference in phenotype is not due to an unspecific off-target effect of the sgRNA.

4. Similar to point 1, the authors should provide data on the library representation at the beginning of the CRISPR screen in Figures 5 & 6.

5. Figure 6C/D: An important conclusion the authors reach in this paper is the correlation between strong growth promotion and sensitivity to immunosurveillance with different tumor suppressor genotypes. Considering Figure 6C and S9A, one might draw the alternative conclusion that highly immunogenic conditions set a ceiling/maximum for tumor size that is independent of tumor suppressor genotype. This leads to the same observation, as tumors that grow faster in the absence of this ceiling would have “relative higher sensitivity”, although the ceiling is the same for all TSGs. The authors should provide further evidence that supports their hypothesis or discuss this alternative in the text. In addition, the conclusions are again founded on the use of a single sgRNA per gene in the screening experiment, while conventional CRISPR screens are usually done with 3-5 sgRNAs for several reasons, including ruling out off-target effects.

6. Based on Figure 6B, the authors claim that only inactivation of Trp53, Rbm10 and Smad4 promoted resistance to tumor immunosurveillance. To strengthen this claim, the authors should carry out statistical testing for the comparisons in Figures 6A-B. For example, the Apc KO tumor size seems highly variable in the mCherry-SIIN/Cre condition.

Minor points:

1. It is not mentioned which statistical test was used in Figure 1F. In addition, Figure 1F and Figure 3F suggest different percentages of mCherry negative tumors in the mCh-SIIN/Cre control group: 1F states 60 % mCherry negative tumors at week 12, in 3F it is 40 %. The authors should comment on this.

2. The authors have used student’s t-test to compare across groups (ie. Fig. 1D, 2C, 3D). Since they are often comparing more than 2 groups, was a multiple comparison correction used? Please also indicate the type of correction.

3. Figure 2C/D: As inferred from Figure 1C, there is a considerable difference in tumor burden between the different groups at week 16. It is conceivable that this difference in absolute tumor burden in the lung also influences the immune cell infiltration in a way not directly related to the vectors. The authors should comment if the comparison of immune cell infiltration at this timepoint can be used to compare the immunogenicity of the vectors.

4. Lines 113-117: there’s an increase in the number of immune cells for Lenti::mCh/Cre and Lenti::mCh-SIIN/Cre relative to Lenti::Cre. If the authors want to claim an increase in immune infiltration over time (as described in Line 116), they should

compare across weeks, not between conditions.

5. Line 140: “Unexpectedly, Lkb1KO, Setd2KO or Rb1KO did not significantly increase tumor burden compared to TSWT tumors when initiated with Lenti:mCh-SIIN/Cre.” In the corresponding Figure 3D, no statistical test was used to determine statistical significance between the tumor suppressors and TSWT in mCherry-SIIN tumors.

6. Figures 5B and 5F could be combined into one to enhance clarity for the reader.

7. It would be interesting to show if the relative resistance trend described in Figure 6 also applies to tumor initiation frequency/tumor burden, as supposed to LN tumor size. The total number of tumors for each sgRNA can be inferred from the tumor barcodes.

8. Figure S7 is not very intuitive to understand and has a few typos (“resistence” instead of “resistance”)

9. A period is missing in line 358.

10. “That we see major differences between genotypes and between different immunogenic contexts all within the same organ environment indicates that significant cancer cell-autonomous differences exist that are driven by biological effects of tumor genotype.” Please rephrase.

11. In the discussion, it may be worth mentioning the limitations of using extrinsically introduced immunogenic genes such as SIINFEKL and their biological relevance in a more “natural” setting in terms of tumor formation.

Version 1:

Reviewer comments:

Reviewer #2

(Remarks to the Author)
I co-reviewed this manuscript with one of the reviewers who provided the listed reports. This is part of the Nature Communications initiative to facilitate training in peer review and to provide appropriate recognition for Early Career Researchers who co-review manuscripts.

Reviewer #3

(Remarks to the Author)
I appreciate the thoughtful revisions and the substantial effort made to address the reviewers' suggestions and concerns, which have strengthened the manuscript. My only minor comment is that there appears to be a truncated sentence around lines 367–368, please consider revising for the final version.

Reviewer #4

(Remarks to the Author)
The authors have satisfactorily addressed most of our concerns. The following remaining points should be considered for the final manuscript.

1. Regarding answer 4.2.: It seems that the text has not been updated correctly. The statement “As expected, tumors initiated with Lenti:Cre increased in size over time (Fig. 1, C and D). In contrast, tumors initiated with either Lenti:mCh/Cre or Lenti:mCh-SIIN/Cre did not increase in size from 8 to 16 weeks” (lines 96–98) does not appear to correspond to Figure 1D. Based on Figure 1D, mCh-SIIN/Cre tumors show a statistically significant increase in tumor area at 16 weeks. The text should be updated to reflect the data presented in the figure.

2. The response regarding the use of only a single sgRNA is not convincing and several of the cited studies do also not include validation with multiple sgRNAs, limiting the support for this claim. While we recognize that experimentally addressing this concern at this point would require additional work, including the repetition of multiple experiments, we think that this limitation should be explicitly acknowledged in the Discussion section.

3. The statement “In TSWT and Lkb1KO tumors, mCherry-SIINFEKL expression reduced tumor area” (Lines 149–150) may be misleading, as Figure 3D does not show a statistically significant decrease. We recommend revising the wording to more accurately reflect the data.

4. We appreciate the authors’ effort to address the point 4.6. through the statistical analysis presented in Figure 6D. However, our concern relates specifically to the individual genes shown in Figures 6A and 6B. For example, Apc relative LN mean tumor size Cre vs. mChSIIN/Cre. For full transparency, statistical comparisons between the relevant groups within

each gene should be provided for Figure 6A/B. Conducting analysis only at the level of relative resistance (as in Figure 6D, comparing across all genes) does not fully resolve this point.

Open Access This Peer Review File is licensed under a Creative Commons Attribution 4.0 International License, which permits use, sharing, adaptation, distribution and reproduction in any medium or format, as long as you give appropriate credit to the original author(s) and the source, provide a link to the Creative Commons license, and indicate if changes were made.
In cases where reviewers are anonymous, credit should be given to 'Anonymous Referee' and the source.
The images or other third party material in this Peer Review File are included in the article's Creative Commons license, unless indicated otherwise in a credit line to the material. If material is not included in the article's Creative Commons license and your intended use is not permitted by statutory regulation or exceeds the permitted use, you will need to obtain permission directly from the copyright holder.
To view a copy of this license, visit https://creativecommons.org/licenses/by/4.0/

REVIEWER COMMENTS

REVIEWER 1

"In this report, Adler et al. engineer several mouse models based on a combination of KrasG12D knock-in, Lenti:Cre, Lenti:mCh/Cre, or Lenti:mCh/SIIN/Cre (where SIIN is a highly immunogenic MHC-1 pepetide) to evaluate the impact of 14 tumor suppressor genes, B2M, and PDL1 on immunoediting and immunosurveillance. The authors first compare the effects of the TSGs alone in each immune context where Cre is the least immunogenic and the SIIN construct is the most immunogenic. The authors show that each TSG has a variable effect on immunoediting depending on which immunogenic background was used. Tumors harboring inactivation of Lkb1, Setd2, or Rb1 were each significantly immunoedited in highly immunogenic conditions, but that each escaped immune pressures through distinct mechanisms that were related to differential regulation of neoantigen expression and recruitment of different immune cell subtypes. In addition, the authors employed a tumor barcoding strategy to simultaneously assess the effect of the TSGs across the 3 levels of immunogenicity in parallel.

The authors found a strong positive relationship between the rate of tumor growth after inactivation of specific tumor suppressor genes in poorly immunogenic tumors and the sensitivity of genotypically identical tumors to immunosurveillance in highly immunogenic contexts. The authors show that inactivation of Keap1 or Arid1a increased the tumor's susceptibility to immunosurveillance despite having only small pro-growth effects on tumor outgrowth in poorly immunogenic contexts. Whereas the authors show that inactivation of Trp53 or Rbm10 limited immunosurveillance regardless of immunogenic context.

Overall, this manuscript is well written, and the author's conclusions are supported by the data shown.

Since the primary purpose of this manuscript is to establish a mouse model of Kras driven lung cancer, tumor suppressor gene inactivation and their interactions with the immune system to share with the community, the lack of mechanistic insight into the tumor intrinsic effects of TSG inactivation and the microenvironment is probably ok. I look forward to seeing further characterization of this model in the future. It will also be interesting to see if this model can be used to better understand how the immune system works during early tumorigenesis."

> We thank the Reviewer for their enthusiasm regarding our manuscript and agree that mechanistic insight will be best addressed in a subsequent manuscript, given the novelty of the system used and the discoveries. While no additional scientific experimentation was suggested, we hope the Reviewer agrees that the substantial additions that we have made based on the other Reviewer's comments further bolstered our conclusions and increased the manuscript's impact.

Minor points

MP1.1: "There are a few grammatical errors throughout."

> **A1.1:** We have carefully reviewed the manuscript and corrected all grammatical errors that we have identified.

REVIEWER 2

"I co-reviewed this manuscript with one of the Reviewers who provided the listed reports. This is part of the Nature Communications initiative to facilitate training in peer review and to provide appropriate recognition for Early Career Researchers who co-review manuscripts.

Thank you to the Reviewer for their contribution.

REVIEWER 3

"In this manuscript, Adler et al present a well-designed study addressing the role of tumor suppressor gene inactivation in shaping immunoediting during early lung tumorigenesis. Using a combination of neoantigen-expressing lenti vectors, CRISPR-based somatic gene editing, and Tuba-seq tumor barcoding within autochthonous mouse models, the authors examine how tumor genotype influences immune surveillance. The technically-savvy study presents a valuable experimental platform and provides highly relevant insight into tumor-immune interactions.

The strengths of the manuscript include:

Development of a lenti vector system that allows neoantigen expression linked to a fluorescent reporter (mCherry-SIIN) that enables tumor dynamics tracking in the context of CRISPR based gene editing of the tumors and Tuba-seq

High-throughput analysis of 11 tumor suppressor gene inactivations

Integration of Tuba-seq to examine tumor growth, Ag silencing, and immune pressure in vivo

The key finding—that the inactivation of certain tumor suppressor genes either sensitizes tumors to, or protects them from, immunoediting—is an important contribution. Particularly notable is the observation that tumor suppressors such as Keap1 and Arid1a, which confer only mild growth advantages in poorly immunogenic settings, nonetheless significantly increase susceptibility to immune surveillance when strong neoantigens are expressed. Conversely, Trp53 and Rbm10 inactivation limit immunoediting, consistent with emerging literature suggesting their role in immune escape (e.g., PMIDs 32312757)."

Many thanks to the Reviewer. We agree that the findings from our study provide important insights to the field of tumor immunoediting by highlighting the important, yet under-appreciated role that cancer driver mutations play in this process.

Major points

Q3.1: "The central claim of the study is that tumor suppressor genotype shapes immunoediting and immune escape. However, the mechanisms underlying these effects are not defined, nor are the specific immune cells responsible for the observed outcomes identified. For example, while the authors show that Lkb1-deficient tumors more frequently downregulate neoantigen expression, and that Setd2 or Rb1-deficient tumors do not, there is not insight into which immune cells drive this selection pressure, or whether differences in infiltration (e.g., CD8+ vs. Treg vs. MDSC) are causal or correlative. The authors conclude that distinct mechanisms of immune evasion are used across genotypes. While it is fair to say that precise mechanisms may be outside of the scope of the current study, I would suggest that at minimum CD8 depletion or pan-T depletion (or depletion of NK cells, etc) studies can be performed for a focused group of genotypes to clearly demonstrate role of well-established immune subsets in immune editing and the critical timing of immune responses in the dynamics of tumor growth. Showing that T cells play a role in immunoediting in some of the genotypes presented, but not others would significantly strengthen the author's argument that pathways of immune evasion are distinct between the genotypes presented. Additional mechanistic insight (assessment of T cell exhaustion in tumors with retained antigen, staining for proliferation/apoptosis, etc) would also be valuable across the genotypes described."

A3.1: We thank the Reviewer for this suggestion and have added new experimental analyses to address this. Our original data demonstrated that tumors with different tumor suppressor genotypes distinctly

tolerate neoantigen expression and differentially regulate the recruitment and engagement of the immune response despite having the same neoantigen (mCherry-SIINFEKL). The difference between genotypes was exacerbated by the expression of the MHC-I restricted peptide SIINFEKL, as tumors with weaker neoantigen expression (mCherry) had less distinctive immune evasive phenotypes. While this certainly hints at distinct mechanisms regulating T cell responses, as the Reviewer suggests it would be important to gain additional insight here. However, we also strongly agree with the Reviewer that precise mechanistic understanding is beyond the scope of this study and should be the topic of future work.

To further parse the differences in T cell responses across genetic contexts, we repeated the experiment shown in **Fig. 3 and 4** with a sample size of n=6 per group and immunophenotyped SIINFEKL-specific T cell responses in tumor-bearing lungs across each genotype of interest. We first assessed the effects of tumor suppressor gene inactivation on immune phenotypes when tumors were initiated by Lenti:mCh/Cre^{sgRNA} vectors. Interestingly, we found that *Lkb1* inactivation limited the frequency of both $CD8^{Positive}$ and $CD4^{Positive}$ central memory T (T_{CM}) cells but had little impact on the effector T (T_{EFF}) cell and naïve T (T_N) cell populations (**Fig. S8, A and B**). *Lkb1* inactivation also had a mild effect on T_{CM} cell exhaustion, with $CD8^{Positive}$ T_{CM} cells expressing lower levels of PD-1, and a larger subset of $CD4^{Positive}$ T_{CM} cells expressing both PD-1 and CD39 (**Fig. S8, C to D**). Neither *Setd2* nor *Rb1* inactivation generated any significant differences across T cell populations (**Fig. S8**).

To directly assess SIINFEKL-specific immune response across tumor genotypes, we assessed T_{EFF} cells that had a SIINFEKL-specific TCR using an H2Kb-SIINFEKL tetramer. Interestingly, the frequency of SIINFEKL-specific T_{EFF} cells was two-fold higher in TS^{WT} and *Lkb1*^{KO} tumor-bearing lungs than in *Setd2*^{KO} or *Rb1*^{KO} tumor-bearing lungs (**Fig. 4D**). This could indicate that *Setd2* or *Rb1* inactivation drives immune evasion through a mechanism that limits the ability for T cells to detect and respond to mCherry-SIINFEKL, thus partially explaining the ability for neoantigen expression to be maintained in these genotypes. Importantly, SIINFEKL-specific $CD8^{Positive}$ T_{EFF} cells were similarly exhausted across all tumor genotypes, and SIINFEKL-specific T_{EFF} cells were significantly more exhausted than SIINFEKL-nonspecific T_{EFF} cells, demonstrating prior antigen stimulation and exhaustion response across all genotypes (**Fig. 4E**). This strongly suggests engagement of T_{EFF} cells with SIINFEKL across all genotypes, despite our observations that mCherry-SIINFEKL expression is severely diminished in *Lkb1*^{KO} tumors relative to TS^{WT} tumors, but not *Setd2*^{KO} or *Rb1*^{KO} tumors. We believe this is exactly the insight that the Reviewer is looking for—**while SIINFEKL elicits an immune response across all tumor suppressor genotypes, tumors of specific genotypes had distinct levels and modes of immunoediting**.

In addition to this experiment, we further assessed the importance of T cell responses to immunosurveillance and maintenance of antigen expression using *K* and *K;Rag1*$^{-/-}$ mice. As expected, Lenti:mCh-SIIN/Cre tumors were significantly smaller than Lenti:mCh/Cre tumors in *K* mice, whereas Lenti:mCh-SIIN/Cre tumors were similar in size to Lenti:mCh/Cre tumors in *K;Rag1*$^{-/-}$ mice (**Fig. S1A**). Additionally, Lenti:mCh-SIIN/Cre tumors were more likely to have silenced antigen expression than Lenti:mCh/Cre tumors in *K* mice, whereas Lenti:mCh-SIIN/Cre tumors had similar frequency of antigen expression as their Lenti:mCh/Cre counterparts in *K;Rag1*$^{-/-}$ mice (**Fig. S1B**). These findings clearly demonstrate that the adaptive immune response is a necessary regulator of tumor immunoediting and significant pressure for mCherry-SIINFEKL silencing in the model. Thus, these additional data suggest that distinct perturbations in mCherry-SIINFEKL expression across tumor suppressor genotypes arise from differential regulation of adaptive immunity across each genetic context.

Finally, in the Tuba-seq approach, we included two sgRNA targets that serve as strong indicators that T cells restrict the outgrowth of Lenti:mCh/Cre and Lenti:mCh-SIIN/Cre tumors with increasing potency (PD-L1 and B2M). *PD-L1*^{KO} promoted a stepwise increase in tumor cell culling when tumors were initiated by Lenti:mCh/Cre and Lenti:mCh-SIIN/Cre, relative to tumors initiated by Lenti:Cre (**Fig. 6A**). Conversely, *B2M*^{KO} provided a selective advantage, allowing mCherry-expressing tumors to expand to greater extent than Lenti:Cre tumors that do not express mCherry (**Fig. S12B**). This effect increases in a stepwise fashion as the immunogenicity of the vector increases (**Fig. 6, A,C, and D**). The fact that losing antigen presentation aids Lenti:mCh/Cre and Lenti:mCh-SIIN/Cre tumor growth is a very strong indicator that

mCherry and SIINFEKL each elicit a functional T cell response that impedes tumor growth directly. We have clarified this point in the corresponding section of the text in the Results section (**Lines 288-290**). Together, these new experimental additions have enhanced the manuscript and addressed the Reviewer's concern.

Minor points

MP3.1: "The authors should better contextualize some findings that differ from established associations in human tumors. For instance:"

- **MP3.1a:** "Keap1 loss is typically associated with immune evasion in NSCLC (PMIDs 32312757, 37889752), yet here, Keap1KO tumors appear more immunogenic. This may be a question of timing of Keap1 loss, etc – but at minimum this should be discussed?"

  A3.1a: We agree with the Reviewer that the finding that *Keap1*^{KO} promotes immunosurveillance is surprising given its association with immunoevasion and PD-1/PD-L1 blockade resistance. A similar curiosity can be raised in the context of *Lkb1* loss, which is also associated with immune evasion and resistance to PD-1/PD-L1 blockade. Based on our data, *Lkb1*^{KO} promotes immune evasion in the mildly immunogenic context of Lenti:mCh/Cre (**Fig. 4, A and B**). However, upon expression of SIINFEKL, *Lkb1*^{KO} tumors become highly sensitized to immunosurveillance, as evidenced by the loss of neoantigen expression and presentation in this context, the reduction in tumor area observed both by histology and Tuba-seq, and the selection of tumors with failed *Lkb1* inactivation (**Fig. 3, D to G, Fig. 6, B to D**). We hypothesize that both *Lkb1* and *Keap1* loss likely drive potent immunoediting early in tumor development, and this in turn drives the immune evasion and checkpoint resistance that are characteristic of these two genotypes. Thus, both our data and clinical data make perfect sense. **This important point has been added to our discussion (Lines 409-423).** Future experiments assessing extended time points may uncover how these genotypes shape tumor–immune dynamics over time.

- **MP3.1b:** "Setd2 mutation is reported to be associated with immunotherapy response (PMID 39506116), suggesting Setd2 mutation improves anti-tumor immune responses but this is not observed in Fig 6B."

  A3.1b: We thank the Reviewer for highlighting this reference. Our results presented in **Fig. 6B** suggest that *Setd2*^{KO} tumors that express mCherry-SIINFEKL are sensitized to immunosurveillance relative to poorly immunogenic tumors that do not express mCherry-SIINFEKL. This is consistent with the indicated reference suggesting that *Setd2* mutations promote immunotherapy response. We have included this reference in the Introduction of the revised manuscript (**Line 57**).

- **MP3.1c:** "Lkb1 inactivation has been linked to neutrophil infiltration (PMID 26833127), but the data presented in Fig. 4A and 4B seem to suggest the opposite."

  A3.1c: Here again, we thank the Reviewer for highlighting this apparent discrepancy with a published report. In contrast to that report by the Wong group, we have published scRNA-seq data on individual lung tumors showing that LKB1 reactivation does not affect neutrophil recruitment to any measurable extent compared to LKB1-null tumors (PMID 35228570). In the publication from the Wong laboratory, the authors show that *Lkb1*^{KO} in KRAS-driven mouse models is associated with neutrophil recruitment which was measured by flow cytometry. In the current study using IHC, similar to our scRNA-seq on dissected tumors, we measure neutrophil presence in the direct vicinity of tumors. Moreover, we compare the infiltration of Ly6G^{Positive} neutrophils in tumors initiated by Lenti:mCh/Cre and Lenti:mCh-SIIN/Cre. In this experimental context, all tumors are expressing neoantigens (mCherry or mCherry-SIINFEKL). It is possible that the expression of the mCherry neoantigen impacts neutrophil infiltration beyond what might

result from Lenti:Cre induced tumors. Thus, the experimental platform and method of analysis used in our current or previous studies differ extensively from that used by Kwok Wong's group in the indicated publication. While it is not appropriate to make sound interpretations between the studies, we agree it is important to highlight these differences in the Results section, which we now have (**Lines 207-209**).

MP3.2: "Tuba-seq presents an exciting opportunity to simultaneously evaluate immune evasion of multiple tumor genotypes. However, it appears that antigenicity of individual tumors may be directly impacted by active immune responses against others – a particular concern when they share neoatigens (such as SIIN or Cas9). This is briefly addressed by the authors in comparing results in this experiment to those of "one-at-a-time" KOs of Lkb1/Setd2 – but seems like a more in depth discussion and greater analysis of TME is warranted."

A3.2: This is a very important point, and we thank the Reviewer for bring it to light. We agree with the Reviewer that the nature of pooled *in vivo* screening leaves open the possibility that individual tumor genotypes could drive changes to the immune system that may impact the immune response generated against other tumors regardless of their genotype. It is important to note that such field effects would only serve to limit our ability to detect cell autonomous, gene-specific effects in the heterogeneous pool. Thus, our detected Tuba-Seq *hits* must have a relatively outsized effect to modulate immune surveillance beyond any field effect. We considered this important concern in multiple ways:

The system is entirely controlled to detect intrinsic effects: We have used three independent lentiviral vectors each with distinct levels of immunogenicity. Thus, in comparisons across vectors, the vector is the only experimental variable. Additionally, there are TS^{WT} tumors that have only $KRAS^{G12D}$ and no tumor suppressor gene inactivated. Thus, in comparisons across tumor suppressor genotypes, the genotype is the only experimental variable. Finally, in our pooled Tuba-seq screens, all vectors initiate tumors in the same mouse. Thus, all experimental variables are internally controlled. Across the experiment, all tumors are subject to the same theoretical response to the cell transduction process used to initiate tumorigenesis. Therefore, changes in the propensity for tumors to grow is entirely due to their own immunogenicity and genotype.

The system includes sgRNA targets that are enriched/depleted as expected: Our screen was designed to include sgRNAs that target genes known to modulate immune responses (PD-L1 and B2M). KO of each of these control genes conferred the expected effect, as shown in **Fig. 6A**. For example, *PD-L1^{KO}* promoted immunosurveillance most strongly in the context of Lenti:mCh-SIIN/Cre, mildly in the context of Lenti:mCh/Cre, and not at all in the context of Lenti:Cre. These results align with the expectation that removing an inhibitory axis of T cell signaling induces T cell-mediated anti-tumor responses most potently in the setting were highly immunogenic neoantigens are expressed (Lenti:mCh-SIIN/Cre), less potently when mild T cell neoantigens are expressed (Lenti:mCh/Cre), and not at all when no T cell neoantigens are expressed (Lenti:Cre). On the other hand, KO of *B2M* had complex effects that were entirely expected. Due to the "missing self" mechanism, we expected that loss of MHC-I expression would limit tumor growth in non-immunogenic conditions, which is what we observe in Lenti:Cre tumors. However, as the selective disadvantage of expressing neoantigens rises in Lenti:mCh/Cre and then rises again in Lenti:mCh-SIIN/Cre tumors, we see now that there is a major selective advantage to losing MHC-I (and thus antigen presentation) (**Fig. 6A**). That this system can tease apart these complex effects of innate and adaptive immune contributions is remarkable.

Comparison of pooled approach and one-at a-time approach demonstrates similar outcomes: Both in the case of Tuba-seq and one-at-a-time gene KO experiments, *Lkb1* inactivation most strongly promoted immunosurveillance. In the single gene KO experiment, expression of mCherry-SIINFEKL led to the most potent reduction in tumor area compared to tumors expressing mCherry alone (**Fig. 3D**). Furthermore, the vast majority of tumors with *Lkb1^{KO}* lost neoantigen expression or presentation, suggesting strong immunoediting that was not observed in other tumor suppressor genotypes (**Fig. 3, F and G, Fig. S4, A to C**). There was also a 40% reduction in the proportion of LKB1-deficient tumors in the context of mCherry-SIINFEKL expression. Together, these findings support the Tuba-seq result that *Lkb1* inactivation strongly

promotes immunosurveillance, which supports the idea that the effects observed in Tuba-seq are primarily driven by tumor suppressor genotype and immunogenic context, rather than inter-lesion crosstalk (**Fig. 6, C and D**).

Finally, while inter-tumor crosstalk is important to consider and likely exists to some extent, our goal was to develop a system to assess tumor cell intrinsic effects of tumor genotype on immunogenicity and immunosurveillance. We demonstrate our system is able to detect changes in immunosurveillance driven by the intrinsic features of tumor genotype and immunogenicity despite potential inter-tumor crosstalk. Experiments to directly assess inter-tumor crosstalk effects would be better suited for a future study. We have included text in the Discussion to explore this important topic (**Lines 365-371**).

MP3.3: "The observation that Lkb1KO tumors exhibit extensive neoantigen silencing but relatively low tumor burden (Fig. 3D, 3F) is intriguing. One possibility is that these tumors experience intense immune pressure early and only a small fraction survive, perhaps with even small number of edited clones facing residual suppression from the immune system. It would be helpful if the authors could provide additional data (later timepoints or immune profiling of edited tumors) to clarify."

A3.3: Yes, we believe this is likely the case and thank the Reviewer for their excitement. In further support of this hypothesis, we provide additional data (**Fig. S2**) indicating that tumors with unsuccessful *Lkb1* inactivation were highly selected for in the context of SIINFEKL expression, which along with severe loss of neoantigen expression, points to early editing of tumors (**Fig. 3F, Fig. S2, A and B**). We highlight this in the Results section of the manuscript, when presenting the data of knockout efficiency (**Lines 152-156**). We also expand on this in the Discussion section, drawing a parallel between *Lkb1* and *Keap1* loss, which both have been shown to drive immune evasion and resistance to PD-1/PD-L1 blockade (**Lines 409-423**). We hypothesize that loss of either *Lkb1* or *Keap1* drives potent immunoediting early in tumor development, and this in turn drives the immune evasion and checkpoint resistance that are characteristic of these two genotypes (exactly as suggested by this Reviewer). We agree with the Reviewer that following how *Lkb1* and *Keap1* loss influence immunosurveillance and editing over time would be valuable to better understand why these genotypes promote resistance to checkpoint therapy and how to overcome this. While the inclusion of the new data quantifying the proportion of tumors with successful gene knockout provides additional evidence in support of our hypothesis, we hope to further investigate this in future studies.

MP3.4: "Given the availability of a previously published reversible Lkb1 knockout system (PMCID: PMC8885825) that the authors established, the team could also explore whether restoration of Lkb1 can reverse immune editing or if neoantigen loss is fixed. While this experiment may be beyond the scope of the current study, a brief discussion of this, and what the expected outcome may imply for immune escape, would be welcome."

A3.4: We thank the Reviewer for this very interesting experiment proposal, however given its complexity we agree that it is beyond the scope of our current study. We hypothesize that restoration of LKB1 early after tumor initiation could potentially reverse or lower a tumor's susceptibility to immunosurveillance, due to our observations that *Lkb1* inactivation potently induces early immunosurveillance. However, at later time points, once tumors with *Lkb1* inactivation have undergone high levels of editing, restoring LKB1 may no longer have an effect on immunosurveillance. This is supported by our finding in PMCID: PMC8885825 that restoration of LKB1 22 weeks after tumor initiation did not impact the abundance of infiltrating T cells or neutrophils by scRNA-seq on tumors. It would also be intriguing to use this model to determine how LKB1 restoration may alter resistance to immune checkpoint blockade. Such experiments could allow us to determine whether *Lkb1* loss itself drives resistance, or whether *Lkb1* loss drives potent immunoediting, which in turn drives resistance. This would be an interesting follow up study in future work.

REVIEWER 4

"In this manuscript, Adler and colleagues investigate how cancer driver mutations shape immunosurveillance during KRAS-driven lung tumorigenesis. By combining a set of increasingly immunogenic lentiviral vectors with CRISPR screening, the authors document how tumor suppressor gene inactivation regulates the level of immunosurveillance in emerging tumors. The authors show that, depending on tumor suppressor genotype, cancer cells use different strategies to escape the immune response and find that faster growing cancers are generally more susceptible to immunosurveillance. Overall, the manuscript is well-written and offers an interesting screening approach for the field to uncover new insights into how tumor genotypes influence cancer progression and immune evasion. However, a number of key controls are lacking and a few conclusions are overstated. The following points should be addressed prior to publication."

We thank the Reviewer for their insights and agree that our study highlights an innovative platform that can be utilized to determine how distinct cancer driver mutations may influence tumor immunosurveillance.

Major points

Q4.1: "The initial infection rate in the mouse lung should be assessed to allow a more meaningful comparison of tumor burden between different conditions (equal virus titer does not necessarily mean equal infection rates)."

A4.1: We agree with the Reviewer that mouse-to-mouse variability in lentiviral-based particle delivery is inevitable. To address this, we have changed our analysis to focus on individual tumor area instead of tumor burden in **Fig. 1** and **Fig. 3** such that the transduction efficiency and resultant tumor number should have a reduced impact on our interpretation of the results. We also note that our Tuba-seq experiments are all internally controlled, thus differences in transduction on individual mice have no impact on those results.

Q4.2: "Regarding the setup of their system, it seems the tumor burden is equal for all 3 vectors between weeks 8-12, only the Cre vector at week 16 is significantly different (Figure 1D). Given the n=4 and because this is an important feature in their system, do the authors have additional evidence that these vectors indeed result in differences in tumor growth (e.g. later time point)? Can the authors also quantify the number of tumors per lung, instead of only tumor burden? How many of these tumors are indeed malignant vs. adenomas, as assessed by pathology? Although the authors claim that tumors increase/do not increase from 8 to 16 weeks, no statistical test has been performed to compare 8 vs. 12 vs. 16 weeks."

A4.2: We thank the Reviewer for these helpful suggestions. All comparisons have been modified to assess 8 vs. 12 vs. 16 weeks for each immunogenic context, and use tumor area instead of tumor burden. Tumor number per mouse was not included due to the potential mouse-to-mouse variability in viral transduction efficiency that the Reviewer points out in **Q4.1**. These changes now highlight the significant increase in tumor size over time in Lenti:Cre tumors and demonstrate the lack of growth of tumors initiated by mCherry vectors between 8 and 16 weeks.

To provide further evidence that these vectors result in differences in tumor growth, we show by Tuba-seq that Lenti:mCh/Cre limits tumor size compared to Lenti:Cre, and that Lenti:mCh-SIIN/Cre further limits tumor size compared to both Lenti:Cre and Lenti:mCh/Cre (**Fig. 5, G-I, Fig. S10C**). These highly quantitative data suggest that mCherry alone promotes immunosurveillance, but to a lesser degree than mCherry-SIINFEKL.

With regard to the question of tumor grade in our system, previous experiments have shown that at 6 weeks post-transduction, *K* mice only harbor adenomas, with no overt adenocarcinomas (PMID 11751630). At 26 weeks, only around 30% of tumors in *K* mice are adenocarcinomas, with the rest remaining as adenomas. As a result, the vast majority of tumors assessed throughout the 16 week time course are likely adenomas. To address this point directly within our own system, we have assessed tumor

grade in tissue sections derived from mice that were transduced with the BC-sgTS11-IM library 12 weeks after tumor induction (representative scan of lobe in **Fig. 5C**). In *K* mice, we observed hyperplasias and adenomas, but no grade 3 adenocarcinomas. In *K;Cas9* mice, we observed hyperplasias and adenomas, with an additional average of 1 to 3 grade 3 adenocarcinomas per mouse (<1% of tumors). It is also important to note that within these models, tumor grade is not independent from tumor size, with only the largest tumors progressing to Grade 3. Because these lesions are infrequent, we do not believe that tumor grade could impact the overall interpretation of our results. Nonetheless, the Reviewer's point is important and would be very interesting to assess in future work. We have included new text in the Results section associated with this notion (**Lines 256-257**).

Q4.3: "The authors make the claim that there is a difference in the propensity to select for mCherry-SIIN silencing between Lkb1KO, Setd2KO, or Rb1KO tumors. Although the phenotype seems convincingly strong in Figure 3F, these claims are based on a single sgRNA for each tumor suppressor. The authors validate the on-target effect of these sgRNAs by IHC and point to previously published work, however, this does not confirm specificity. A second sgRNA should be used for Lkb1 KO, Setd2 KO, and Rb1 KO to further ensure that the strong difference in phenotype is not due to an unspecific off-target effect of the sgRNA."

A4.3: Here we have used a single sgRNA sequence to target Lkb1 and Rb1 and two distinct sgRNA sequences targeting Setd2. We have used these sgRNA sequences alongside additional sgRNAs targeting the same genes in numerous publications (PMIDs: 41188600, 28530655, 37828026, 33707235, 33608386, 29610476, 34215621, 40501771). In each instance, we found that each sgRNA results in highly reproducible effects from experiment to experiment and between sgRNAs targeting the same gene. Based on the robustness of phenotypes that result from this approach we have confidence using limited number of sgRNAs targeting genes of interest. This is coupled with the exceedingly low probability that an off-target effect that impacts the immunogenicity of a tumor. Regardless, there is no doubt that this persists as a diminishingly small limitation on the interpretations and we have addressed this in the Results section (**Lines 244-246**). We hope that the Reviewer will agree that the wholesale repetition of our study with additional sgRNA sequences is beyond what we could reasonably accomplish within the review period for this manuscript and would not change overall conclusions.

Q4.4: "Similar to point 1, the authors should provide data on the library representation at the beginning of the CRISPR screen in Figures 5 & 6."

A4.4: Thank you for pointing out this obfuscation. We originally included this in the Methods section, but now reference it during the presentation of the experimental results. Please refer to **Fig. S10**, previously **Fig. S14**, where we show the plasmid library representation, and how this compares to library representation in *K* mice. We further elaborate on this in the Methods section, as a detailed understanding of library representation is a strength of the Tuba-seq approach.

Q4.5: "Figure 6C/D: An important conclusion the authors reach in this paper is the correlation between strong growth promotion and sensitivity to immunosurveillance with different tumor suppressor genotypes. Considering Figure 6C and S13A, one might draw the alternative conclusion that highly immunogenic conditions set a ceiling/maximum for tumor size that is independent of tumor suppressor genotype. This leads to the same observation, as tumors that grow faster in the absence of this ceiling would have "relative higher sensitivity", although the ceiling is the same for all TSGs. The authors should provide further evidence that supports their hypothesis or discuss this alternative in the text."

A4.5. The Reviewer raises an interesting idea that mCherry-SIINFEKL expression may set a "universal ceiling" for tumor size that is independent of tumor genotype. However, several pieces of evidence in our study suggest this is not the case. First, that *Lkb1*^{KO} tumors are most potently selected against **when tumors are initiated** by Lenti:mCh-SIIN/Cre but not Lenti:mCh/Cre suggests that genotype-specific patterns of immunosurveillance begin **early after tumor initiation and before any ceiling of tumor size**

could be reached (**Fig. S2, A and B**). In poorly immunogenic tumors, *Lkb1*^{KO} is highly advantageous, and thus the selection against these tumors in a highly immunogenic context suggests specific and robust tumor culling in this genotype compared to *Setd2*^{KO} and *Rb1*^{KO} (**Fig. S2, C to F**).

In addition, while highly immunogenic conditions may impose an upper limit on tumor size, this framework does not fully account for the genotype-specific effects observed in our study. Notably, many *Trp53*- and *Rbm10*-deficient tumors exceed this apparent size threshold, whereas *Lkb1*- and *Setd2*-deficient tumors show disproportionate sensitivity to immunosurveillance under highly immunogenic conditions (Fig. 6, B to D, Fig. S14A). These findings argue against a universal ceiling that is independent of tumor suppressor genotype and instead support differential tumor proliferation rates as the most parsimonious explanation for the observed relationship between genotype and immune-mediated tumor control. However, we believe this alternative explanation is important and have now addressed it in the Discussion (**Lines 377-384**).

Q4.6: "Based on Figure 6B, the authors claim that only inactivation of Trp53, Rbm10 and Smad4 promoted resistance to tumor immunosurveillance. To strengthen this claim, the authors should carry out statistical testing for the comparisons in Figures 6A-B. For example, the Apc KO tumor size seems highly variable in the mCherry-SIIN/Cre condition."

A4.6: In **Fig. 6B**, genotypes are ordered from left to right according to their proliferative capacity in the Lenti:Cre context, with the most proliferative on the left and the least on the right. In the presence of immunogenic neoantigens, there was a stronger reduction in mean tumor size from left to right, with some exceptions noted in **Fig. 6D**. The pattern suggests that more proliferative tumors are subject to stronger immunosurveillance, while the exceptions highlight certain genotypes that are more or less resistant to immunosurveillance, independent of the proliferation effect. **To address the Reviewer's suggestion of using statistical testing to bolster our claims, we marked which gene knockouts significantly alter sensitivity or resistance to immunosurveillance in Fig. 6D.** Genes with an FDR controlled p-value of <0.1 are flagged as significantly deviating from the expected regression line.

Minor points

MP4.1: "It is not mentioned which statistical test was used in Figure 1F. In addition, Figure 1F and Figure 3F suggest different percentages of mCherry negative tumors in the mCh-SIIN/Cre control group: 1F states 60 % mCherry negative tumors at week 12, in 3F it is 40 %. The authors should comment on this."

A4.1: The statistical test used in **Fig. 1F** is a chi-squared test, which is now indicated in the figure legend. As the Reviewer indicated, we saw a higher percentage of mCherry negative tumors in the initial Lenti:mCh-SIIN/Cre cohort than we saw in the similar cohort in **Fig. 3F**. We have increased the sample size for the analysis shown in **Fig. 3F** and still see the same difference. While we cannot say for sure, this distinction could arise from the timing of IHC analysis, natural experimental variation or perhaps most likely due to different mouse lines used in Figure 1 (*Kras*$^{LSL\text{-}G12D/+}$) and in Figure 3 (*Kras*$^{LSL\text{-}G12D/+}$; *Rosa26*$^{LSL\text{-}Cas9:GFP/LSL\text{-}Cas9:GFP}$). Regardless, the comparisons tested are only within each experiment and not between experiments which we believe is the most valid.

MP4.2: "The authors have used student's t-test to compare across groups (ie. Fig. 1D, 2C, 3D). Since they are often comparing more than 2 groups, was a multiple comparison correction used? Please also indicate the type of correction."

A4.2: Many thanks to the Reviewer for the comment. We have redone all the statistical analyses throughout the paper and converted to ANOVA testing where appropriate. While some of the p values are changed due to the multiple hypothesis testing penalties associated with ANOVA and from the extensive additional sample numbers added to increase experimental rigor, none of the overall conclusions are affected. We have indicated the exact statistical analyses and type of multiple comparison corrections used

in the figure legends for each panel.

MP4.3: “Figure 2C/D: As inferred from Figure 1C, there is a considerable difference in tumor burden between the different groups at week 16. It is conceivable that this difference in absolute tumor burden in the lung also influences the immune cell infiltration in a way not directly related to the vectors. The authors should comment if the comparison of immune cell infiltration at this timepoint can be used to compare the immunogenicity of the vectors.”

A4.3: To thoroughly address this, we have changed our analysis to focus on how immune infiltration changes over time in each immunogenic context. Looking at the immune infiltration in each of the mCherry vectors, we see a sharp and significant increase in $CD45^{Positive}$ cells at 16 weeks compared to 8 or 12 weeks despite no change in tumor growth between these time points (**Fig. 1, D and F, Fig. S1C**). This phenomenon is not observed in Lenti:Cre tumors, which have a significant increase in tumor area at 16 weeks. We also show this in a new supplementary figure, **Fig. S1C**. Using simple linear regressions, we see that $CD45^{Positive}$ immune infiltration does not significantly change over time in Lenti:Cre tumors, whereas there is a significant increase in $CD45^{Positive}$ immune infiltration in both Lenti:mCh/Cre and Lenti:mCh-SIIN/Cre tumors, as exhibited by the positive slope and small p-values (>0.0001). If tumor size or burden were to strongly effect immune infiltration, the slope of the Lenti:Cre line in **Fig. S1C** would likely deviate more significantly from zero in the negative direction, and the p-value would be significant. Although we appreciate the point raised by the Reviewer that certain factors like tumor area or burden could impact immune infiltration, there seems to be no detectable effect in our system at the 16 week time point. Therefore, we believe that our comparisons at 16 weeks can indeed be used to support our claims that we have generated vectors that model different levels of immunogenicity.

MP4.4: **“**Lines 113-117: there’s an increase in the number of immune cells for Lenti::mCh/Cre and Lenti::mCh-SIIN/Cre relative to Lenti::Cre. If the authors want to claim an increase in immune infiltration over time (as described in Line 116), they should compare across weeks, not between conditions.”

A4.4: Similar to MP4.3, we fixed this comparison to reflect change over time for each vector in **Fig. 2, C and D** and **Fig. S1, C and D**.

MP5: “Line 140: “Unexpectedly, Lkb1KO, Setd2KO or Rb1KO did not significantly increase tumor burden compared to TSWT tumors when initiated with Lenti:mCh-SIIN/Cre.” In the corresponding Figure 3D, no statistical test was used to determine statistical significance between the tumor suppressors and TSWT in mCherry-SIIN tumors.”

A4.5: We have changed these data to assess individual tumor area instead of tumor burden, and we use a one-way ANOVA testing with a Šidák correction to test statistical significance, as denoted in the new figure legend for **Fig. 3D**.

MP4.6: “Figures 5B and 5F could be combined into one to enhance clarity for the reader.”

A4.6: Thank you for pointing this out. We have combined these into one figure (**Fig. 5B**).

MP4.7: “It would be interesting to show if the relative resistance trend described in Figure 6 also applies to tumor initiation frequency/tumor burden, as supposed to LN tumor size. The total number of tumors for each sgRNA can be inferred from the tumor barcodes.”

A4.7: We thank the Reviewer for their valuable suggestion. We have included a new plot which shows that the TS genotypes with higher growth rates in tumors initiated by Lenti:Cre have lower tumor numbers when initiated by Lenti:mCh/Cre or Lenti:mCh-SIIN/Cre, demonstrating that more rapidly proliferating tumors have lower initiation rates as the tumor immunogenicity increases to a mildly or highly immunogenic context (**Rebuttal Fig. 1**). Notably, $B2M^{KO}$ significantly promoted tumor initiation only in the context of

mCherry-SIINFEKL expression. Unlike the relationship between Relative Resistance and the size of Lenti:Cre$^{BC\text{-}sgTS11\text{-}IM}$ tumors, the reduction in tumor initiation appeared to occur across immunogenic contexts and thus does not appear to be related to tumor immunogenicity. We have decided not to include these data in the current manuscript for better clarity.

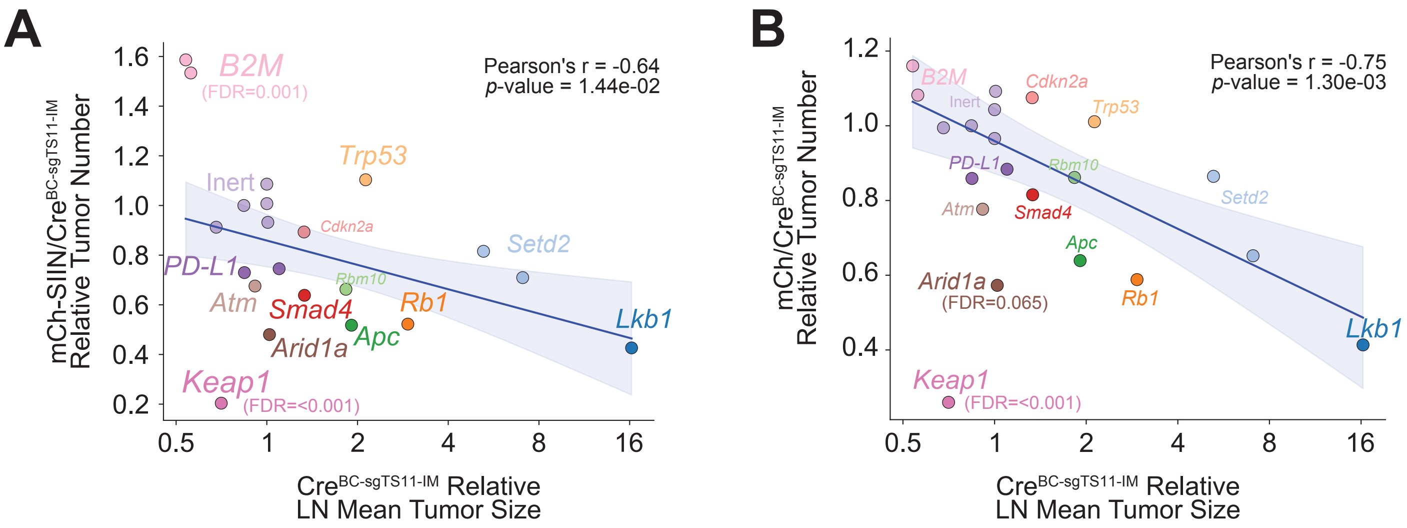

Rebuttal Fig. 1. Plots depicting relative tumor number across TS genotypes in tumors initiated by Lenti:mCh-SIIN/Cre$^{BC\text{-}sgTS11\text{-}IM}$ (**A**) or Lenti:mCh/Cre$^{BC\text{-}sgTS11\text{-}IM}$ (**B**) versus relative LN Mean tumor size of tumors initiated by Lenti:Cre$^{BC\text{-}sgTS11\text{-}IM}$.

MP4.8: "Figure S11 is not very intuitive to understand and has a few typos ("resistence" instead of "resistance")"

A4.8: We apologize for the grammatical issues in this figure; they have been resolved.

MP4.9: "A period is missing in line 358."

A4.9: We have resolved this issue.

MP4.10: " "That we see major differences between genotypes and between different immunogenic contexts all within the same organ environment indicates that significant cancer cell-autonomous differences exist that are driven by biological effects of tumor genotype." Please rephrase."

A4.10: We thank the Reviewer for their suggestion. We have rewritten that paragraph, and the new corresponding text now reads: "Though the pooled nature of Tuba-seq mimics tumor genetic heterogeneity that exists in human cancers, inter-tumor crosstalk or field effects, where a specific tumor clone with a given genotype affects immunosurveillance of other tumor clones, is possible or perhaps even likely. However, such field effects would only serve to limit the ability to detect cell autonomous gene-specific effects in the heterogeneous pool, suggesting that our detected Tuba-Seq *hits* must have a relatively outsized effect to modulate immune surveillance." (**Lines 365-371**).

MP4.11: "In the discussion, it may be worth mentioning the limitations of using extrinsically introduced immunogenic genes such as SIINFEKL and their biological relevance in a more "natural" setting in terms of tumor formation."

A4.11: We agree with the Reviewer that SIINFEKL is a highly immunogenic model antigen and does not fully reflect the average immunogenicity of endogenous tumor neoantigens. However, GEMMs of LUAC have inherently low tumor mutational burden and fail to respond to immune checkpoint inhibitors, making them poorly suited to study neoantigen-driven tumor–immune dynamics. In this context, SIINFEKL provides a useful surrogate for the presence of immunogenic neoantigens. Supporting this, Alban *et al.* (PMID: 39349627) demonstrated that although only a minority of predicted neoantigens are immunogenic, those that are can be sufficiently potent to drive clonal elimination and immunoediting during PD-1 blockade. Thus, the use of SIINFEKL enables GEMMs to capture aspects of tumor–immune interactions that are otherwise absent in these models but are relevant to human LUACs with higher mutational burden. We have added text mentioning the rationale for and limitation of using SIINFEKL as a model neoantigen in our system to the results section (**Lines 87-90**).

REVIEWERS' COMMENTS

Reviewer #2:

I co-reviewed this manuscript with one of the reviewers who provided the listed reports. This is part of the Nature Communications initiative to facilitate training in peer review and to provide appropriate recognition for Early Career Researchers who co-review manuscripts.

We thank the Reviewer for their contribution to the revision of our manuscript.

Reviewer #3:

Q3.1: I appreciate the thoughtful revisions and the substantial effort made to address the reviewers' suggestions and concerns, which have strengthened the manuscript. My only minor comment is that there appears to be a truncated sentence around lines 367–368, please consider revising for the final version.

A3.1: We thank the Reviewer for their recognition of our efforts to improve our manuscript. We have revised the sentence around lines 367-368 to now read: "While the pooled Tuba-seq approach mimics the genetic heterogeneity seen in human cancers, it also allows for potential inter-tumor crosstalk or field effects, in which one tumor clone's genotype may influence the immunosurveillance of neighboring clones." This can be found on lines 365-368.

Reviewer #4:

The authors have satisfactorily addressed most of our concerns. The following remaining points should be considered for the final manuscript.

Many thanks to the Reviewer for these helpful comments. We have revised our manuscript to address these suggestions.

1. Regarding answer 4.2.: It seems that the text has not been updated correctly. The statement "As expected, tumors initiated with Lenti:Cre increased in size over time (Fig. 1, C and D). In contrast, tumors initiated with either Lenti:mCh/Cre or Lenti:mCh-SIIN/Cre did not increase in size from 8 to 16 weeks" (lines 96–98) does not appear to correspond to Figure 1D. Based on Figure 1D, mCh-SIIN/Cre tumors show a statistically significant increase in tumor area at 16 weeks. The text should be updated to reflect the data presented in the figure.

A4.1: Thank you to the Reviewer for highlighting this error. We have modified the wording to reflect that tumors initiated by Lenti:mCh-SIIN/Cre grew at a slower rate than those initiated by Lenti:Cre.

2. The response regarding the use of only a single sgRNA is not convincing and several of the cited studies do also not include validation with multiple sgRNAs, limiting the support for this claim. While we recognize that experimentally addressing this concern at this point would require additional work, including the repetition of multiple experiments, we think that this limitation should be explicitly acknowledged in the Discussion section.

A4.2: We agree with the Reviewer that this is a point that should be addressed. We have added a sentence (lines 421-424) to highlight a need to further validate findings across multiple sgRNAs per gene.

3. The statement “In TSWT and Lkb1KO tumors, mCherry-SIINFEKL expression reduced tumor area” (Lines 149–150) may be misleading, as Figure 3D does not show a statistically significant decrease. We recommend revising the wording to more accurately reflect the data.

> **A4.3:** We have changed the wording in line 150 to reflect that there was a distinct, but non-significant reduction in tumor area in TS^{WT} and $Lkb1^{KO}$ tumors. Thank you to the Reviewer for pointing this out.

4. We appreciate the authors’ effort to address the point 4.6. through the statistical analysis presented in Figure 6D. However, our concern relates specifically to the individual genes shown in Figures 6A and 6B. For example, Apc relative LN mean tumor size Cre vs. mChSIIN/Cre. For full transparency, statistical comparisons between the relevant groups within each gene should be provided for Figure 6A/B. Conducting analysis only at the level of relative resistance (as in Figure 6D, comparing across all genes) does not fully resolve this point.

> **A4.4:** Thank you to the Reviewer for their clarification regarding Figs. 6A and B. For Fig. 6A and B, Fig. S13A and C and Fig. S14C, we have added FDR values to show the statistical significance in tumor growth across immunogenic contexts for each gene of interest. We believe this satisfies the Reviewer’s request.
